# Silicon(i) chemistry: the NHC-stabilised silicon(i) halides Si_2_X_2_(Idipp)_2_ (X = Br, I) and the disilicon(i)-iodido cation [Si_2_(I)(Idipp)_2_]^+^†
†Electronic supplementary information (ESI) available: Syntheses and analytical data of **2-Cl**, **2-Br**, **2-I** and **3**, illustrations of the ^1^H, ^13^C and ^29^Si NMR spectra of **2-Br**, **2-I** and **3**, results of the analysis of the dynamic process of **3** in solution and details of the quantum chemical calculations of **2-Br**, **2-I**, SiBr_2_(Idipp) and the cation in **3**. CCDC 1414787–1414789. For ESI and crystallographic data in CIF or other electronic format see DOI: 10.1039/c5sc02681d. NHC = N-heterocyclic carbene; Idipp = C[N(dipp)CH]_2_, dipp = C_6_H_3_-2,6-*i*Pr_2_.


**DOI:** 10.1039/c5sc02681d

**Published:** 2015-08-17

**Authors:** Marius I. Arz, Daniel Geiß, Martin Straßmann, Gregor Schnakenburg, Alexander C. Filippou

**Affiliations:** a Institut für Anorganische Chemie , Rheinische Friedrich-Wilhelms-Universität Bonn , Gerhard-Domagk-Straße 1 , 53121 Bonn , Germany . Email: filippou@uni-bonn.de

## Abstract

An efficient method for the synthesis of the NHC-stabilised Si(i) halides Si_2_X_2_(Idipp)_2_ (**2-X**, X = Cl, Br, I; Idipp = C[N(C_6_H_3_-2,6-*i*Pr_2_)CH]_2_) was developed, which involves the oxidation of Si_2_(Idipp)_2_ (**1**) with 1,2-dihaloethanes. Halogenation of **1** is a diastereoselective reaction leading exclusively to a racemic mixture of the *RR* and *SS* enantiomers of **2-X**. Compounds **2-Br** and **2-I** were characterised by single-crystal X-ray crystallography and multinuclear NMR spectroscopy, and their electronic structures were analysed by quantum chemical methods. Dynamic NMR spectroscopy unraveled a fluxional process of **2-Br** and **2-I** in solution, which involved a hindered rotation of the NHC groups about the Si–C_NHC_ bonds. Iodide abstraction from **2-I** by [Li(Et_2_O)_2.5_][B(C_6_F_5_)_4_] selectively afforded the disilicon(i) salt [Si_2_(I)(Idipp)_2_][B(C_6_F_5_)_4_] (**3**). X-ray crystallography and variable-temperature NMR spectroscopy of **3** in combination with quantum chemical calculations shed light on the ground-state geometric and electronic structure of the [Si_2_(I)(Idipp)_2_]^+^ ion, which features a Si=Si bond between a trigonal planar coordinated Si^II^ atom with a Si–I bond and a two-coordinate Si^0^ center carrying a lone pair of electrons. The dynamics of the [Si_2_(I)(Idipp)_2_]^+^ ion were studied in solution by variable-temperature NMR spectroscopy and they involve a topomerisation, which proceeds according to quantum theory *via* a disilaiodonium intermediate (“π-bonded” isomer) and exchanges the two heterotopic Si sites.

## Abstract 2

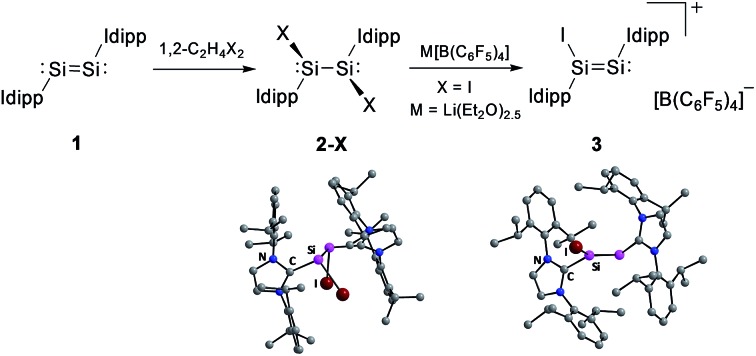
An efficient method for the synthesis of the NHC-stabilised Si(i) halides Si_2_X_2_(Idipp)_2_ (**2-X**, X = Cl, Br, I) was developed, which involves the oxidation of Si_2_(Idipp)_2_ (**1**) with 1,2-dihaloethanes. Iodide abstraction from **2-I** afforded the unprecedented silicon(i) salt [Si_2_(I)(Idipp)_2_][B(C_6_F_5_)_4_] (**3**).

## Introduction

The molecular chemistry of silicon has witnessed remarkable progress in recent years following the discovery that N-heterocyclic carbenes (NHCs) are particularly suitable Lewis-bases for the thermodynamic and kinetic stabilisation of highly reactive, low-valent silicon species.^1^ Appealing examples substantiating this development include the Si(0) compounds Si_2_(NHC)_2_ (NHC = C[N(dipp)CH]_2_ (Idipp) and C[N(dipp)CH_2_]_2_ (SIdipp); dipp = C_6_H_3_-2,6-*i*Pr_2_)^2^ and Si(bNHC) (bNHC = chelating bis-N-heterocyclic carbene),^3^ the Si^I^ chloride Si_2_Cl_2_(Idipp)_2_,2*a* the NHC-stabilised Si^II^ compounds SiX_2_ (X = Cl–I),^4^ Si(X)R (X = Cl, Br, H; R = aryl, amino, silyl),^5^ Si(R)(SiR=SiR_2_) (R = C_6_H_2_-2,4,6-iPr_3_)^6^ and 1-silacyclopenta-2,4-dienylidenes SiC_4_R_4_ (R = Ph, NEt_2_),^7^ and the NHC-trapped silyliumylidene cations [SiR]^+^ (R = I, aryl)4d,5e,^8^ and Si^2+^ ions.4*d* More recently, NHC-stabilised phosphasilenylidenes Si=PR (R = C_6_H_2_-2,4,6-*t*Bu_3_)^9^ and disilavinylidenes Si=Si(Br)R (R = C_6_H_2_-2,6-{CH(SiMe_3_)_2_}_2_-4-*t*Bu) were also isolated, fortifying the binding capacity of NHCs.^10^ All these compounds offer new avenues of chemical exploration with potential applications in both molecular chemistry and materials science due to their functional versatility originating from the simultaneous presence of many reactive sites, such as the Si lone pairs, the silicon–halogen bonds or the displaceable NHC groups. In fact, the Si^II^ halides SiX_2_(NHC) (X = Cl–I, NHC = Idipp and SIdipp) and SiCl(R)(NHC) (R = C_6_H_3_-2,6-Tip_2_, Tip = C_6_H_2_-2,4,6-*i*Pr_3_; NHC = C[N(Me)CMe]_2_) were shown to be valuable starting materials, which paved the way to new classes of unsaturated silicon compounds, including silylidyne complexes, zwitterionic silylidene complexes, metallosilylenes and metallosilanones.^11^

**Chart 1:**
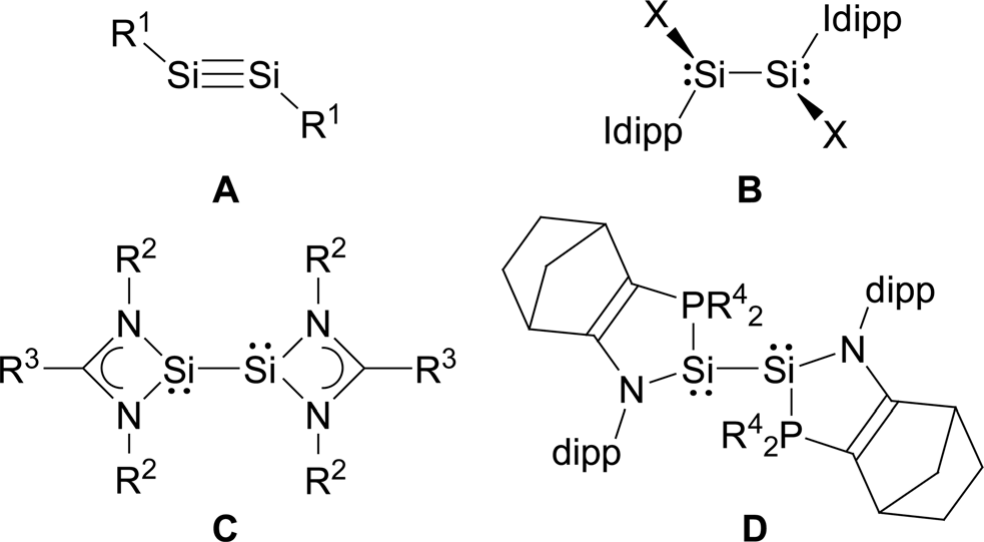
Important classes of silicon(i) compounds (X = Cl; Idipp = C[N(dipp)CH]_2_; R^1^ = alkyl, aryl, silyl; R^2^ = *t*Bu, R^3^ = Ph or R^2^ = dipp, R^3^ = C_6_H_4_-4-*t*Bu; R^4^ = *t*Bu). Formal charges are not included for simplicity.

In comparison, the chemistry of NHC-stabilised silicon(i) halides (Chart 1, **B**) has not been explored so far. This can probably be attributed to the severely limited access to this very reactive class of compounds, as revealed by the very low yield synthesis (6.1%) of the only presently known example Si_2_Cl_2_(Idipp)_2_ (**2-Cl**) upon the reduction of SiCl_4_(Idipp) with C_8_K.2*a* Compounds **B** can be viewed as bis(NHC) adducts of the disilynes Si_2_R_2_ (**A**, Chart 1), the chemistry of which has flourished since the isolation of the first thermally stable compounds in 2004.^12^ The NHC-stabilised Si^I^ halides **B** bear as their amidinato (**C**, Chart 1)^13^ or (phosphino)enamido-stabilised congeners (**D**, Chart 1),^14^ a reactive Si–Si single bond and a lone pair of electrons at each silicon atom, but contain beyond displaceable halide and NHC substituents, which offer additional dimensions of reactivity.^15^ In the present work, an efficient synthesis of NHC-stabilised Si^I^ halides Si_2_X_2_(Idipp)_2_ (**2-X**, X = Cl, Br, I) is reported, facilitating the exploration of their reactivity. Moreover, iodide abstraction from **2-I** is demonstrated to provide access to an unprecedented Si(i) salt containing the NHC-trapped [Si_2_I]^+^ cation.

## Results and discussion

**Scheme 1:**
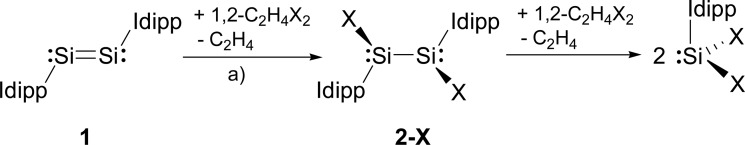
Synthesis of the Si(i) halides **2-X** (X = Cl, Br, I) and further oxidation to the Si(ii) halides SiX_2_(Idipp) (Idipp = C[N(dipp)CH]_2_); a) X = Cl: toluene, –30 °C; X = Br: toluene, –45 °C; X = I: THF, –70 °C. Formal charges are not included for simplicity.

We presumed that halogenation of the Si–Si double bond of the disilicon(0) compound (Idipp)Si=Si(Idipp) (**1**)2*a* might be a suitable reaction for the synthesis of the target silicon(i) halides, analogous to the well-known olefin halogenation in carbon chemistry.^16^ The 1,2-dihaloethanes 1,2-C_2_H_4_X_2_ (X = Cl, Br, I) proved to be particularly suitable reagents to accomplish this transformation. In fact, the addition of a stock solution of 1,2-C_2_H_4_X_2_ to a dark red solution of **1** in THF or toluene at low temperature was accompanied by a rapid color change to red-orange and gas evolution (ethene), and selectively afforded the Si^I^ halides **2-X** (X = Cl, Br, I) in moderate to very good yields (**2-Cl**: 49%; **2-Br**: 98%; **2-I**: 61%) (Scheme 1).† Precise adherence to a 1 : 1 stoichiometric ratio of the reactants and a slow addition rate proved to be essential for a selective conversion of **1** to **2-X**. This avoids further oxidation of **2-X** by 1,2-C_2_H_4_X_2_ to the Si^II^ halides SiX_2_(Idipp) (X = Cl, Br, I) (Scheme 1), which are difficult to separate from **2-X** due to their similar solubilities.4a,b,d

Halogenation of **1** by 1,2-C_2_H_4_X_2_ is a highly diastereoselective *cis*-addition leading exclusively to a racemic mixture of the *RR* and *SS* stereoisomers of **2-X** (Scheme 1). No evidence for the formation of the *meso* diastereoisomer (*trans*-addition product) was found, which, according to quantum chemical calculations at the B97-D3/**I** level of theory,^17^ is thermodynamically less stable than the *RR*/*SS* stereoisomers by 57 kJ mol^–1^ (see ESI,† Section 5.1). In comparison, halogenation of the diphosphene RP=PR (R = C(SiMe_3_)_3_) with Cl_2_ was reported to give exclusively the *meso* diastereomer.^18^,^19^

An alternative approach to the Si^I^ halides **2-X** was also investigated, which involved comproportionation of the Si^0^ compound **1** with SiX_2_(Idipp). Whereas no reaction between **1** and SiBr_2_(Idipp) was observed at room temperature, heating a 1 : 2 mixture of **1** and SiBr_2_(Idipp) in toluene at 85 °C afforded the Si^I^ bromide **2-Br**, as confirmed by NMR spectroscopy. However, conversion to the comproportionation product competed with the slow decomposition of **2-Br** occurring under the same conditions (*vide infra*), leading finally to a mixture of **2-Br**, SiBr_2_(Idipp) and Idipp. Whereas Idipp could easily be removed, separation of **2-Br** from SiBr_2_(Idipp) proved to be difficult due to their similar solubility preventing the isolation of **2-Br** in high-yield and pure form.

The silicon(i) halides **2-X** were isolated as vermillion, extremely air-sensitive solids, which immediately decolourised when in contact with air, but can be stored indefinitely at room temperature under an atmosphere of argon. Compounds **2-Br** and **2-I** are thermally quite robust in the solid-state and decompose upon heating at a temperature (190 °C) similar to that previously reported for **2-Cl** (184 °C).2*a* However, in toluene solution, **2-Br** starts to decompose at a much lower temperature (at 85 °C, *ca.* 10% decomposition within 2 h), and the decomposition leads to Idipp, SiBr_2_(Idipp) and an unknown toluene-insoluble solid (see Fig. S5 in the ESI†).

Compounds **2-Br** and **2-I** are the first molecular silicon(i) bromide and iodide to be reported and were comprehensively characterised by single-crystal X-ray crystallography, NMR spectroscopy and quantum chemical calculations.†

**Fig. 1:**
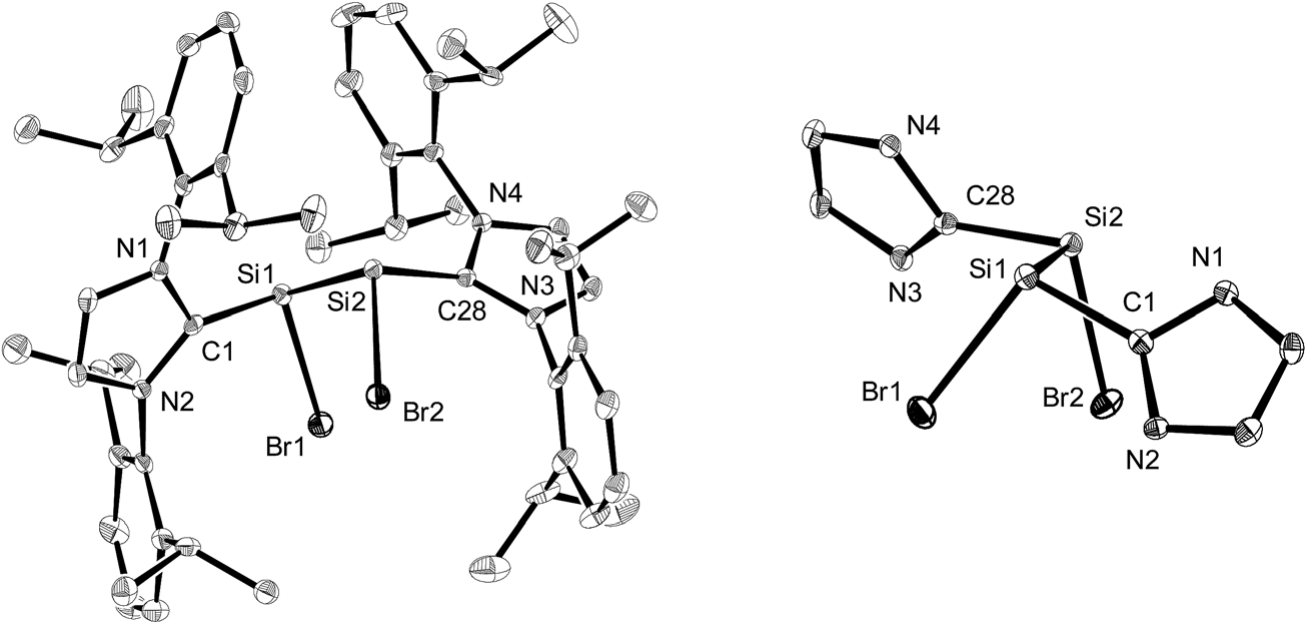
Left: Diamond plot of the molecular structure of **2-Br**·0.5(*n*-C_6_H_14_) in the single crystal. The thermal ellipsoids are set at 30% probability at 123(2) K, and the hydrogen atoms and *n*-hexane molecules are omitted for clarity. Right: View of the **2-Br** molecule along the Si–Si bond axis illustrating the synclinal arrangement of the Br atoms and the antiperiplanar orientation of the NHC groups (N-bonded dipp substituents are omitted for clarity). Selected bond lengths [Å], bond angles [°] and torsion angles [°] for **2-Br**·0.5(*n*-C_6_H_14_): C1–Si1 1.940(3), Si1–Br1 2.3602(8), Si1–Si2 2.385(1), Si2–Br2 2.3677(9), Si2–C28 1.936(3); C1–Si1–Br1 102.22(9), C1–Si1–Si2 97.87(9), Br1–Si1–Si2 103.78(4), Si1–Si2–Br2 104.17(4), Si1–Si2–C28 96.74(9), Br2–Si2–C28 101.42(9); C1–Si1–Si2–C28 161.5(1), Br1–Si1–Si2–Br2 –46.81(4).

The molecular structures of the *n*-hexane semisolvates **2-Br**·0.5(*n*-C_6_H_14_) and **2-I**·0.5(*n*-C_6_H_14_) were determined by single-crystal X-ray diffraction (Fig. 1, Table 1 and Fig. S23 in the ESI†). All compounds **2-X** (X = Cl–I) feature two stereogenic trigonal pyramidal silicon centers of the same configuration and display similar bonding parameters (Table 1). The halogen substituents of **2-X** adopt a synclinal conformation and the sterically more demanding Idipp groups adopt an antiperiplanar conformation, as indicated by the X–Si–Si–X torsion angles (**2-Cl**: –46.5(1)°,2*a***2-Br**: –46.81(4)°, **2-I**: 50.46(3)°) and C_NHC_–Si–Si–C_NHC_ torsion angles (**2-Cl**: –162.9(3)°,2*a***2-Br**: 161.5(1)°, **2-I**: –160.31(9)°), respectively (Fig. 1). The degree of silicon pyramidalisation (DP) of the Si^I^ halides ranges from 57–64% and is considerably smaller than that of the Si^II^ halides SiX_2_(Idipp) (DP = 70–78%) (Table 1).^20^ This suggests a lower s-character of the lone pair orbitals in **2-X** compared to those in SiX_2_(Idipp), which was confirmed by comparative NBO analyses (see Tables S6 and S7 in the ESI†). Furthermore, these analyses indicate a higher s-character of the Si hybrid orbitals employed in the bonding to the NHC groups in **2-X**, providing a rationale for the observed shortening of the Si–C_NHC_ bonds of **2-X***versus* those in SiX_2_(Idipp) (Table 1). These trends follow the predictions of Bent's rule made for a replacement of one halogen atom in SiX_2_(Idipp) by the more electropositive substituent SiX(Idipp).^21^ The Si–Si bond lengths of **2-X** (**2-Cl**: 2.393(3) Å,2*a***2-Br**: 2.385(1) Å, **2-I**: 2.3909(9) Å) are slightly longer than that in α-Si (2.352 Å)^22^ and lie in-between those of the amidinato-substituted Si^I^ compounds **C** (2.413(2) Å and 2.489(2) Å)^13^ and the (phosphino)enamido containing compound **D** (2.331(1) Å) (Chart 1).^14^ Remarkably, a plot of the Si–Si bond length of the Si^I^ compounds **B**, **C** and **D***versus* the sum of the bond angles at silicon revealed a good linear correlation, with the Si–Si bond length decreasing upon an increase in the sum of the bond angles (decreasing pyramidalisation) at the silicon atoms (see Fig. S25 in the ESI†).

**Table 1:** Comparison of selected bonding and NMR spectroscopic data of **1**, **2-X** and SiX_2_(L) (X = Cl, Br, I; L = Idipp) (bond lengths [Å], bond angles [°], sums of angles at silicon (∑_Si_) [°], degrees of pyramidalisation (DP) [%], and chemical shifts (*δ*) [ppm])

	**1** ^*a*^	**2-Cl** ^*a*^	**2-Br**	**2-I**	SiCl_2_(L)^*b*^	SiBr_2_(L)^*c*^	SiI_2_(L)^*d*^
Si–Si	2.229(1)	2.393(3)	2.385(1)	2.3909(9)			
Si–C_NHC_	1.927(2)	1.939(6)	1.940(3)	1.943(2)	1.985(4)	1.989(3)	1.984(7)^*g*^
		1.929(7)	1.936(3)	1.939(2)			
Si–X		2.161(3)	2.3602(8)	2.6036(6)	2.159(2)	2.3607(8)	2.573(6)^*g*^
		2.168(3)	2.3677(9)	2.5919(6)	2.174(2)	2.3379(8)	2.577(1)^*g*^
C_NHC_–Si–Si	93.37(5)	98.8(2)	97.87(9)	97.04(7)			
		98.7(2)	96.74(9)	97.49(7)			
∑_Si_		308.8(3)^*f*^	303.9(1)^*f*^	304.4(1)^*f*^	290.7	292.7(1)^*f*^	297(1)^*g*^
		307.4(3)^*f*^	302.3(1)^*f*^	304.0(1)^*f*^			
DP		57	62	62	78	75	70
		58	64	63			
*δ*(^29^Si)^*e*^	224.5	38.4	34.9	18.7	19.06	10.9	–9.7
*δ*(C_NHC_)^*e*^	196.3	180.0	177.1	174.4	168.5	164.5	158.4

^*a*^The data were obtained from ref. 2*a*.

^*b*^The data were obtained from ref. 4*a*; the arithmetic mean value of the bonding parameters of two independent molecules in the unit cell of SiCl_2_(Idipp) is reported.

^*c*^The data were obtained from ref. 4*b*.

^*d*^The data were obtained from ref. 4*d*; the arithmetic mean value of the bonding parameters of the three independent molecules in the unit cell of SiI_2_(Idipp) is given.

^*e*^NMR chemical shifts are given in ppm in C_6_D_6_ at 298 K.

^*f*^The uncertainty (*u*) of the sum of angles is given in parenthesis and was calculated from the individual uncertainties (*u*_i_) by error propagation using the formula *u* = (∑(*u*_i_)^2^)^1/2^.

^*g*^The standard deviation (*σ*) of the unweighted arithmetic mean values *x*_u_ is given in parenthesis and was calculated using the formula *σ*^2^ = ∑(*x*_i_ – *x*_u_)^2^/*n*^2^ – *n*, where *x*_i_ is the respective individual value and *n* is the total number of individual values.

In the ^29^Si{^1^H} NMR spectra in C_6_D_6_, the Si^I^ halides display a characteristic singlet signal (**2-Cl**: *δ* = 38.4 ppm, **2-Br**: *δ* = 34.9 ppm, **2-I**: *δ* = 18.7 ppm), which appears at a lower field than that of the corresponding Si(ii) halides SiX_2_(Idipp) (X = Cl: *δ* = 19.2 ppm,4*a* X = Br: *δ* = 10.9 ppm,4*b* X = I: *δ* = –9.7 ppm4*d*) (Table 1).† In both series of compounds, the ^29^Si NMR signals shift progressively to a higher field upon Cl → Br → I substitution, and the same trend is observed for the ^13^C NMR signals of the Si-bonded C_NHC_ atoms (Table 1). The ^1^H and ^13^C{^1^H} NMR spectra of **2-Br** and **2-I** display a single set of signals for the homotopic Idipp groups originating from the time-averaged *C*_2_-symmetry of the *RR*/*SS* stereoisomers (the *C*_2_ axis perpendicularly intersects the Si–Si bond).† The peripheral N-bonded dipp substituents are locked in an orthogonal conformation *versus* the N-heterocyclic rings (Fig. 1), which, in combination with the presence of stereogenic Si centers, gives rise to two different sets of ^1^H/^13^C NMR signals for the *ortho*- and *meta*-positioned groups, respectively (see Fig. S1, S2, S6 and S8 in the ESI†). Whereas all the ^1^H (300.1 MHz) and ^13^C (75.47 MHz) NMR signals of **2-Br** were sharp at 298 K, several signals of **2-I** were broadened under the same conditions, suggesting a dynamic behavior (see Fig. S6–S8 in the ESI†). Variable-temperature ^1^H NMR spectroscopy of **2-Br** and **2-I** in the temperature range 203–333 K revealed a hindered rotation of the NHC groups about the Si–C_NHC_ bonds, which leads to a duplication of the signals of the dipp substituents and the N-heterocyclic C^4,5^-*H* ring protons in the slow-exchange limit spectra (see Fig. S4 and S10 in the ESI†). Analysis of the full coalescence behavior of the two singlet signals observed for the N-heterocyclic C^4,5^-*H* ring protons in the temperature range 203–333 K (see Fig. S4 and S10 in the ESI†) allowed an estimation of the standard Gibbs energy of activation for the hindered Si–C_NHC_ rotation (**2-Br**: Δ*G*^≠^ = 46 kJ mol^–1^, *T*_c_ (coalescence temperature) = 228 K; **2-I**: Δ*G*^≠^ = 51 kJ mol^–1^, *T*_c_ = 248 K).†

**Scheme 2:**
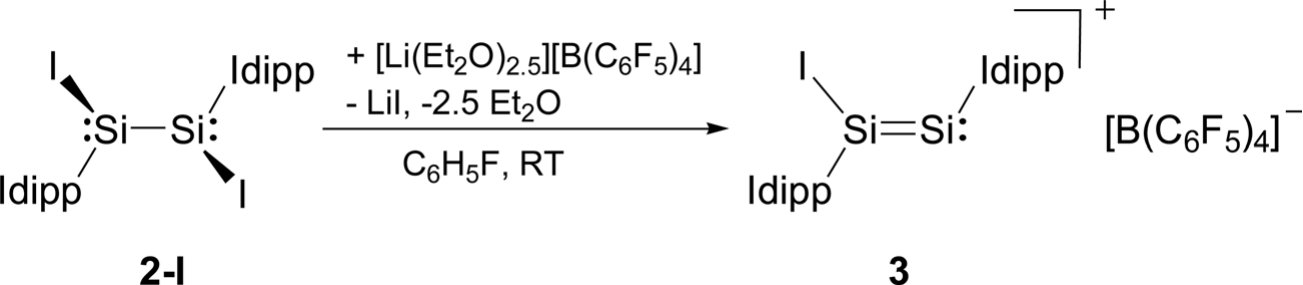
Synthesis of the disilicon(i) iodido salt **3** by iodide abstraction from **2-I**. Formal charges are not included for simplicity.

Compounds **2-X** contain many reactive sites for further functionalisation with the most appealing ones being the displacable halide and Idipp groups, which are not available in the silicon(i) congeners **C** and **D** (Chart 1). First reactivity studies were carried out focusing on the abstraction of the halide groups. In fact, the addition of one equivalent of [Li(Et_2_O)_2.5_][B(C_6_F_5_)_4_] to a solution of **2-I** in fluorobenzene at ambient temperature was accompanied by a colour change from bright to dark red and precipitation of LiI. Iodide abstraction from **2-I** selectively afforded the disilicon(i) salt [Si_2_(I)(Idipp)_2_][B(C_6_F_5_)_4_] (**3**), as evidenced by NMR spectroscopy of the crude reaction mixture (Scheme 2). The salt was isolated after a work-up and crystallisation from a fluorobenzene/*n*-hexane mixture as dark red crystals of the fluorobenzene monosolvate (**3**·(C_6_H_5_F)) in 62% yield, and was comprehensively characterised.†

Compound **3**·(C_6_H_5_F) is an extremely air-sensitive solid, which is instantly degraded by air to a colourless powder. It is stable in THF-*d*_8_ solution for several days under strict exclusion of air, and decomposes upon heating in a sealed glass capillary tube under vacuum at 208 °C.

**Fig. 2:**
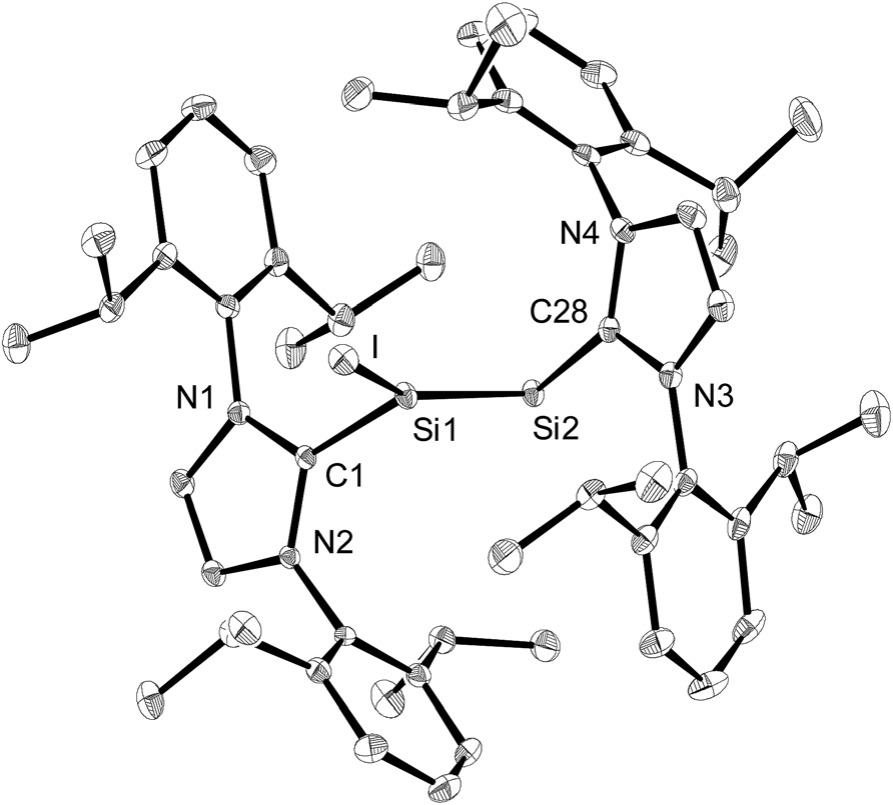
Diamond plot of the molecular structure of the cation of **3**·(C_6_H_5_F) in the single crystal. Thermal ellipsoids represent 30% of the electronic probability at 123(2) K. Hydrogen atoms are omitted for clarity. Selected bond lengths [Å], bond angles [°] and torsion angles [°]: C1–Si1 1.901(2), Si1–I 2.4654(7), Si1–Si2 2.1739(9), Si2–C28 1.931(2); C1–Si1–I 104.56(7), C1–Si1–Si2 112.83(7), I–Si1–Si2 142.27(3), Si1–Si2–C28 96.61(7); C1–Si1–Si2–C28 –178.5(1), I–Si1–Si2–C28 –6.71(9).

The solid-state structure of **3**·(C_6_H_5_F) was determined by single-crystal X-ray diffraction and it is composed of well separated [Si_2_(I)(Idipp)_2_]^+^ and [B(C_6_F_5_)_4_]^–^ ions.† The closest Si···F interionic contacts (6.371(3) Å) are significantly longer than the sum of the van der Waals radii of silicon and fluorine (3.6 Å).^23^ The cations [Si_2_(I)(Idipp)_2_]^+^ feature a trigonal planar coordinated Si1 atom (sum of the bond angles at Si1 = 359.7(1)°) and a two-coordinate Si2 atom with V-shaped geometry (Fig. 2). The two silicon atoms form a planar core with the end-on bonded iodine atom and the two C_Idipp_ atoms (C1 and C28). The Si–Si bond of **3**·(C_6_H_5_F) is considerably shorter (2.1739(9) Å) than the Si–Si single bond of **2-I** (2.3909(9) Å), and also shorter than the Si–Si double bond of **1** (2.229(1) Å),2*a* but lies in the reported range of Si=Si bond lengths.^24^ The presence of a Si–Si double bond was further confirmed by the electronic structure analysis of [Si_2_(I)(Idipp)_2_]^+^ (*vide infra*). The bulky NHC groups are *trans*-arranged at the Si–Si double bond (torsion angles: C1–Si–Si2–C28 = –178.5(1)° and I–Si1–Si2–C28 = –6.71(9)°) and orthogonally oriented with respect to the planar core of the cation.^25^ The angle at the two-coordinate Si atom is quite narrow (Si1–Si2–C28 = 96.61(7)°) and compares well with those observed in **1** (Si–Si–C_NHC_ = 93.37(5)°),2*a* the NHC-stabilised phosphasilenylidene (Idipp)Si=PR (R = C_6_H_2_-2,4,6-*t*Bu_3_; P–Si–C_NHC_ = 96.90(6)°)^9^ and the NHC-stabilised disilavinylidene (SIdipp)Si=Si(Br)R (R = C_6_H_2_-2,6-{CH(SiMe_3_)_2_}_2_-4-*t*Bu; Si–Si–C_NHC_ = 97.6(1)°).^10^ A rationale for the narrow angle at Si2 is provided by the NBO analysis of [Si_2_(I)(Idipp)_2_]^+^, which indicates the presence of a stereochemically active lone-pair in an orbital of high s-character (77%) and Si2 hybrid orbitals of high p-character employed for the σ-bonding to the Si1 atom and the NHC group (87 and 89%, respectively, see Table 4). The Si1–I bond (2.4654(7) Å) compares well with that of the iodotriaryldisilene Tip(I)Si=SiTip_2_ (2.4520(7) Å, Tip = C_6_H_2_-2,4,6-*i*Pr_3_),^26^ but is considerably shorter than the Si–I bond lengths of **2-I** (Si1–I1: 2.6036(6) Å; Si2–I2 2.5919(6) Å) (Table 1). This trend can be rationalised according to comparative NBO analyses of **3** and **2-I** with the increased s-character of the Si hybrid orbital employed in the Si–I bond of **3** (20%) compared to that in **2-I** (4%), and this is also reflected in the Si–I Wiberg bond indexes (**3**: WBI (Si–I) = 0.89; **2-I**: WBI (Si–I) = 0.78) (see Table 4 and Table S8 in the ESI†). The Si–C_NHC_ bond lengths of **3**·(C_6_H_5_F) (1.901(2) and 1.931(2) Å) have similar values to those of **2-I** (1.943(2) Å and 1.939(2) Å) and **1** (1.927(2) Å) (Table 1).2*a*

**Fig. 3:**
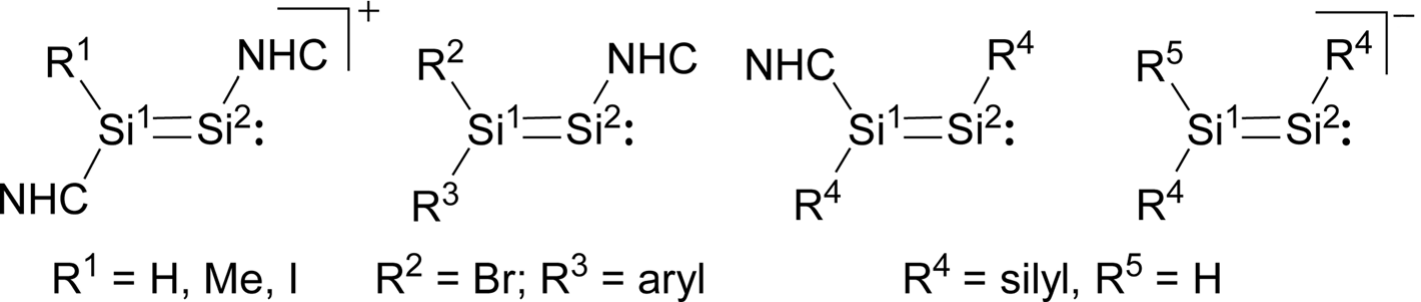
Structural formulae of the cations [(NHC)(R^1^)Si=Si(NHC)]^+^ (NHC = C[N(dipp)CH]_2_ (Idipp)), the NHC-stabilised disilavinylidenes R^2^R^3^Si=Si(NHC) (NHC = C[N(dipp)CH_2_]_2_ (SIdipp), R^3^ = C_6_H_2_-2,6-{CH(SiMe_3_)_2_}_2_-4-*t*Bu), the NHC-stabilised disilynes (NHC)R^4^Si=SiR^4^ (NHC = C[N(Me)CMe]_2_, R^4^ = Si*i*Pr{CH(SiMe_3_)_2_}_2_) and the disilenide anions [R^4^R^5^Si=SiR^4^]^–^. Formal charges are not included for simplicity.

Notably, a comparison of [Si_2_(I)(Idipp)_2_]^+^ with the related cations [Si_2_(H)(Idipp)_2_]^+^ and [Si_2_(Me)(Idipp)_2_]^+^, the NHC-stabilised disilavinylidenes, the NHC-stabilised disilynes and the disilenide anions (Fig. 3) reveals a similar electronic structure of these molecules leading to common structural features, such as a planar core, similar Si=Si bond lengths and similar bond angles at the two-coordinate Si atom (Table 2).^10^,^27^–^29^

**Table 2:** Comparison of selected bonding parameters of the cations [(NHC)(R^1^)Si=Si(NHC)]^+^ (NHC = C[N(dipp)CH]_2_ (Idipp), R^1^ = I, H, Me), the neutral NHC-stabilised disilavinylidenes R^2^R^3^Si=Si(NHC) (NHC = C[N(dipp)CH_2_]_2_ (SIdipp); R^2^ = Br, R^3^ = C_6_H_2_-2,6-{CH(SiMe_3_)_2_}_2_-4-*t*Bu (Tbb)), the NHC-stabilised disilynes (NHC)R^4^Si=SiR^4^ (NHC = C[N(Me)CMe]_2_ (IMe_4_), R^4^ = Si*i*Pr{CH(SiMe_3_)_2_}_2_) and the disilenide anions [R^4^HSi=SiR^4^]^–^

	*d*(Si–Si) [Å]	∠Si^1^–Si^2^–R [°]	Ref.
[(Idipp)(I)Si^1^=Si^2^(Idipp)]^+^	2.1739(9)	96.61(7)	This paper
[(Idipp)(H)Si^1^=Si^2^(Idipp)]^+^	2.1873(8)	95.34(6)	27
[(Idipp)(Me)Si^1^=Si^2^(Idipp)]^+^	2.1909(8)	95.13(6)	27
Tbb(Br)Si^1^ = Si^2^(SIdipp)	2.167(2)	97.6(1)	10
(IMe_4_)R^4^Si^1^=Si^2^R^4^	2.1989(6)	120.35(2)	28
[R^4^(H)Si^1^=Si^2^R^4^]^–^	2.2034(9)	102.69(3)	29

**Scheme 3:**
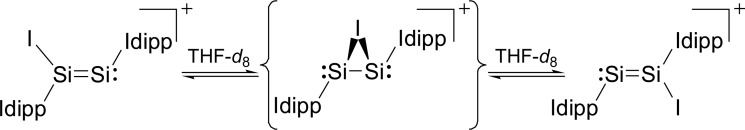
Topomerisation of [Si_2_(I)(Idipp)_2_]^+^ occurring in solution. The tentative intermediate, a NHC-stabilised disilaiodonium ion, is depicted in curly brackets. Formal charges are omitted for simplicity.

Variable-temperature ^1^H, ^29^Si and ^13^C NMR studies of **3**·(C_6_H_5_F) in THF-*d*_8_ revealed an interesting dynamic process leading to an exchange of the heterotopic Si sites. The degenerate isomerisation (topomerisation)^30^ is suggested by quantum chemical calculations to proceed *via* a NHC-stabilised disilaiodonium ion (Scheme 3).^31^

**Fig. 4:**
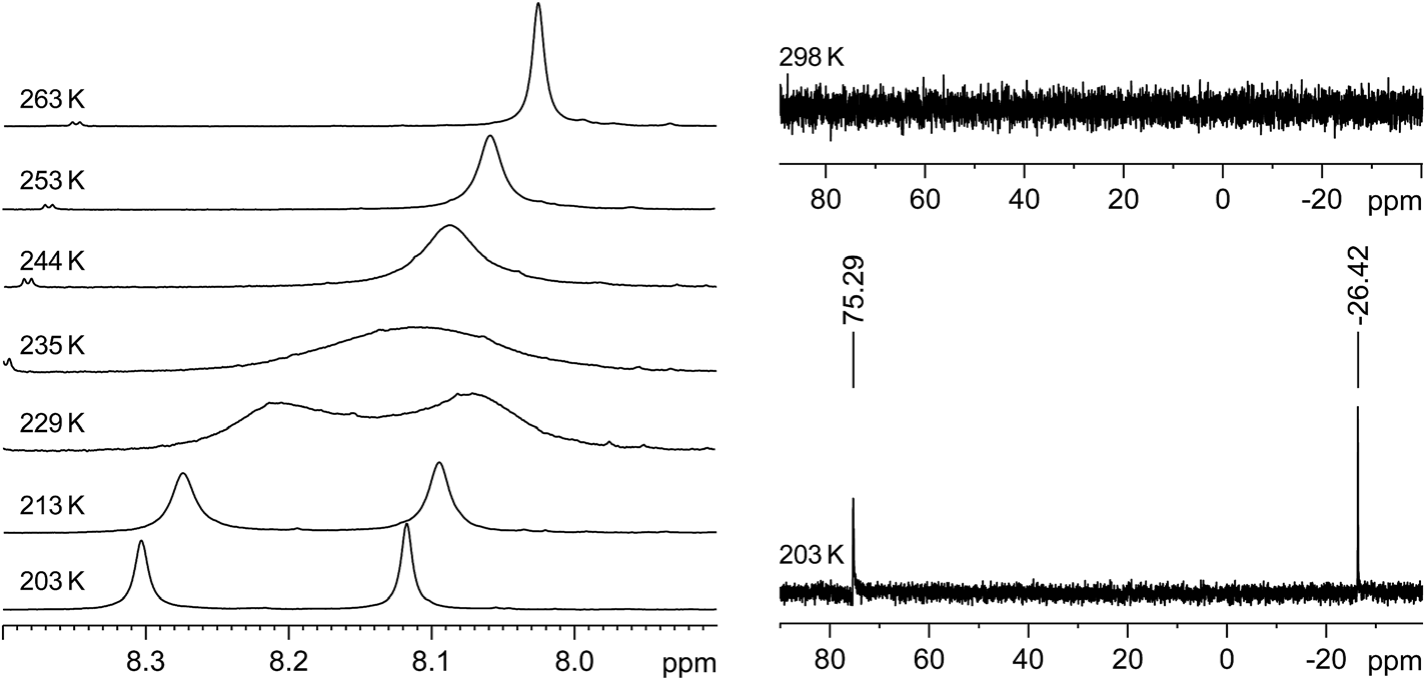
Left: Excerpts of the variable-temperature ^1^H NMR (300.1 MHz) spectra of **3**·(C_6_H_5_F) in THF-*d*_8_ in the temperature range 203–263 K showing the coalescence of the C^4,5^-*H* signals. Right: ^29^Si{^1^H} NMR (59.63 MHz) spectra of **3**·(C_6_H_5_F) in THF-*d*_8_ at 203 K (bottom) and 298 K (top).

Thus, two well separated ^29^Si NMR signals at *δ* = –26.4 ppm and +75.3 ppm are observed in the slow exchange limit ^29^Si{^1^H} NMR spectrum of **3**·(C_6_H_5_F) at 203 K (Fig. 4, right), which are assigned by B97-D3/IGLOIII/ZORA-def2-TZVP (iodine atom)/ZORA/COSMO(THF) calculations to the three-coordinate, I-bonded silicon (Si1) and the two-coordinate silicon (Si2) nuclei, respectively.^32^ In comparison, no ^29^Si NMR signals could be detected at 298 K even after a long accumulation time (intermediate time regime) (Fig. 4, right). Similarly, the slow-exchange limit ^1^H NMR spectrum of **3**·(C_6_H_5_F) at 203 K displays a double set of resonance signals for the chemically different Idipp groups (see Fig. S11 and S12 in the ESI†). Most distinctive are the two singlet signals for the N-heterocyclic C^4,5^-*H* ring protons, which, upon increasing temperature coalesce at *T*_c_ = 235 K, and then merge into one sharp signal in the fast-exchange limit ^1^H NMR spectrum at 298 K (see Fig. 4, left and Fig. S20 in the ESI†). Likewise, the ^13^C{^1^H} NMR spectrum of **3**·(C_6_H_5_F) at 203 K shows a double set of signals for the inequivalent Idipp groups (for example, two singlets for the Si-bonded C_NHC_ nuclei at *δ* = 153.6 and 172.2 ppm), which merge into one set of signals at 298 K (see Fig. S14–S17 in the ESI†). The number and relative intensity of the signals in the slow-exchange limit ^1^H and ^13^C{^1^H} NMR spectra of **3**·(C_6_H_5_F) are compatible with the results of the single-crystal X-ray diffraction and show an averaged *C*_s_-symmetric structure of the cation [Si_2_(I)(Idipp)_2_]^+^ with fast rotating NHC substituents about the respective Si–C_NHC_ bonds.^33^

The rate constants of the dynamic process were determined by full line-shape analyses of the signals of the N-heterocyclic C^4,5^-*H* ring protons in the temperature range of 203–263 K (Fig. 4, left and Fig. S20 in the ESI†). An Eyring plot of ln(*k*/*T*) against 1/*T* (*k* = rate constant, *T* = temperature) afforded a linear relationship (see Section 3 in the ESI†). The activation parameters of the dynamic process were obtained from the slope and the intercept of the corresponding regression line (*R*^2^ = 0.9966) and were found to be Δ*H*^≠^ = 47.3 (±0.7) kJ mol^–1^, Δ*S*^≠^ = 1.39 (±3.0) J K^–1^ mol^–1^ and Δ*G*^≠^ (*T*_c_ = 235 K) = 47.0 (±1.4) kJ mol^–1^.†

The potential energy hypersurface (PES) of the cation [Si_2_(I)(Idipp)_2_]^+^ was studied by quantum chemical calculations at the B97-D3/**I** level of theory^17^ in order to analyse the topomerisation process of **3**·(C_6_H_5_F) observed in solution. Geometry optimization of [Si_2_(I)(Idipp)_2_]^+^ afforded a “σ-bonded” minimum structure (**3_calc_**) with an excellent agreement between the calculated and the experimental bond lengths obtained for **3**·(C_6_H_5_F) by single-crystal X-ray crystallography (Fig. 2 and Table 3).

**Table 3:** Comparison of selected experimental bond lengths, bond angles and dihedral angles of **3**·(C_6_H_5_F) with the calculated (B97-D3/**I**)^17^ bond lengths and angles of **3_calc_**, **3^TS^_calc_** and **3′_calc_**. Atom numbering of the experimental structure (Fig. 2) was taken over to the calculated structure

	Si1–Si2 [Å]	Si1–C1 [Å]	Si2–C28 [Å]	Si–I [Å]	C1–Si1–Si2 [°]	C1–Si1–I [°]	Si1–Si2–C28 [°]	I–Si1–Si2 [°]	*φ*_NHC1_ ^*a*^ [°]	*φ*_NHC2_ ^*a*^ [°]
**3**·(C_6_H_5_F)_exp_	2.1739(9)	1.901(2)	1.931(2)	2.4654(7)	112.83(7)	104.56(7)	96.61(7)	142.27(3)	96.69(7)	95.78(7)
**3_calc_**	2.171	1.903	1.923	2.502	112.06	103.58	96.96	144.35	89.10	89.68
**3^TS^_calc_**	2.366	1.950	1.936	2.618	95.39	104.66^*b*^	97.44	87.14^*b*^	91.98	45.56
				3.440						
**3′_calc_**	2.463	1.977	1.975	2.696	101.49	98.24^*b*^	101.79	62.84^*b*^	78.45	81.02

^*a*^The dihedral angles *φ*_NHC1_ and *φ*_NHC2_ are the respective angles between the least-square plane of the atoms C1, Si1, Si2 and C28 and the respective NHC central ring planes.

^*b*^The corresponding angles C28–Si2–I and I–Si2–Si1 are 76.12° and 49.48° (**3^TS^_calc_**) and 98.24° and 62.84° (**3′_calc_**).

**Fig. 5:**
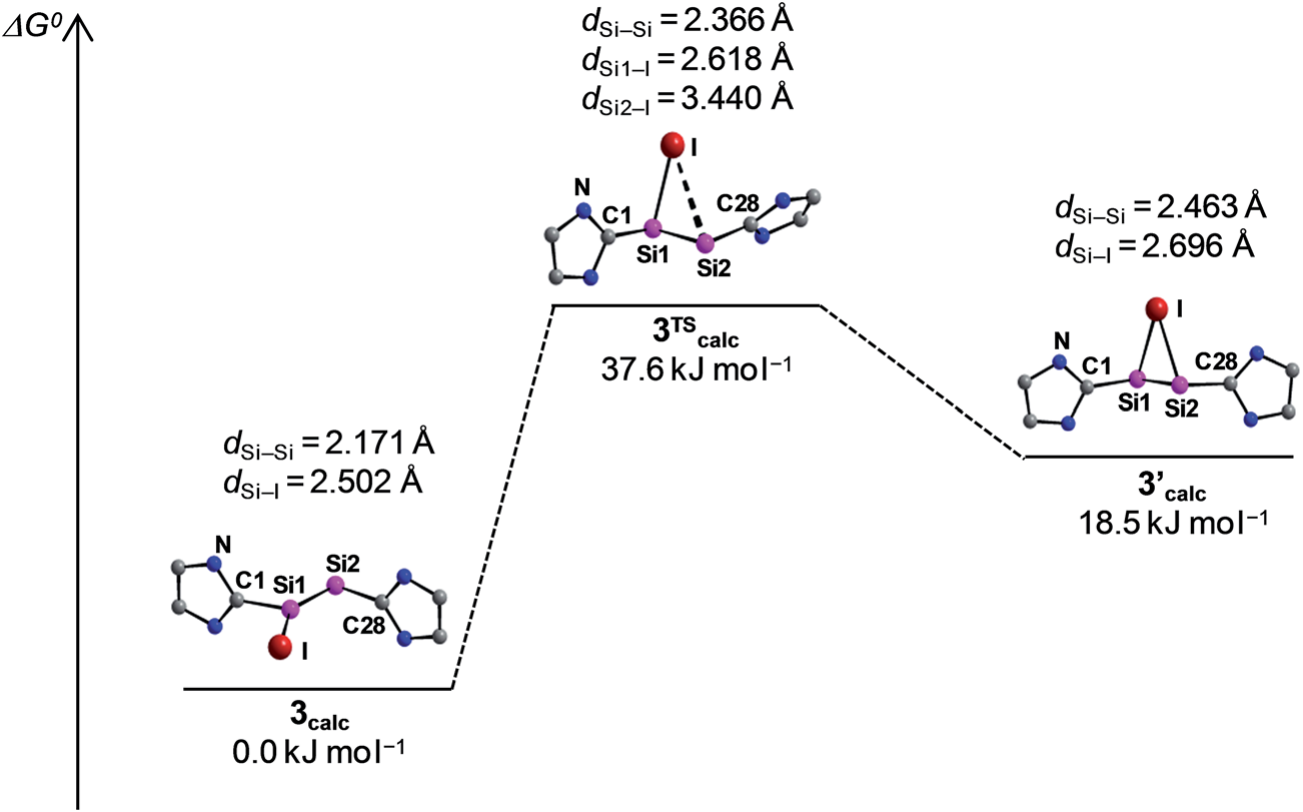
Schematic Gibbs energy profile (*T* = 298 K) for the degenerate isomerisation of the cation [Si_2_(I)(Idipp)_2_]^+^, including the optimized minimum structures **3_calc_** and **3′_calc_** and the transition state **3^TS^_calc_** with selected bonding parameters. The N-bonded dipp substituents are omitted for clarity and only one half of the symmetric energy profile is depicted.

Furthermore, a “π-bonded” *C*_2_-symmetric minimum structure (**3′_calc_**) was located on the PES, which is less stable by 18.5 kJ mol^–1^ than the “σ-bonded” isomer of [Si_2_(I)(Idipp)_2_]^+^ (**3_calc_**) (Fig. 5). The two minimum structures are connected *via* a transition state (**3^TS^_calc_**), which lies at an energy 37.6 kJ mol^–1^ higher than the overall minimum structure **3_calc_** (Fig. 5). The transition state has an imaginary frequency of –92 cm^–1^, and connects the two minimum structures *via* a rocking vibrational mode of the iodine atom. The calculated barrier of 37.6 kJ mol^–1^ compares acceptably well with that obtained from the variable-temperature NMR studies (*vide supra*). The most striking bonding parameters of **3′_calc_** are the elongated Si–Si single bond (2.463 Å), which is considerably longer than the Si=Si bond of **3_calc_** (2.171 Å), as well as the Si–I bonds (2.696 Å), which are longer than that of **3_calc_** (Si–I: 2.502 Å). These bonding parameters suggest that **3′_calc_** can be better described as a NHC-stabilised disilaiodonium ion^34^ rather than a Si_2_(Idipp)_2_ (**1**) π-complex of I^+^. Notably, the structure of **3′_calc_** is reminiscent of those of the symmetrical 1,2-bridged halonium ions, which have been extensively studied in organic chemistry.^35^

**Fig. 6:**
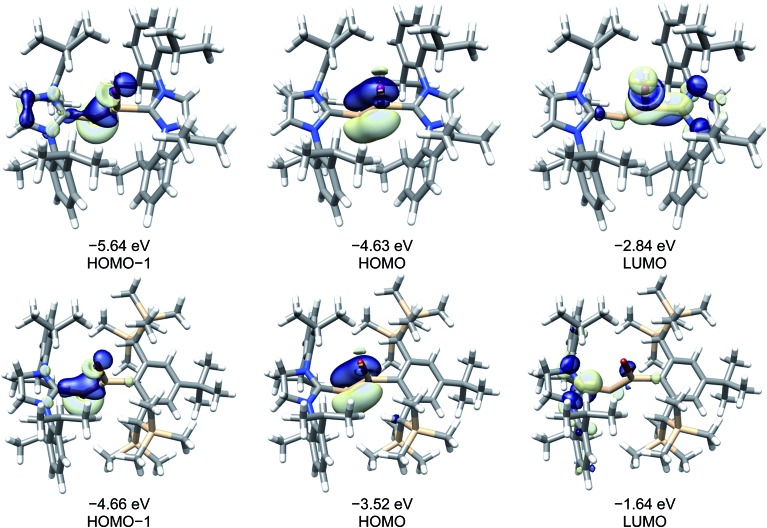
Selected Kohn–Sham orbitals (B97-D3/**I**) of **3_calc_** (top) and (*Z*)-(SIdipp)Si=Si(Br)R (bottom, R = C_6_H_2_-2,6-{CH(SiMe_3_)_2_}_2_-4-*t*Bu) and their corresponding energy eigenvalues. Isosurface value: 0.05 e bohr^–3^. The LUMO of **3_calc_** is a symmetrical combination of a π* NHC and σ* Si–I orbital, whereas the LUMO of (*Z*)-(SIdipp)Si=Si(Br)R is a π* NHC orbital.

A comparison of the frontier Kohn–Sham orbitals of the ion [Si_2_(I)(Idipp)_2_]^+^ with those of the NHC-stabilised disilavinylidene (SIdipp)Si=Si(Br)R (R = C_6_H_2_-2,6-{CH(SiMe_3_)_2_}_2_-4-*t*Bu)^10^ or the model compound (IMe_4_)RSi=SiR (IMe_4_ = C[N(Me)CMe]_2_, R = SiMe_3_)^28^ revealed the same symmetry properties, shape and approximate energy of the frontier orbitals, confirming the isolobal analogy of these molecules, which can be traced back to the electronic relationship of the fragments Si(NHC) and (SiR)^–^ (R = singly bonded substituent). In all cases, the HOMO is the Si=Si π-bonding orbital, which is followed by the lone-pair orbital at the two-coordinate Si atom (HOMO–1) (Fig. 6). The electronic structure of the “σ-bonded” isomer of [Si_2_(I)(Idipp)_2_]^+^ (**3_calc_**) was analysed by the natural bond orbital (NBO) method and natural resonance theory (NRT) (see Table 4), and the results were compared with those of the “π-bonded” isomer of [Si_2_(I)(Idipp)_2_]^+^ (**3′_calc_**) (see Table S10 in the ESI†) and **2-I_calc_** (see Table S8 in the ESI†). NBO analysis of the wavefunction of **3_calc_** suggests a high localization of the orbitals describing the Si=Si, Si–C_NHC_, and Si–I bonds (Table 4). For example, the Si–Si σ-bond NBO is occupied by 1.90 electrons and the Si–Si π-bond NBO is occupied by 1.89 electrons. Whereas the Si–Si σ-bond is slightly polarised towards the Si1 atom and is formed from the overlap of a Si1 natural hybrid orbital (NHO) with high s-character (59%) and a Si2 NHO with high p-character (87%), the Si–Si π-bond NBO is less polarised and is formed from pure Si p-orbitals. The moderate polarisation of the Si=Si bond of **3_calc_** and the high occupancies of its NBO lead to a high Wiberg bond index (WBI) of 1.81, which is twice as large as the WBI of the Si–Si single bond of **3′_calc_** (0.89) and **2-I** (0.96). These findings verify the presence of a Si=Si bond in the “σ-bonded” isomer (**3_calc_**) and a Si–Si single bond in the “π-bonded” isomer (**3′_calc_**) of [Si_2_(I)(Idipp)_2_]^+^ or in **2-I**, and are further confirmed by the NRT Si–Si bond orders, which, in the case of **3_calc_**, is twice as large (NRT-BO = 1.95, Table 4) as that of **3′_calc_** (0.95) or **2-I** (0.93) (see Tables S8 and S10 in the ESI†). In the “σ-bonded” isomer **3_calc_**, the two-coordinate silicon atom (Si2) carries a lone pair of electrons in an NHO with high s-character (77%, Table 4), as was found for the NHC-stabilised disilavinylidene.^10^ In comparison, both Si atoms in the “π-bonded” isomer **3′_calc_** carry a lone pair of electrons in NHO orbitals with high s-character (79%, see Table S10 in the ESI†), providing additional evidence for the different structures of the “σ-bonded” and “π-bonded” isomers of [Si_2_(I)(Idipp)_2_]^+^.

**Table 4:** Selected results of the natural bond orbital (NBO) and natural resonance theory (NRT) analyses of **3_calc_** (B97-D3/**I**). Atom numbering of the experimental structure (Fig. 2) was taken over to the calculated structure **3_calc_**

NBO analysis					NPA partial charges^*b*^		NRT analysis^*c*^	
	occ.^*a*^	pol.^*a*^ [%]	hyb.^*a*^	WBI^*a*^			tot/cov/ionic^*a*^	
σ(Si1–Si2)	1.90	62.0 (Si1)	sp^0.69^ (Si1)	1.81	Si1	0.30	Si1–Si2	1.95/1.55/0.41
		38.1 (Si2)	sp^7.03^ (Si2)					
π(Si1–Si2)	1.89	58.1 (Si1)	p (Si1)		Si2	0.18		
		41.9 (Si2)	p (Si2)					
σ(Si1–C1)	1.95	24.3 (Si1)	sp^3.85^ (Si1)	0.72	C1	0.05	Si1–C1	1.00/0.47/0.53
		75.8 (C1)	sp^1.39^ (C1)		∑(NHC1)	0.41		
σ(Si2–C28)	1.93	21.8 (Si2)	sp^8.50^ (Si2)	0.76	C28	0.06	Si2–C28	1.03/0.43/0.61
		78.3 (C28)	sp^1.28^ (C28)		∑(NHC2)	0.28		
σ(Si1–I)	1.96	34.5 (Si1)	sp^3.87^ (Si1)	0.89	I	–0.18	Si1–I	0.93/0.64/0.30
		65.5 (I)	sp^5.62^ (I)					
n(Si2)	1.77		sp^0.29^		∑(Si_2_I)	0.30		

^*a*^occ.: occupancy, pol.: polarization, hyb.: hybridization, WBI: Wiberg bond index, tot/cov/ionic: total bond order/covalent bond order/ionic bond order.

^*b*^Partial charges obtained by natural population analysis (NPA).

^*c*^A local NRT analysis was carried out including the Si1, Si2, I, N, C1 and C28 atoms.

Finally, a natural population analysis of **3_calc_** indicates a considerable charge flow from the NHC to the disilaiodonium ion [Si_2_I]^+^, as evidenced by the overall NPA charges of the NHCs (Si1-bonded: *q*(∑(NHC)) = 0.41; Si2-bonded: *q*(∑(NHC)) = 0.28) (Table 4).

## Conclusions

An efficient method for the synthesis of silicon(i) halides Si_2_X_2_(Idipp)_2_ (**2-X**, X = Cl, Br, I) was developed, which involved a diastereoselective halogenation of Si_2_(Idipp)_2_ (**1**) with 1,2-dihaloethanes. This allowed the isolation of the first silicon(i) bromide (**2-Br**) and silicon(i) iodide (**2-I**) in high yield, enabling first reactivity studies of **2-I**. The geometric and electronic structures of **2-Br** and **2-I** were comprehensively studied by experimental and theoretical methods. Iodide abstraction from **2-I** selectively afforded the unprecedented disilicon(i) iodido salt [Si_2_(I)(Idipp)_2_][B(C_6_F_5_)_4_] (**3**), the geometric and electronic structure of which is isolobal to that of a NHC-stabilised disilavinylidene recently reported by our group. The topomerisation of the cation [Si_2_(I)(Idipp)_2_]^+^, leading to an exchange of the two heterotopic Si sites, was studied by variable-temperature NMR spectroscopy and the underlying dynamic process was analysed by quantum chemical calculations. The calculations suggest the intermediate formation of a *C*_2_-symmetric π-bonded isomer with homotopic Si sites reminiscent of the symmetrical 1,2-bridged halonium ions in organic chemistry. The present results corroborate the ability of N-heterocyclic carbenes to stabilise low-valent main-group element centers with unusual bonding features. Further studies addressing the reactivity of the NHC-stabilised Si(i) halides **2-X** and the Si^I^ salt **3** are currently underway.

## Supplementary Material

Supplementary information

Crystal structure data
