# Supplementary material for: Silicon(i) chemistry: the NHC-stabilised silicon(i) halides Si2X2(Idipp)2 (X = Br, I) and the disilicon(i)-iodido cation [Si2(I)(Idipp)2]+
Source: Chem Sci. 2015 Aug 17;6(11):6515–24. doi: 10.1039/c5sc02681d (PMC6054044; doi:10.1039/c5sc02681d)
Supplement: Supplementary file 1 [file SC-006-C5SC02681D-s001.pdf]

Electronic Supplementary Material (ESI) for Chemical Science.
This journal is © The Royal Society of Chemistry 2015

Supporting Information

1.	Experimental Section – General Part	S2
2.	Syntheses and analytical data of the compounds	S3
2.1	$Si_2Cl_2(Idipp)_2$ (**2-Cl**)	S3
2.2	$Si_2Br_2(Idipp)_2$ (**2-Br**)	S4
2.3	Thermolysis of **2-Br**	S8
2.4	$Si_2I_2(Idipp)_2\cdot 0.5(n\text{-}C_6H_{14})$ (**2-I**·0.5(n-C_6H_{14}))	S8
2.5	$[Si_2(I)(Idipp)_2][B(C_6F_5)_4]\cdot(C_6H_5F)$ (**3**·(C_6H_5F))	S13
3.	Determination of the standard Gibbs energy of activation for **3**·(C_6H_5F)	S20
4.	Crystal structure determination of **2-Br**·0.5(n-C_6H_{14}), **2-I**·0.5(n-C_6H_{14}) and **3**·(C_6H_5F)	S23
4.1	Correlation of the Si–Si bond length in base-stabilized Si(I) compounds to the sum of bond angles at the silicon atoms	S26
5.	Electronic structure calculations	S28
5.1	Comparison of selected experimental and calculated bonding parameters of **2-Br**, (S,S)-**2-Br$_{calc}$** and (R,S)-**2-Br$_{calc}$**	S29
5.2	Comparison of selected experimental and calculated bonding parameters of **2-I**, **2-I$_{calc}$**, **3**, **3$_{calc}$**, **3$^{TS}_{calc}$** and **3'$_{calc}$**	S30
5.3	Results of the natural bond orbital (NBO) and natural resonance theory (NRT) analyses of $[SiBr_2(IDipp)]_{calc}$, (S,S)-**2-Br$_{calc}$**, **2-I$_{calc}$**, **3$_{calc}$** and **3'$_{calc}$**	S31
5.4	Cartesian coordinates [Å] and SCF energies of the calculated structures of $[SiBr_2(Idipp)]_{calc}$, (S,S)-**2-Br$_{calc}$**, (R,S)-**2-Br$_{calc}$**, **2-I$_{calc}$**, **3$_{calc}$**, **3$^{TS}_{calc}$** and **3'$_{calc}$**	S34

1. Experimental Section – General Part

All experiments were carried out under strict exclusion of water and oxygen in an atmosphere of argon using Schlenk or glove box techniques. The commercially received argon had a purity of ≥99.999 % and was further passed through an argon purification system to remove traces of O_2 and H_2O. The glassware was dried in an oven at approximately 110 °C and baked under vacuum prior to use. Each solvent was refluxed over an appropriate drying agent (*n*-hexane: sodium wire / benzophenone / tetraglyme (0.5 vol%); diethyl ether: sodium wire / benzophenone, THF: sodium wire / benzophenone, toluene: sodium wire), purged several times during reflux with argon and distilled under argon. Fluorobenzene was stirred over CaH_2 and trap-to-trap condensed. All solvents were degassed by three freeze-pump-thaw cycles and stored in the glove box.
The C, H, N elemental analyses were carried out in triplicate for each sample on an Elementar Vario Micro elemental analyser. The C, H, N values did not differ by more than ±0.3 %. The mean C, H, N values are given below for each compound. The melting points were determined in triplicate for each sample using a Büchi melting point B-545 apparatus. The samples were sealed in capillary tubes under vacuum and heated once with a gradient of 5 K min^{-1} for a rough determination of the melting point or temperature of starting decomposition. Heating of the second and third sample was then repeated with a gradient of 2 K min^{-1}, starting 20 K below the temperature of melting or decomposition determined in the first experiment. The decomposition of the compounds was verified by optical inspection.
All NMR spectra were recorded on a Bruker Avance DMX-300 or DPX-300 NMR spectrometer in dry deoxygenated benzene-d_6, THF-d_8 or toluene-d_8. The deuterated solvents were stirred over sodium powder and then trap-to-trap condensed and stored over 4 Å molecular sieves. The ^{1}H and ^{13}C{^{1}H} NMR spectra were calibrated against the residual proton and natural abundance ^{13}C resonances of the deuterated solvent relative to tetramethylsilane (benzene-d_6: δ_H = 7.15 ppm, δ_C = 128.0 ppm; THF-d_8: δ_H = 1.73 ppm, δ_C = 25.3 ppm; toluene-d_8: δ_H = 2.09 ppm, δ_C = 20.4 ppm). The ^{29}Si{^{1}H}, ^{19}F{^{1}H} and ^{11}B{^{1}H} NMR spectra were calibrated against external pure $SiMe_4$, $CFCl_3$ and $BF_3{\cdot}Et_2O$, respectively. The NMR standards were filled in capillaries, which were sealed off and introduced into 5 mm NMR tubes containing the corresponding deuterated solvent (benzene-d_6, THF-d_8 and toluene-d_8). The NMR tubes were vacuum-sealed and used for the calibration. The following abbreviations were used for the multiplicities and forms of the NMR signals: s = singlet, d = doublet, dd = doublet of doublets; t = triplet, sept = septet, m = multiplet, dm = doublet of multiplets, br = broad. The full width at half maximum of broad signals was designated with $\Delta\nu_{½}$. The ^{1}H and ^{13}C NMR signals of all compounds were assigned by a combination of

HMQC, HMBC and DEPT experiments. This allowed an unequivocal assignment of all proton and carbon resonances including those of the diastereotopic methyl groups of the isopropyl substituents, which were labeled with the subscript letters A and B, respectively. The label A was used for the methyl groups with the lower ^{1}H NMR chemical shift. The Idipp substituents of compound **3** were designated with the letters X and Y, respectively. The label X was used for the Idipp substituent bonded to the three-coordinated Si atom bearing the iodine atom.

The compounds $Si_2(Idipp)_2$ (**1**)[S1] and $[Li(Et_2O)_{2.5}][B(C_6F_5)_4]$[S2] were prepared following the procedures described in the literature. 1,2-Dichloroethane, 1,2-dibromoethane and 1,2-diiodoethane were purchased from Sigma Aldrich. 1,2-Dichloroethane and 1,2-dibromoethane were stirred over predried K_2CO_3 for three days and obtained as colourless liquids after distillation under argon. 1,2-Diiodoethane was recrystallised from toluene at −30 °C and obtained as a colourless solid.

2. Syntheses and analytical data of the compounds

2.1 $Si_2Cl_2(Idipp)_2$ (2-Cl)

A suspension of **1** (350 mg, 0.42 mmol) in 25 mL of toluene was cooled to −30 °C and 0.65 mL (0.42 mmol) of a 0.650 M stock solution of 1,2-dichloroethane in toluene was added dropwise over a period of 10 minutes. During this time the solid dissolved, the colour of the solution changed from dark red to red-orange and evolution of ethene gas was observed. Static vacuum was applied and the reaction mixture was stirred for one hour at −30 °C and then warmed to ambient temperature and stirred for another hour at ambient temperature. The solvent was removed under vacuum and a red solid was obtained. The red solid was dissolved in 4 mL of THF and the red-orange solution was stored at −60 °C for three days. The red-orange, microcrystalline solid was collected by filtration at −60 °C and dried under vacuum for two hours at ambient temperature. The compound **2-Cl** was obtained as a red-orange, extremely air-sensitive, microcrystalline solid.

Crude yield: 190 mg (0.236 mmol, 49 %). The solid was found by ^{1}H NMR spectroscopy to contain 6 % Idipp. Recrystallisation of the solid from toluene or THF at low temperature afforded pure **2-Cl**.

^{1}H NMR (300.1 MHz, C_6D_6, 298 K, ppm): δ_H = 1.00, 1.05, 1.31, 1.65 (d each, ^{3}J(H,H) = 6.9 Hz, 12H each, 4 × C^2-CH(CH_3)$_A$(CH_3)$_B$ + 4 × C^6-CH(CH_3)$_A$(CH_3)$_B$ + 4 × C^2-CH(CH_3)$_A$(CH_3)$_B$ +

[S1] Y. Wang, Y. Xie, P. Wei, R. B. King, H. F. Schaefer III, P. v. R. Schleyer, G. H. Robinson, *Science* **2008**, *321*, 1069.

[S2] M. Lehmann, A. Schulz, A. Villinger, *Angew. Chem. Int. Ed.* **2009**, *48*, 7444; *Angew. Chem.* **2009**, *121*, 7580.

4 × C^6-CH(CH_3)$_A$(C*H*$_3$)$_B$), 3.02 – 3.16 (m, 8H, 4 × C^2-C*H*(CH_3)$_A$(CH_3)$_B$ + 4 × C^6-C*H*(CH_3)$_A$(CH_3)$_B$), 6.30 (s, 4H, 2 × $C^{4,5}$-*H*, NCH), 7.00, 7.11 (d each, ^3J(H,H) = 7.5 Hz, 4H each, 4 × C^3-*H* + 4 × C^5-*H*, C_6H_3), 7.21 (t, ^3J(H,H) = 7.5 Hz, 4H, 4 × C^4-*H*, C_6H_3).[S3]

2.2 Si_2Br_2(Idipp)$_2$ (2-Br)

A suspension of **1** (1.000 g, 1.20 mmol) in 60 mL of toluene was cooled to −45 °C and 1.3 mL (1.20 mmol) of a 0.938 M stock solution of 1,2-dibromoethane in toluene was added dropwise over a period of 10 minutes. Upon addition the solid dissolved, the colour of the solution changed from dark red to red-orange and evolution of ethene gas was observed. Static vacuum was applied and the reaction mixture was stirred for one hour at −45 °C, and then warmed to ambient temperature and stirred for another hour at ambient temperature. The solution was concentrated under vacuum to approximately 5 mL, whereupon a red solid started to precipitate. The suspension was stored at −60 °C for five days to complete crystallisation of **2-Br**. The red-orange, microcrystalline solid was collected by filtration at −60 °C and dried under vacuum for two hours at ambient temperature. Compound **2-Br** was obtained as a red-orange, extremely air-sensitive, microcrystalline solid. Yield: 1.175 g (1.18 mmol, 98 %).

Compound **2-Br** turns beige upon heating to 190 °C and decomposes to a dark brown liquid at 196 °C. Elemental analysis calcd (%) for $C_{54}H_{72}Br_2N_4Si_2$ (993.14): C 65.30, H 7.31, N 5.64; found: C 64.19, H 7.28, N 5.20 %.[S4]

^{1}H NMR (300.1 MHz, C_6D_6, 298 K, ppm, Figure S1): δ_H = 0.97 (d, ^3J(H,H) = 6.9 Hz, 12H, 4 × C^2-CH(C*H*$_3$)$_A$(CH_3)$_B$), 1.02 (d, ^3J(H,H) = 6.9 Hz, 12H, 4 × C^6-CH(C*H*$_3$)$_A$(CH_3)$_B$), 1.37 (d, ^3J(H,H) = 6.9 Hz, 12H, 4 × C^2-CH(CH_3)$_A$(C*H*$_3$)$_B$), 1.68 (d, ^3J(H,H) = 6.9 Hz, 12H, 4 × C^6-CH(CH_3)$_A$(C*H*$_3$)$_B$), 3.09 (sept, ^3J(H,H) = 6.9 Hz, 4H, 4 × C^2-C*H*(CH_3)$_A$(CH_3)$_B$), 3.16 (sept, ^3J(H,H) = 6.9 Hz, 4H, 4 × C^6-C*H*(CH_3)$_A$(CH_3)$_B$), 6.29 (s, 4H, 2 × $C^{4,5}$-*H*, NCH), 6.99 (dd, ^3J(H,H) = 7.7 Hz, ^4J(H,H) = 1.3 Hz, 4H, 4 × C^5-*H*, C_6H_3), 7.10 (dd, ^3J(H,H) = 7.7 Hz, ^4J(H,H) = 1.3 Hz, 4H, 4 × C^3-*H*, C_6H_3), 7.20 (t, ^3J(H,H) = 7.7 Hz, 4H, 4 × C^4-*H*, C_6H_3).

^{13}C{^{1}H} NMR (75.47 MHz, C_6D_6, 298 K, ppm, Figure S2): δ_C = 22.6 (s, 4C, 4 × C^6-CH(CH_3)$_A$(*C*H$_3$)$_B$), 23.5 (s, 4C, 4 × C^2-CH(CH_3)$_A$(*C*H$_3$)$_B$), 25.9 (s, 4C, 4 × C^2-CH(*C*H$_3$)$_A$(CH_3)$_B$), 26.0 (s, 4C, 4 × C^6-CH(*C*H$_3$)$_A$(CH_3)$_B$), 28.8, 28.9 (s each, 4C each, 4 × C^2-*C*H(CH_3)$_A$(CH_3)$_B$ + 4 × C^6-*C*H(CH_3)$_A$(CH_3)$_B$), 123.7 (s, 4C, 4 × *C*^{3}-H, C_6H_3), 123.9 (s, 4C, 4 × *C*^{5}-H, C_6H_3), 124.3

[S3] The ^{1}H NMR spectroscopic data of **2-Cl** compare well with those reported in ref. [S1]. However, the C^3-*H*, C^4-*H* and C^5-*H* signals were only described as one multiplet in ref. [S1].

[S4] The elemental analysis of **2-Br** was repeated several times with different samples of **2-Br**, which were all tested before to be completely soluble in benzene-d_6 and pure by NMR spectroscopy (cf. Figures S1 – S3). All samples yielded consistently lower C values (by ca. 1 %) probably due to incomplete combustion.

(s, 4C, 2 × $C^{4,5}$-H, NCH), 130.2 (s, 4C, 4 × C^4-H, C_6H_3), 135.9 (s, 4C, 4 × C^1, C_6H_3), 146.2 (s, 4C, 4 × C^2, C_6H_3), 146.9 (s, 4C, 4 × C^6, C_6H_3), 177.1 (s, 2C, 2 × C^2-Si).

$^{29}Si\{^1H\}$ NMR (C_6D_6, 59.63 MHz, 298 K, ppm, Figure S3): δ_{Si} = 34.9 (s, 2Si).

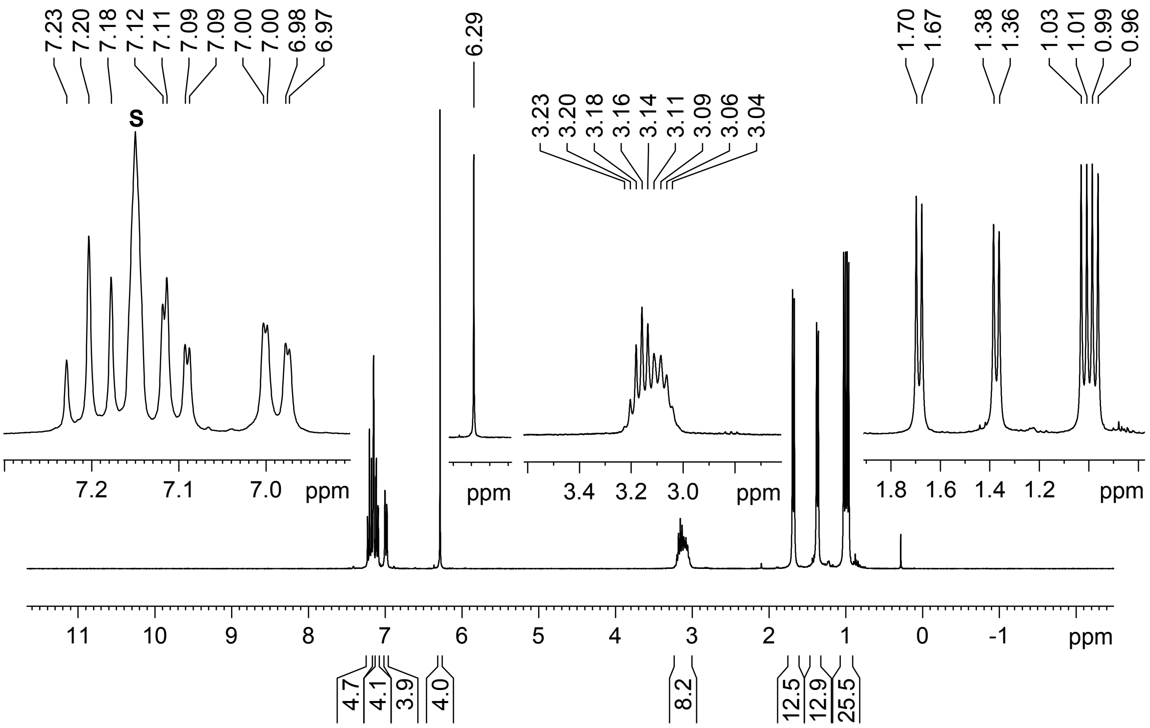

Figure S1. ^1H NMR (300.1 MHz) spectrum of **2-Br** in benzene-d_6 at 298 K. The signal of the deuterated solvent is marked with the character **S**. Enlarged excerpts are shown in the insets.

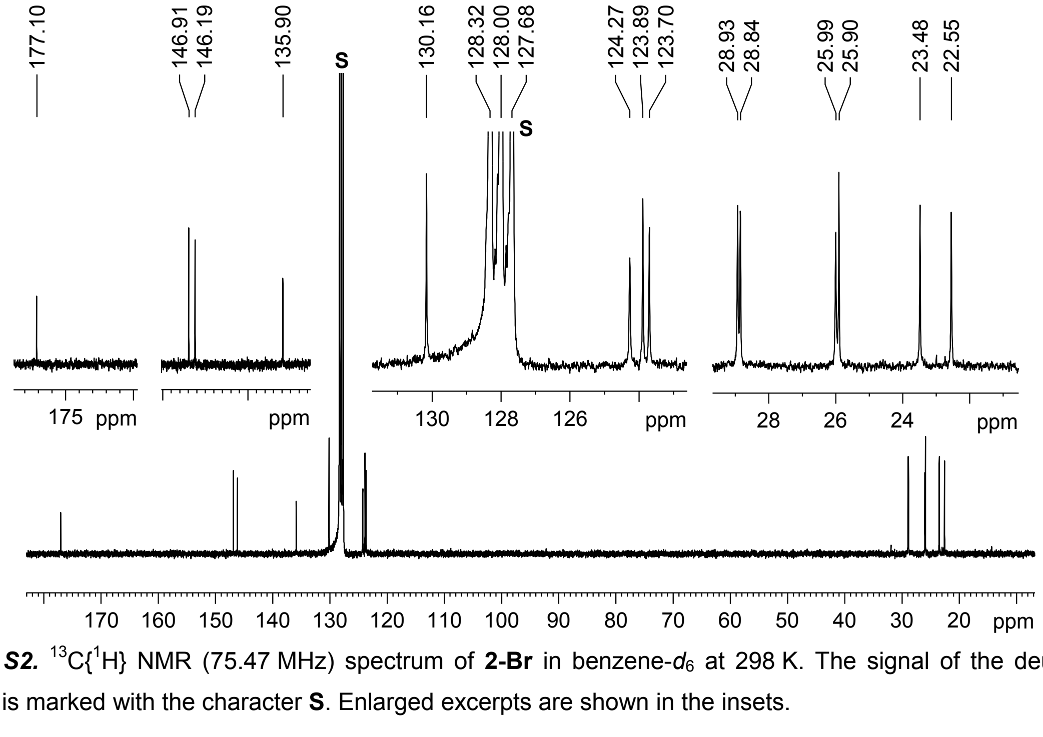

Figure S2. $^{13}C\{^1H\}$ NMR (75.47 MHz) spectrum of **2-Br** in benzene-d_6 at 298 K. The signal of the deuterated solvent is marked with the character **S**. Enlarged excerpts are shown in the insets.

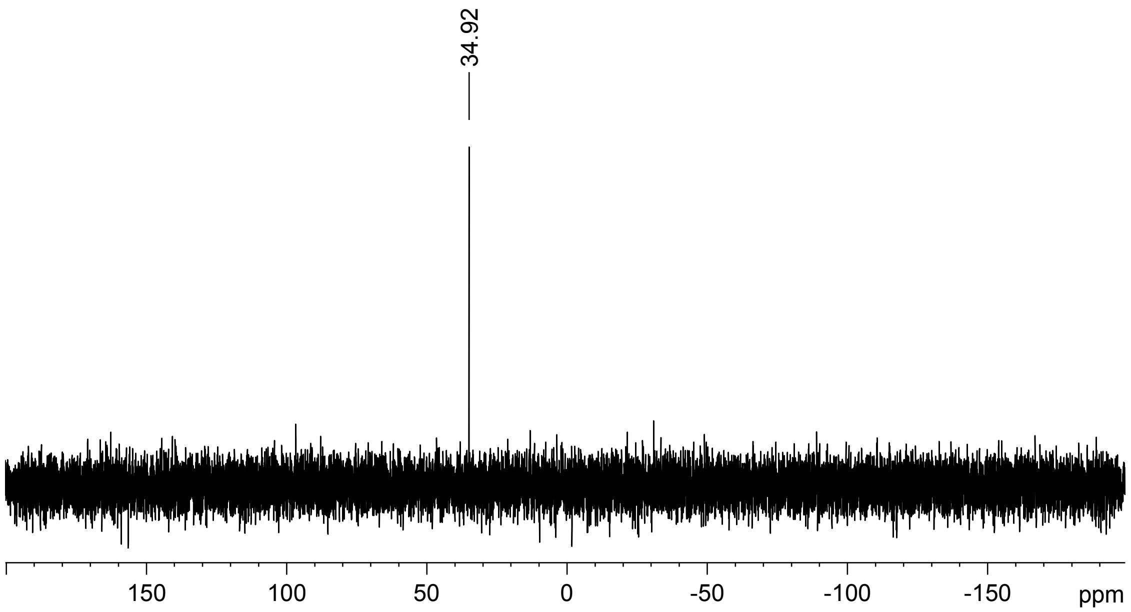

Figure S3. $^{29}Si\{^{1}H\}$ NMR (59.63 MHz) spectrum of **2-Br** in benzene-d_6 at 298 K.

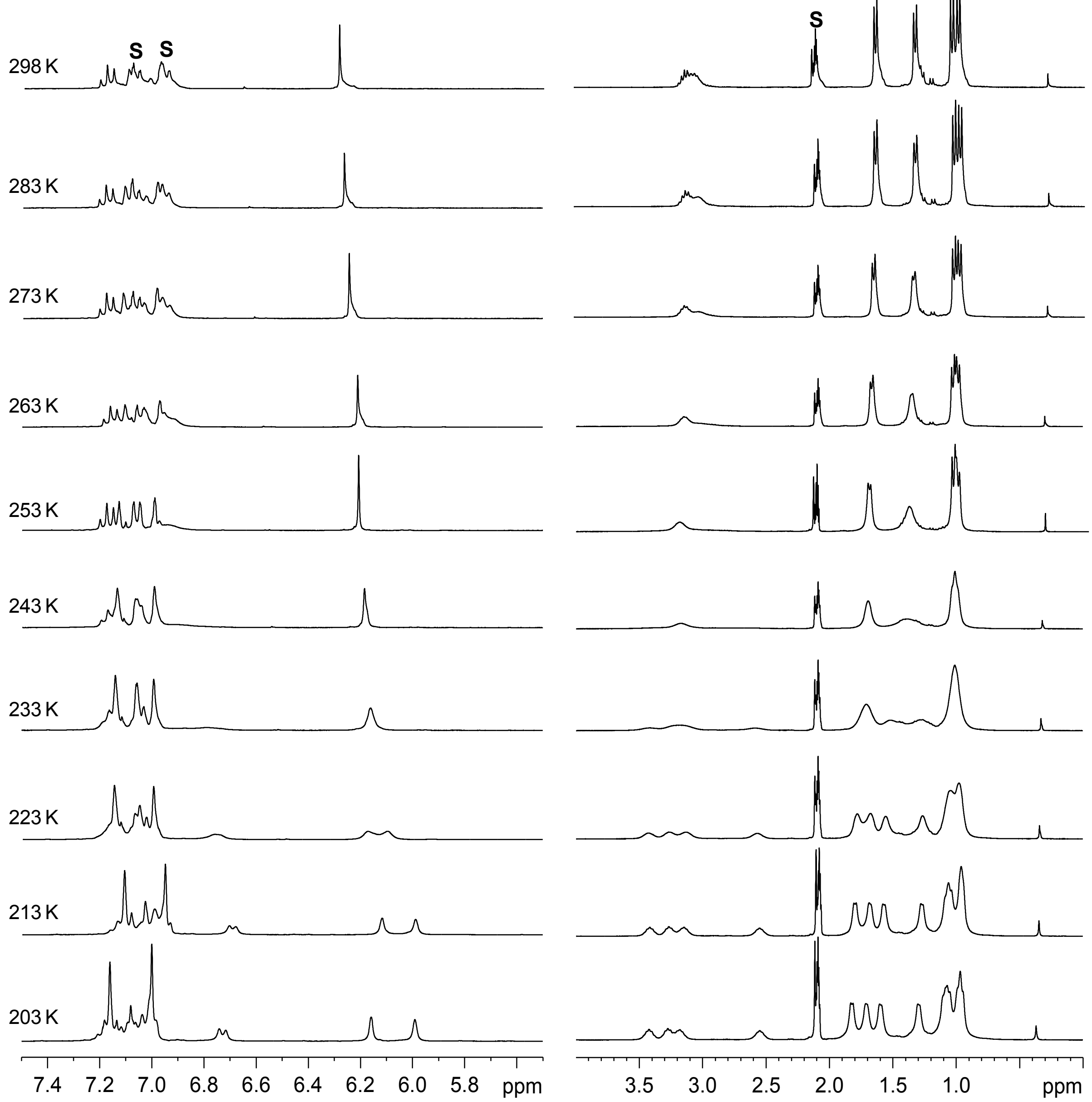

Figure S4. Excerpts of the variable temperature ^{1}H NMR (300.1 MHz) spectra of **2-Br** in toluene-d_8 in the range of 203 – 298 K. The signals of the deuterated solvent are marked with the character **S**.

The standard Gibbs energy of activation ($\Delta G^{\neq}$) for the hindered rotation of the NHC-substituents about the Si–C_{NHC} bonds of **2-Br** was calculated using the equation $\Delta G^{\neq} = 0.01914 \cdot T_c \cdot [9.972 + \lg(T_c/\Delta\nu)]$ (T_c = coalescence temperature, $\Delta\nu$ = distance of the signals in the slow exchange limit spectrum) and the values determined from the coalescence of the $C^{4,5}$-H signals of **2-Br** (T_c = 228 K, $\Delta\nu$ = 51 Hz) (Figure S4). $\Delta G^{\neq}$ amounts to 46 kJ mol^{-1}.[S5]

[S5] H. S. Gutowsky, C. H. Holm, *J. Chem. Phys.* **1956**, *25*, 1228.

2.3 Thermolysis of 2-Br

The thermal behaviour of compound **2-Br** in solution was studied by dissolving 10 mg of **2-Br** in 0.5 mL C_6D_6 and recording the ^1H NMR spectra at 25 °C and after heating of the solution at 85 °C for 2 h and 5 h (Figure S5). The NMR spectra after heating of the solution showed the thermal decomposition of **2-Br** (**a**) to $SiBr_2$(Idipp) (**b**) and Idipp (**c**) (Figure S5). The decomposition was accompanied by precipitation of a colorless solid of unknown composition.

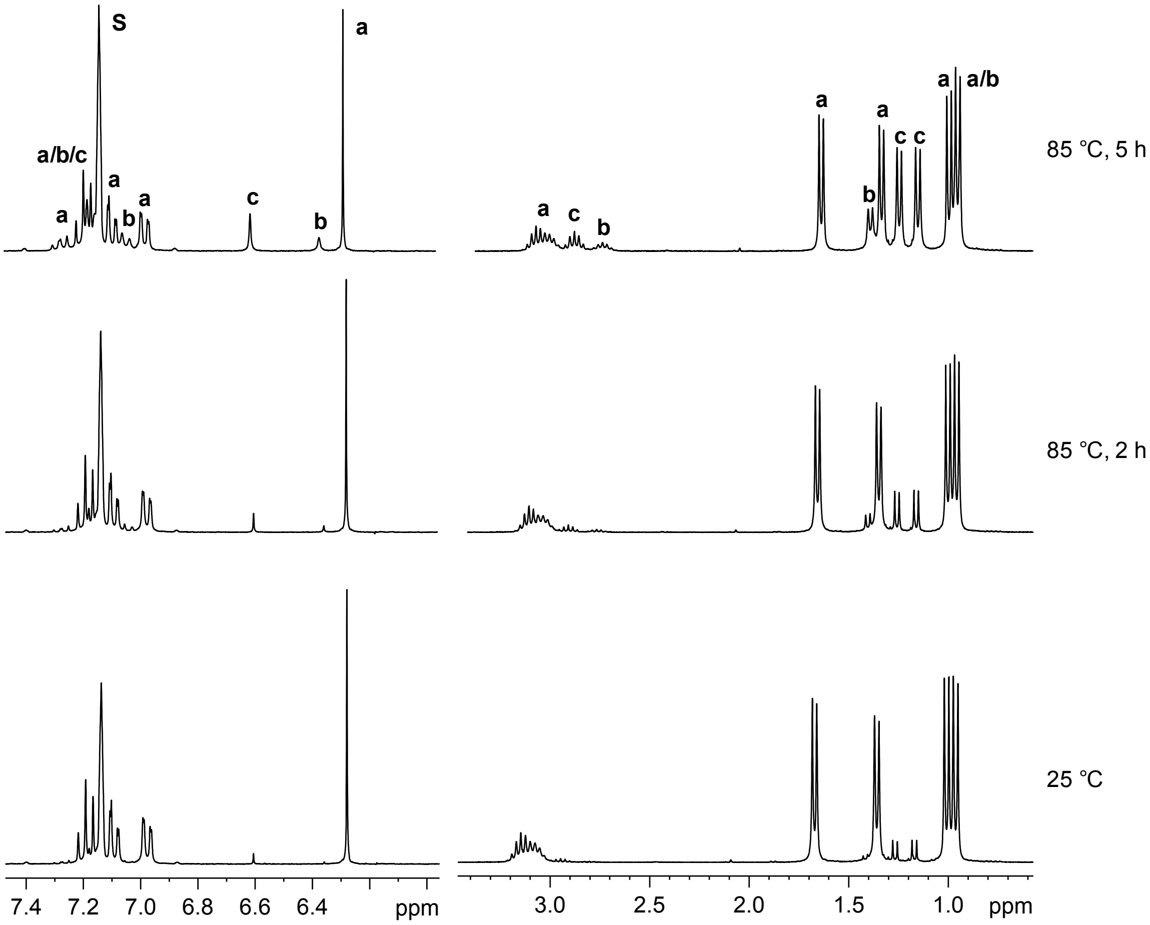

Figure S5. Excerpts of the ^1H NMR (300.1 MHz) spectra of **2-Br** in C_6D_6 recorded at 25 °C (bottom) and after heating the sample at 85 °C for 2 h (middle) and 5 h (top).The thermal decomposition of **2-Br** (**a**) at 85 °C leads to $SiBr_2$(Idipp) (**b**) and Idipp (**c**). The signals of the deuterated solvent are marked with the character **S**.

2.4 $Si_2I_2(Idipp)_2 \cdot 0.5(n\text{-}C_6H_{14})$ (2-I·0.5(n-C_6H_{14}))

A dark red suspension of **1** (350 mg, 0.42 mmol) in 30 mL of THF was cooled to −70 °C. A solution of 118 mg (0.42 mmol) of 1,2-diiodoethane in 10 mL of THF was added dropwise to the suspension of **1** by means of a stainless steel cannula (∅ = 1 mm) over a period of 15 minutes, whereupon the solid dissolved. The colour of the solution changed from dark red to light red and evolution of ethene gas was observed. The reaction solution was stirred under

static vacuum for one hour at −70 °C and for another hour at ambient temperature. The solution was then concentrated under vacuum to approximately 5 mL, whereupon precipitation of a light red solid was observed. The suspension was stored at −60 °C for seven days to complete the precipitation of **2-I**. The red precipitate was isolated by filtration at −60 °C, washed with *n*-hexane (2 × 5 mL) at this temperature and dried under vacuum at ambient temperature for two hours to afford the *n*-hexane hemisolvate **2-I**·0.5(*n*-C_6H_{14}) as a light red, extremely air-sensitive solid. Yield: 290 mg (0.26 mmol, 61 %).

Compound **2-I**·0.5(*n*-C_6H_{14}) turns brown upon heating to 190 °C and melts under decomposition at 208 °C to form a dark brown mass.

Elemental analysis calcd (%) for $C_{54}H_{72}I_2N_4Si_2 \cdot 0.5\ C_6H_{14}$ (1130.24): C 60.57, H 7.05, N 4.96; found: C 59.06, H 6.87, N 4.65 %.[S6]

^{1}H NMR (300.1 MHz, C_6D_6, 298 K, ppm, Figures S6 and S7): δ_H = 0.86 (t, ^3J(H,H) = 6.8 Hz, 3H, 2 × CH_3, 0.5(*n*-C_6H_{14})), 0.94 (d, ^3J(H,H) = 6.8 Hz, 12H, 4 × C^2-CH(CH_3)$_A$(CH$_3$)$_B$), 0.99 (d, 12H, ^3J(H,H) = 6.8 Hz, 4 × C^6-CH(CH_3)$_A$(CH$_3$)$_B$), 1.18 – 1.29 (m, 4H, 4 × CH_2, 0.5(*n*-C_6H_{14})), 1.41 (br, $\Delta\nu_{½}$ = 21 Hz, 12H, 4 × C^2-CH(CH$_3$)$_A$(CH_3)$_B$), 1.71 (br d, ^3J(H,H) = 6.4 Hz, 12H, 4 × C^6-CH(CH$_3$)$_A$(CH_3)$_B$), 3.20 (br, $\Delta\nu_{½}$ = 40 Hz, 8H, 4 × C^2-CH(CH$_3$)$_A$(CH$_3$)$_B$ + 4 × C^6-CH(CH$_3$)$_A$(CH$_3$)$_B$), 6.29 (s, 4H, 2 × $C^{4,5}$-*H*), 6.99 (br, $\Delta\nu_{½}$ = 17 Hz, 4H, 4 × C^3-*H*, C_6H_3), 7.10 (dd, ^3J(H,H) = 7.6 Hz, ^4J(H,H) = 1.2 Hz, 4H, 4 × C^5-*H*, C_6H_3), 7.22 (t, ^3J(H,H) = 7.6 Hz, 4H, 4 × C^4-*H*, C_6H_3).

^{13}C{^{1}H} NMR (75.47 MHz, C_6D_6, 298 K, ppm, Figure S8): δ_C = 14.2 (s, 1C, 2 × *C*H$_3$, 0.5(*n*-C_6H_{14})), 22.7 (s, 1C, 2 × *C*H$_2$, 0.5(*n*-C_6H_{14})), 22.8 (s, 4C, 4 × C^6-CH(CH$_3$)$_A$(*C*H$_3$)$_B$), 23.7 (br, $\Delta\nu_{½}$ = 14.4 Hz, 4C, 4 × C^2-CH(CH$_3$)$_A$(*C*H$_3$)$_B$), 26.1 (s, 8C, 4 × C^2-CH(*C*H$_3$)$_A$(CH$_3$)$_B$ + 4 × C^6-CH(*C*H$_3$)$_A$(CH$_3$)$_B$), 28.8 (s, 4C, 4 × C^6-*C*H(CH$_3$)$_A$(CH$_3$)$_B$), 29.0 (s, 4C, 4 × C^2-*C*H(CH$_3$)$_A$(CH$_3$)$_B$), 34.4 (s, 1C, 2 × *C*H$_2$, 0.5(*n*-C_6H_{14})), 123.9 (s, 4C, 4 × C^5-H, C_6H_3), 124.3 (s, 4C, 4 × C^3-H, C_6H_3), 124.5 (br, $\Delta\nu_{½}$ = 25 Hz, 4C, 2 × $C^{4,5}$-H, NCH), 130.5 (s, 4C, 4 × C^4-H, C_6H_3), 136.3 (s, 4C, 4 × C^1, C_6H_3), 146.0 (s, 4C, 4 × C^2, C_6H_3), 147.2 (s, 4C, 4 × C^6, C_6H_3), 174.4 (s, 2C, 2 × C^2-Si).

^{29}Si{^{1}H} NMR (59.63 MHz, C_6D_6, 298 K, ppm, Figure S9): δ_{Si} = 18.7 (s, 2Si).

[S6] The elemental analysis of **2-I**·0.5(*n*-C_6H_{14}) was repeated several times with different samples of **2-I**·0.5(*n*-C_6H_{14}), which were all found before to be completely soluble in benzene-*d*$_6$ and pure by NMR spectroscopy (cf. Figures S5 – S8). All samples yielded consistently lower C values (by ca. 1 %) probably due to incomplete combustion.

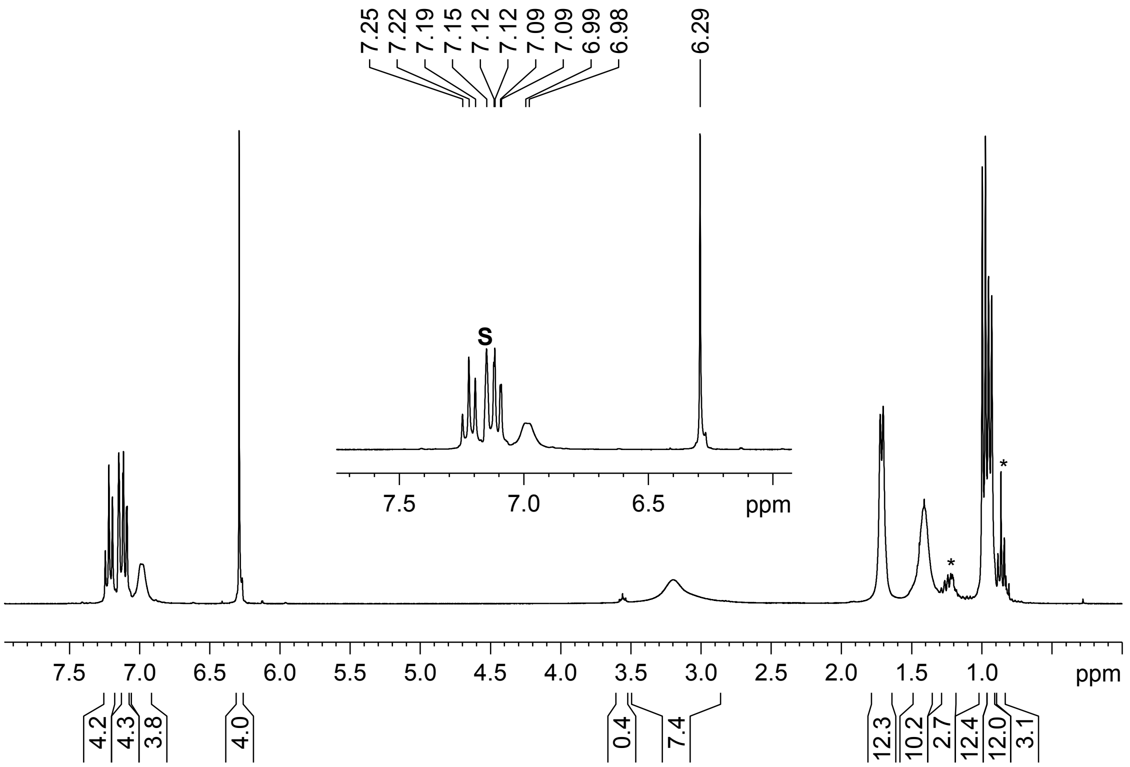

Figure S6. ^{1}H NMR (300.1 MHz) spectrum of **2-I**·0.5(n-C_6H_{14}) in benzene-d_6 at 298 K. The signals marked with an asterisk (*) arise from the co-crystallised n-hexane. The inset shows the aryl region of the spectrum. The signal of the deuterated solvent is marked with the character **S**.

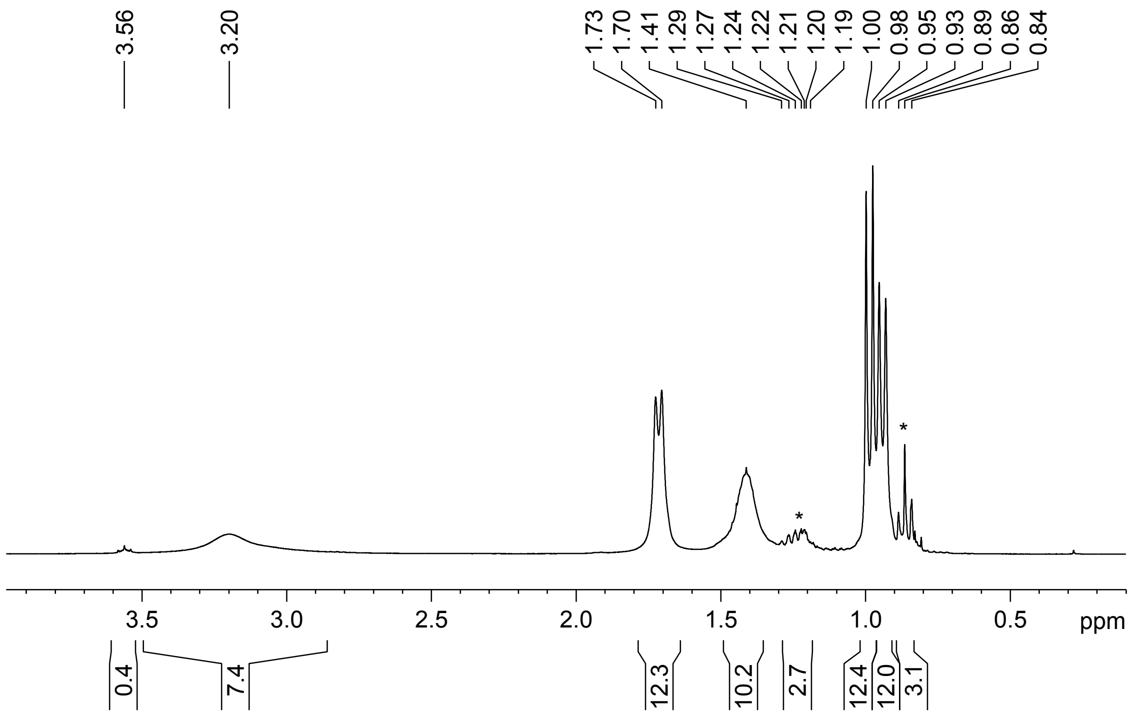

Figure S7. Alkyl region of the ^{1}H NMR (300.1 MHz) spectrum of **2-I**·0.5(n-C_6H_{14}) in benzene-d_6 at 298 K. The signals marked with an asterisk (*) arise from the co-crystallised n-hexane.

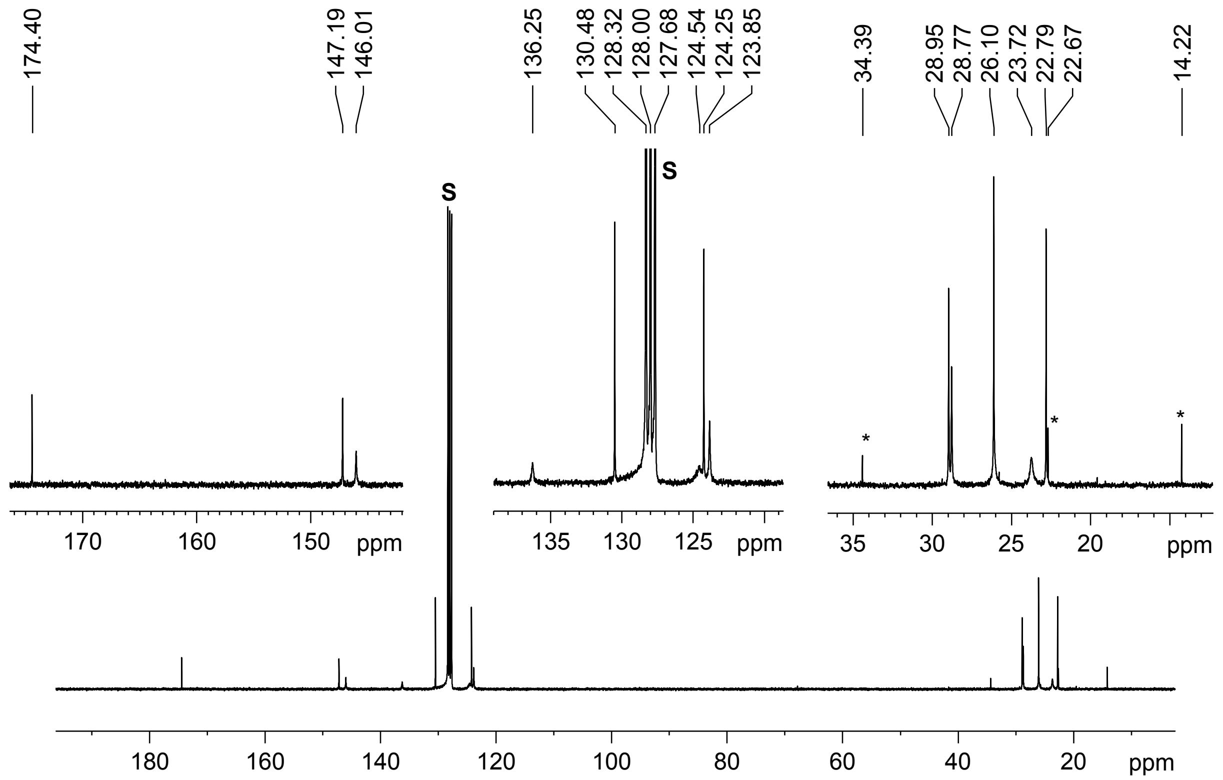

Figure S8. $^{13}C\{^1H\}$ NMR (75.47 MHz) spectrum of **2-I**·0.5(*n*-C_6H_{14}) in benzene-d_6 at 298 K. The signal of the deuterated solvent is marked with the character **S**. Enlarged excerpts are shown in the insets. The signals marked with an asterisk (*) arise from the co-crystallised *n*-hexane.

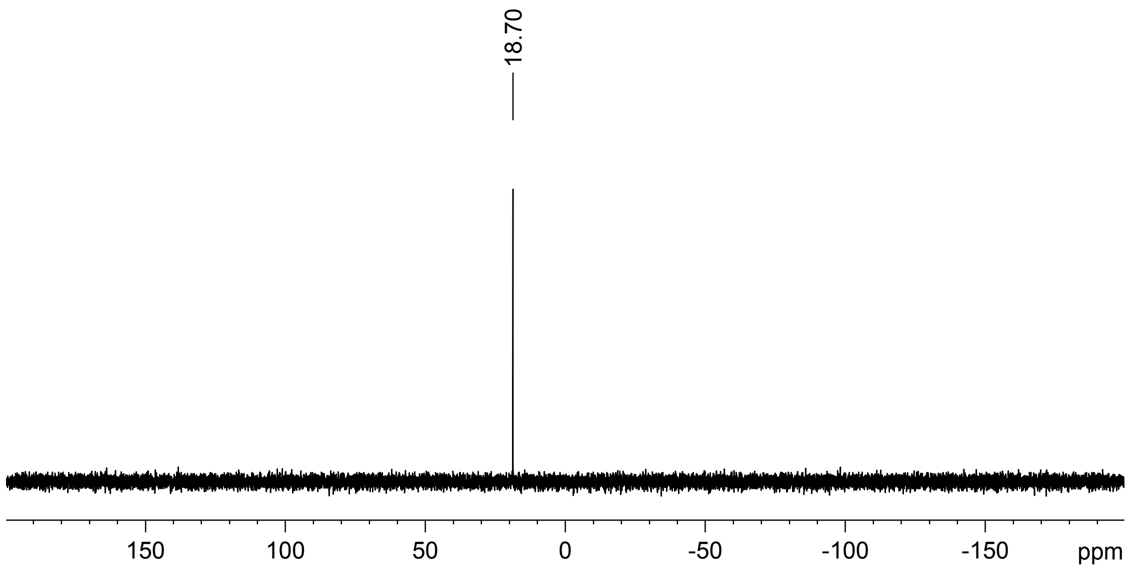

Figure S9. $^{29}Si\{^1H\}$ NMR (59.63 MHz) spectrum of **2-I**·0.5(*n*-C_6H_{14}) in benzene-d_6 at 298 K.

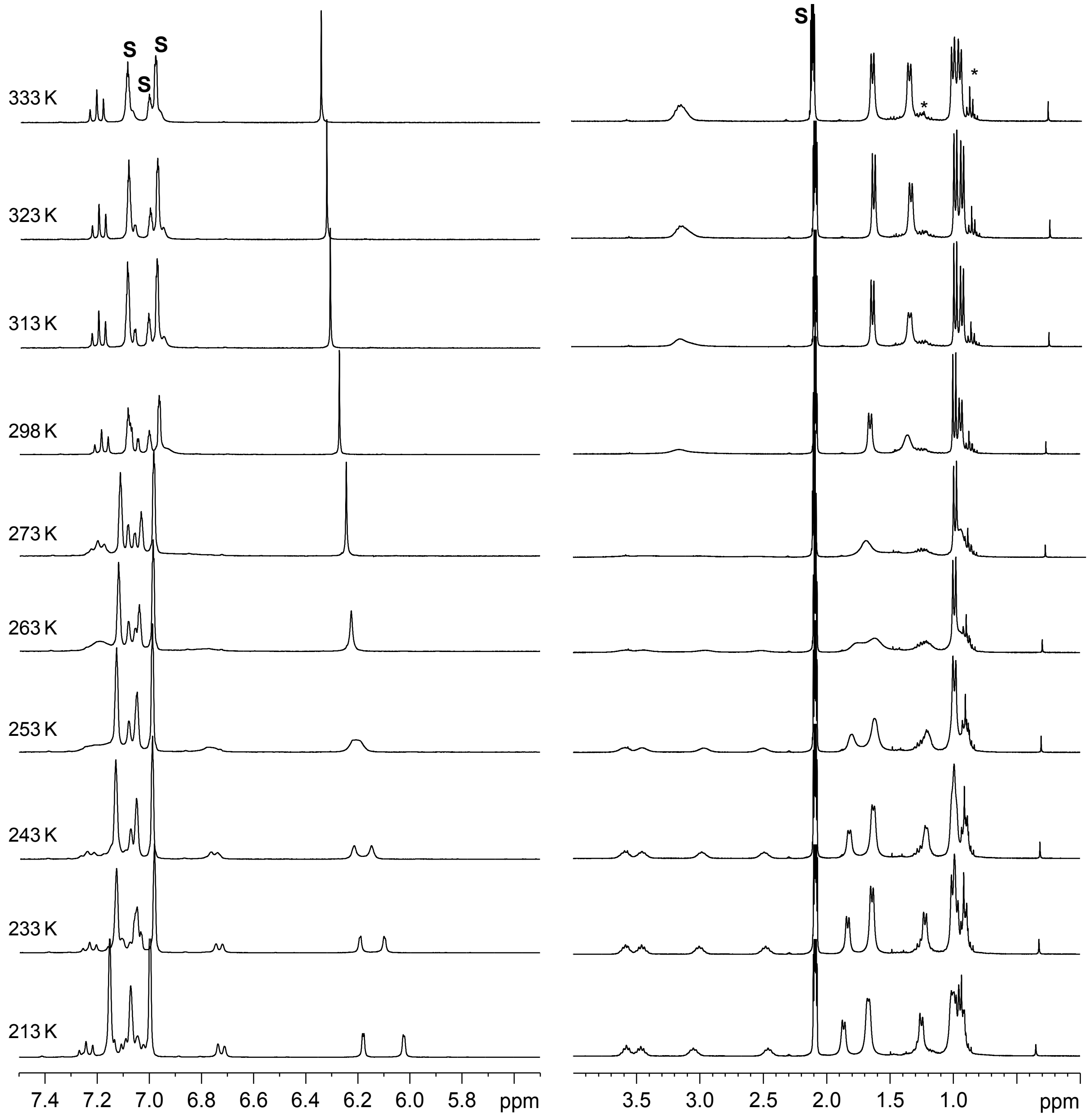

Figure S10. Excerpts of the variable temperature ^{1}H NMR (300.1 MHz) spectra of **2-I**·0.5(*n*-C_6H_{14}) in toluene-d_8 in the range of 203 – 333 K. The signals of the deuterated solvent are marked with the character **S**. The signals marked with an asterisk (*) arise from the co-crystallised *n*-hexane.

The standard Gibbs energy of activation ($\Delta G^{\neq}$) for the hindered rotation of the NHC-substituents about the Si–C_{NHC} bonds of **2-I**·0.5(*n*-C_6H_{14}) was calculated using the equation $\Delta G^{\neq} = 0.01914 \cdot T_c \cdot [9.972 + \lg(T_c/\Delta\nu)]$ (T_c = coalescence temperature, $\Delta\nu$ = distance of the signals in the slow-exchangelimit spectrum) and the values determined from the coalescence of the $C^{4,5}$-*H* signals of **2-I**·0.5(*n*-C_6H_{14}) (T_c = 248 K, $\Delta\nu$ = 47 Hz) (Figure S10). $\Delta G^{\neq}$ amounts to 51 kJ mol^{-1}.[S5]

2.5 [Si_2(I)(Idipp)$_2$][B(C_6F_5)$_4$]·(C_6H_5F) (3·(C_6H_5F))

A solution of [Li(Et_2O)$_{2.5}$][B(C_6F_5)$_4$] (168 mg, 0.19 mmol) in 7 mL of fluorobenzene was added dropwise to a solution of **2-I**·0.5(*n*-C_6H_{14}) (210 mg, 0.19 mmol) in 8 mL of fluorobenzene at ambient temperature. Immediately, a dark red solution containing a small amount of a white solid was formed. The reaction mixture was stirred at ambient temperature for two hours, then concentrated under vacuum to 4 mL, and 1 mL of *n*-hexane was added. The dark red solution was filtered from a small amount of a white solid (LiI). 3 mL of *n*-hexane were added to the filtrate and the biphasic mixture was stirred for a few seconds. Storage of the red solution at ambient temperature for 14 hours afforded a dark red, crystalline solid. The red supernatant was decanted off with a syringe and the solid was washed with *n*-hexane (2 × 2 mL) at ambient temperature and dried under vacuum for two hours to afford the fluorobenzene monosolvate **3**·(C_6H_5F) as dark red, plate-shaped crystals, which were suitable for X-Ray diffraction (vide infra). Yield: 204 mg (0.12 mmol, 62 %). Compound **3**·(C_6H_5F) melts under decomposition at 208 °C to form a red-brown mass.

Elemental analysis calcd (%) for $C_{78}H_{72}BF_{20}IN_4Si_2$·$C_6H_5F$ (1735.79): C 58.13, H 4.47, N 3.23; found: C 57.71, H 4.67, N 3.08 %.

^{1}H NMR (300.1 MHz, THF-d_8, 203 K, ppm, Figures S11 and S12): δ_H = 0.93 – 1.12 (br m, $\Delta\nu_{½}$ = 24 Hz, 48H, 4 × $C^{2,6}$-CH(C*H*$_3$)$_2$), 2.27 – 2.50 (br m, $\Delta\nu_{½}$ = 49 Hz, 8H, 4 × $C^{2,6}$-C*H*(CH$_3$)$_A$(CH$_3$)$_B$), 7.14 – 7.22 (m, 3H, C_6H_5F), 7.26 (d, ^3J(H,H) = 7.8 Hz, 4H, 2 × $C^{3,5}$-*H*, C_6H_3, (Idipp)$_X$), 7.27 (d, ^3J(H,H) = 7.8 Hz, 4H, 2 × $C^{3,5}$-*H*, C_6H_3, (Idipp)$_Y$), 7.38 – 7.45 (m, 2H, C_6H_5F), 7.54 (t, ^3J(H,H) = 7.8 Hz, 2H, 2 × C^4-*H*, (Idipp)$_Y$), 7.58 (t, ^3J(H,H) = 7.8 Hz, 2H, 2 × C^4-*H*, (Idipp)$_X$), 8.11 (s, 2H, $C^{4,5}$-*H*, (Idipp)$_Y$), 8.30 (s, 2H, $C^{4,5}$-*H*, (Idipp)$_X$).

^{1}H NMR (300.1 MHz, THF-d_8, 298 K, ppm, Figure S13): δ_H = 1.06 (d, ^3J(H,H) = 6.8 Hz, 24H, 4 × $C^{2.6}$-CH(C*H*$_3$)$_A$(CH$_3$)$_B$), 1.11 (d, ^3J(H,H) = 6.8 Hz, 24H, 4 × $C^{2.6}$-CH(CH$_3$)$_A$(C*H*$_3$)$_B$), 2.45 (sept, ^3J(H,H) = 6.8 Hz, 8H, 4 × $C^{2.6}$-C*H*(CH$_3$)$_A$(CH$_3$)$_B$), 7.03 – 7.16 (m, 3H, C_6H_5F), 7.25 (d, ^3J(H,H) = 7.8 Hz, 8H, 4 × $C^{3,5}$-*H*, C_6H_3), 7.31 – 7.38 (m, 2H, C_6H_5F), 7.50 (t, ^3J(H,H) = 7.8 Hz, 4H, 4 × C^4-*H*, C_6H_3), 7.88 (s, 4H, 2 × $C^{4,5}$-*H*, NCH).

^{13}C{^{1}H} NMR (75.47 MHz, THF-d_8, 203 K, ppm, Figures S14 and S15): δ_C = 22.8, 23.7 (s each, 4C each, 2 × $C^{2.6}$-CH(CH$_3$)$_A$(*C*H$_3$)$_B$, (Idipp)$_X$ + 2 × $C^{2.6}$-CH(CH$_3$)$_A$(*C*H$_3$)$_B$, (Idipp)$_Y$), 25.9, 26.1 (s each, 4C each, 2 × $C^{2.6}$-CH(*C*H$_3$)$_A$(CH$_3$)$_B$, (Idipp)$_X$ + 2 × $C^{2.6}$-CH(*C*H$_3$)$_A$(CH$_3$)$_B$, (Idipp)$_Y$), 29.6 (s, 4C, 2 × $C^{2,6}$-*C*H(CH$_3$)$_A$(CH$_3$)$_B$, (Idipp)$_Y$), 30.0 (s, 4C, 2 × $C^{2,6}$-*C*H(CH$_3$)$_A$(CH$_3$)$_B$, (Idipp)$_X$), 116.0 (d, ^2J(F,C) = 21.0 Hz, 2C, $C^{2,6}$-H, C_6H_5F), 124.4 (br, $\Delta\nu_{½}$ = 120 Hz, 4C, 4 × C^1-B, C_6F_5), 125.2 (d, ^4J(F,C) = 3.1 Hz, 1C, C^4-H, C_6H_5F), 125.3, 125.4 (s each, 2C each, 2 × $C^{3,5}$-H, C_6H_3, (Idipp)$_X$ + 2 × $C^{3,5}$-H, C_6H_3, (Idipp)$_Y$), 128.4 (s, 2C, 2 × $C^{4,5}$-H, (Idipp)$_X$), 128.5 (s, 2C, 2 × $C^{4,5}$-H, (Idipp)$_Y$), 131.2 (d, ^3J(F,C) = 7.9 Hz, 2C, $C^{3,5}$-F, C_6H_5F), 131.6 (s, 2C, 2 × C^4-H, (Idipp)$_Y$), 132.5 (s, 2C, 2 × C^4-H, (Idipp)$_X$), 133.5 (s, 2C, 2 × C^1, C_6H_3,

(Idipp)$_X$), 134.7 (s, 2C, 2 × *C*[1], C_6H_3, (Idipp)$_Y$), 136.9 (dm, ^1J(C,F) = 245 Hz, 8C, 4 × $C^{3,5}$-F, C_6F_5), 139.0 (dm, ^1J(C,F) = 245 Hz, 4C, 4 × C^4-F, C_6F_5), 145.6 (s, 4C, 2 × $C^{2,6}$, C_6H_3, (Idipp)$_Y$), 145.9 (s, 4C, 2 × $C^{2,6}$, C_6H_3, (Idipp)$_X$), 148.8 (dm, ^1J(C,F) = 241 Hz, 8C, 4 × $C^{2,6}$-F, C_6F_5), 153.6 (s, 1C, C^2-Si, (Idipp)$_X$), 163.6 (d, ^1J(F,C) = 244.2 Hz, 1C, C^1-F, C_6H_5F), 172.2 (s, 1C, C^2-Si, (Idipp)$_Y$).

$^{13}C\{^1H\}$ NMR (75.47 MHz, THF-d_8, 298 K, ppm, Figures S16 and S17): δ_C = 23.4 (s, 8C, 4 × $C^{2.6}$-CH(CH_3)$_A$(CH_3)$_B$), 25.8 (s, 8C, 4 × $C^{2.6}$-CH(CH_3)$_A$(CH_3)$_B$), 29.9 (s, 8C, 4 × $C^{2,6}$-*C*H(CH_3)$_A$(CH_3)$_B$), 115.9 (d, ^2J(F,C) = 21 Hz, 2C, $C^{2,6}$-H, C_6H_5F), 124.9 (d, ^4J(F,C) = 2.9 Hz, 1C, C^4-H, C_6H_5F), 125.2 (br, $\Delta\nu_{½}$ ca. 180 Hz, 4C, 4 × C^1-B, C_6F_5), 125.6 (s, 8C, 4 × $C^{3,5}$-H, C_6H_3), 128.2 (s, 4C, 2 × $C^{4,5}$-H, NCH), 130.9 (s, ^3J(F,C) = 7.7 Hz, 2C, $C^{3,5}$-H, C_6H_5F), 132.1 (s, 4C, 4 × C^4-H, C_6H_3), 134.3 (s, 4C, 4 × C^1, C_6H_3), 137.1 (dm, ^1J(F,C) = 243 Hz, 8C, 4 × $C^{3,5}$-F, C_6F_5), 139.1 (dm, ^1J(F,C) = 243 Hz, 4C, 4 × C^4-F, C_6F_5), 146.1 (s, 8C, 4 × $C^{2,6}$, C_6H_3), 149.2 (dm, ^1J(F,C) = 243 Hz, 8C, 4 × $C^{2,6}$-F, C_6F_5), 163.9 (d, ^1J(F,C) = 244 Hz, 1C, C^1-F, C_6H_5F).

$^{29}Si\{^1H\}$ NMR (59.63 MHz, THF-d_8, 203 K, ppm, Figure S18): δ_{Si} = −26.4 (s, 1Si, *Si*-I), 75.3 (s, 1Si).

$^{11}B\{^1H\}$ NMR (96.29 MHz, THF-d_8, 298 K, ppm): δ_B = −16.6 (s, 1B).

$^{19}F\{^1H\}$ NMR (282.4 MHz, THF-d_8, 298 K, ppm, Figure S19): δ_F = −168.4 (m, 8F, 4 × $C^{3,5}$-*F*, C_6F_5), −165.0 (t, ^3J(F,F) = 20.5 Hz, 4F, 4 × C^4-*F*, C_6F_5), −132.6 (m, 8F, 4 × $C^{2,6}$-*F*, C_6F_5), −114.2 (s, 1F, C_6H_5F).

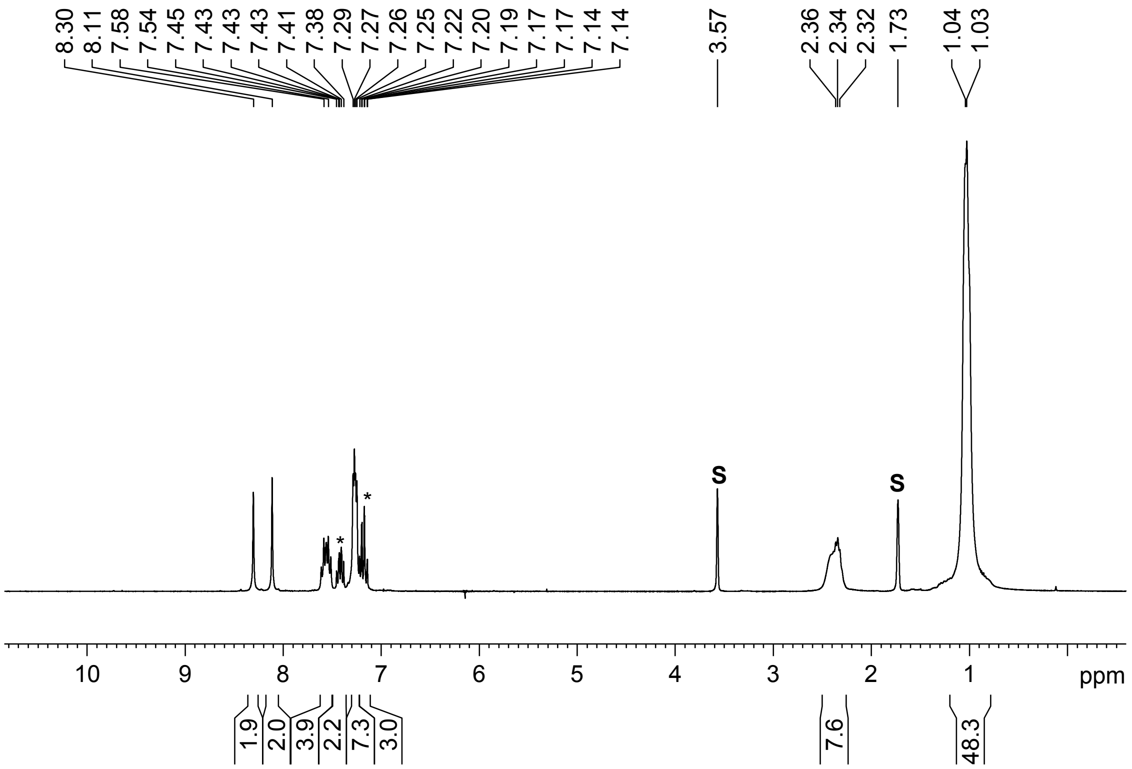

Figure S11. ^{1}H NMR (300.1 MHz) spectrum of **3**·(C_6H_5F) in THF-d_8 at 203 K. The signals of the deuterated solvent are marked with the character **S**. The signals marked with an asterisk (*) arise from the co-crystallised fluorobenzene.

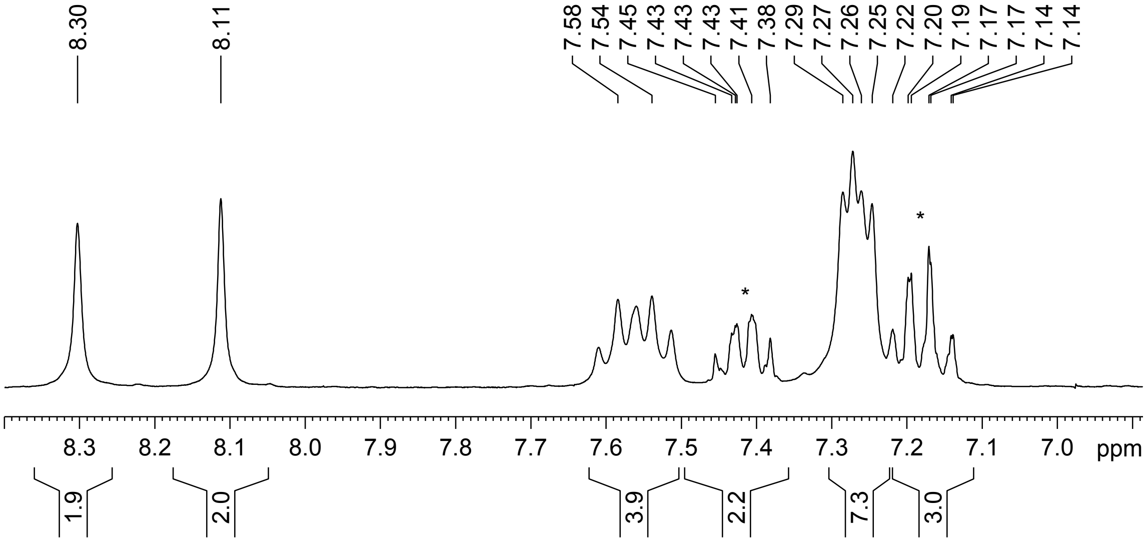

Figure S12. Aryl region of the ^{1}H NMR (300.1 MHz) spectrum of **3**·(C_6H_5F) in THF-d_8 at 203 K. The multiplet signals marked with an asterisk (*) arise from the co-crystallised fluorobenzene.

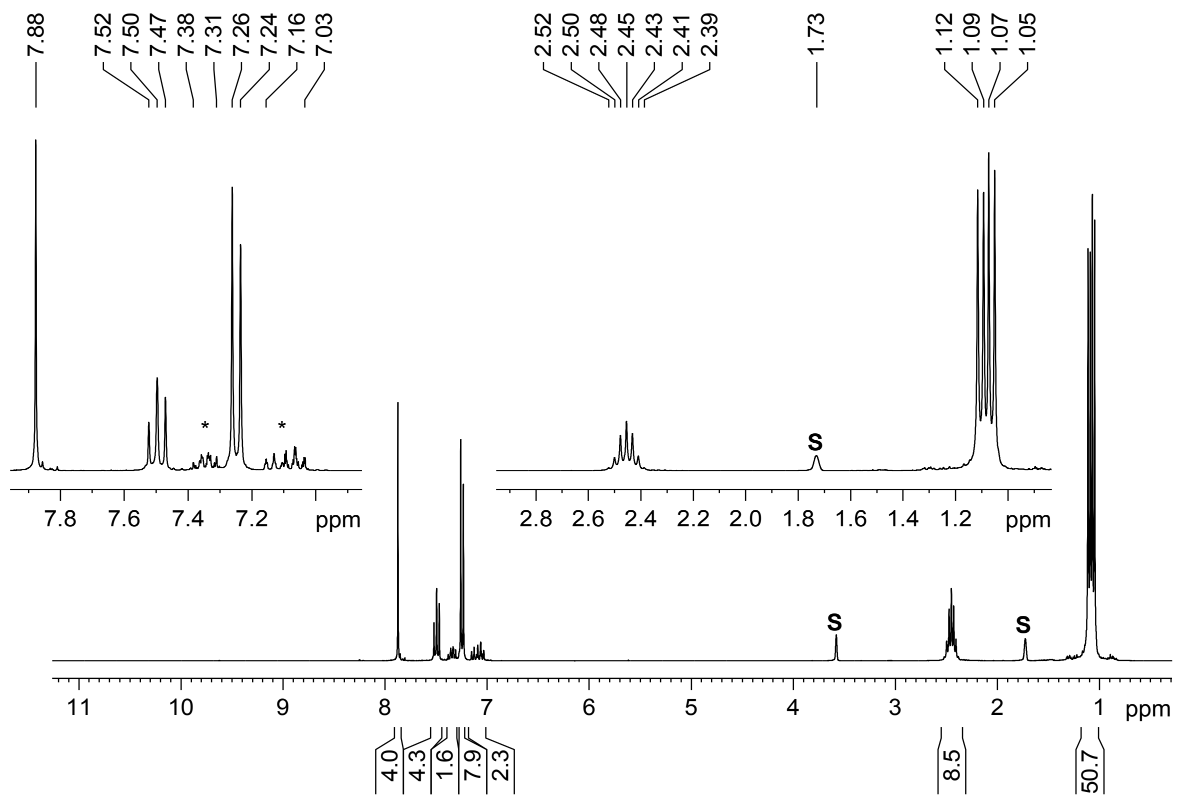

Figure S13. ^{1}H NMR (300.1 MHz) spectrum of **3**·(C_6H_5F) in THF-d_8 at 298 K. The signals of the deuterated solvent are marked with the character **S**. Enlarged excerpts are shown in the insets. The two multiplets marked with an asterisk (*) arise from the co-crystallised fluorobenzene.

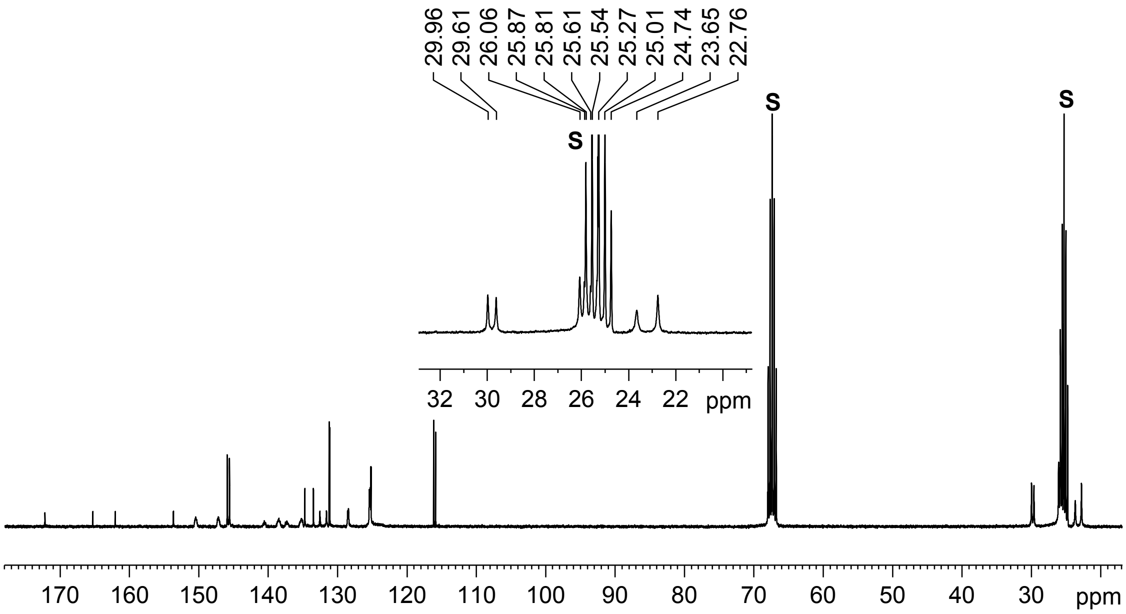

Figure S14. ^{13}C{^{1}H} NMR (75.47 MHz) spectrum of **3**·(C_6H_5F) in THF-d_8 at 203 K. The signals of the deuterated solvent are marked with the character **S**. An enlarged excerpt of the alkyl section of the spectrum is shown in the inset.

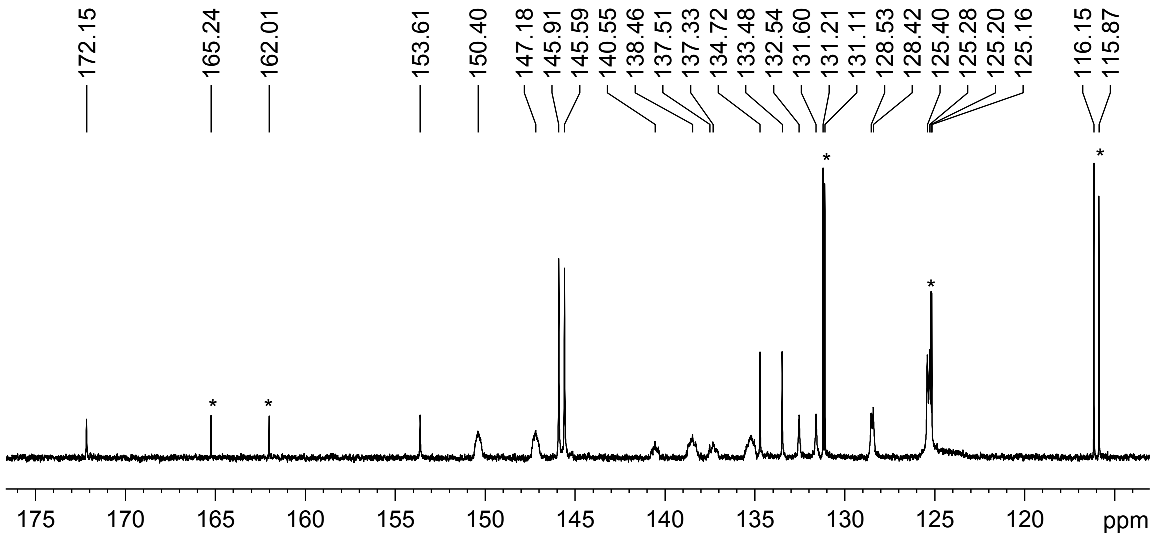

Figure S15. Aryl section of the $^{13}C\{^1H\}$ NMR (75.47 MHz) spectrum of **3**·(C_6H_5F) in THF-d_8 at 203 K. The signals marked with an asterisk (*) arise from the co-crystallised fluorobenzene.

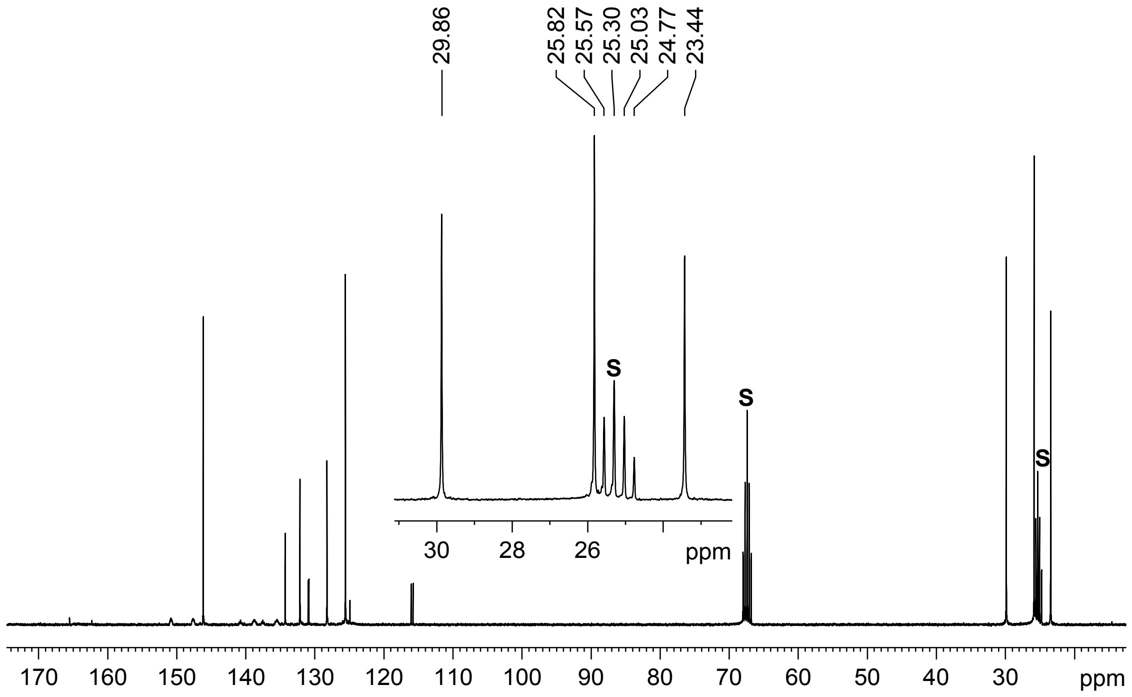

Figure S16. $^{13}C\{^1H\}$ NMR (75.47 MHz) spectrum of **3**·(C_6H_5F) in THF-d_8 at 298 K. The signals of the deuterated solvent are marked with the character **S**. An enlarged excerpt of the alkyl section of the spectrum is shown in the inset.

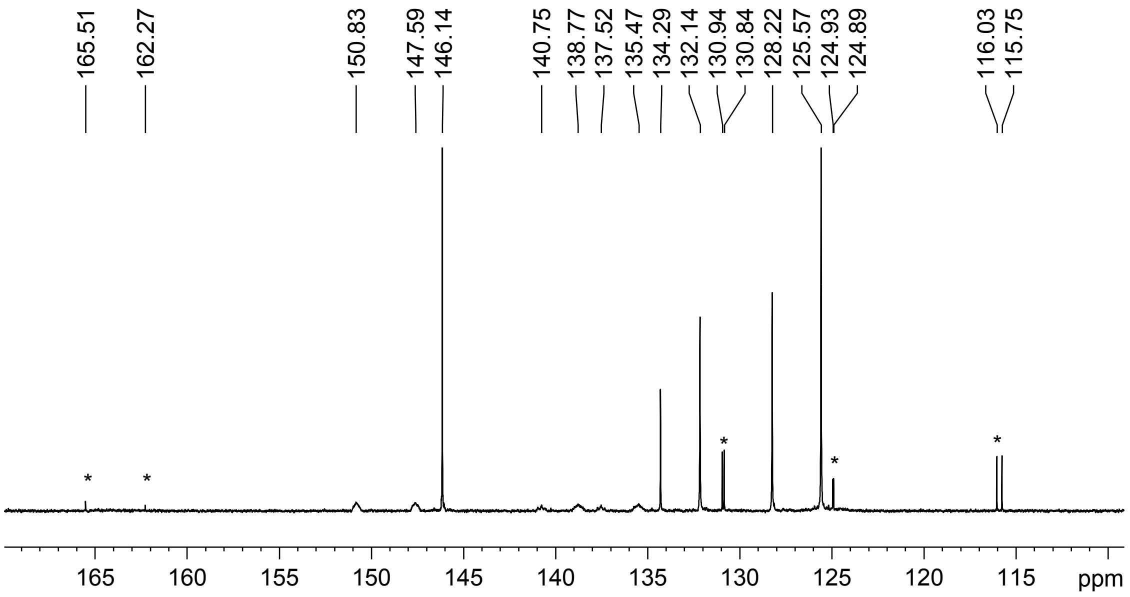

Figure S17. Aryl section of the ^{13}C{^{1}H} NMR (75.47 MHz) spectrum of **3**·(C_6H_5F) in THF-d_8 at 298 K. The signals marked with an asterisk (*) arise from the co-crystallised fluorobenzene.

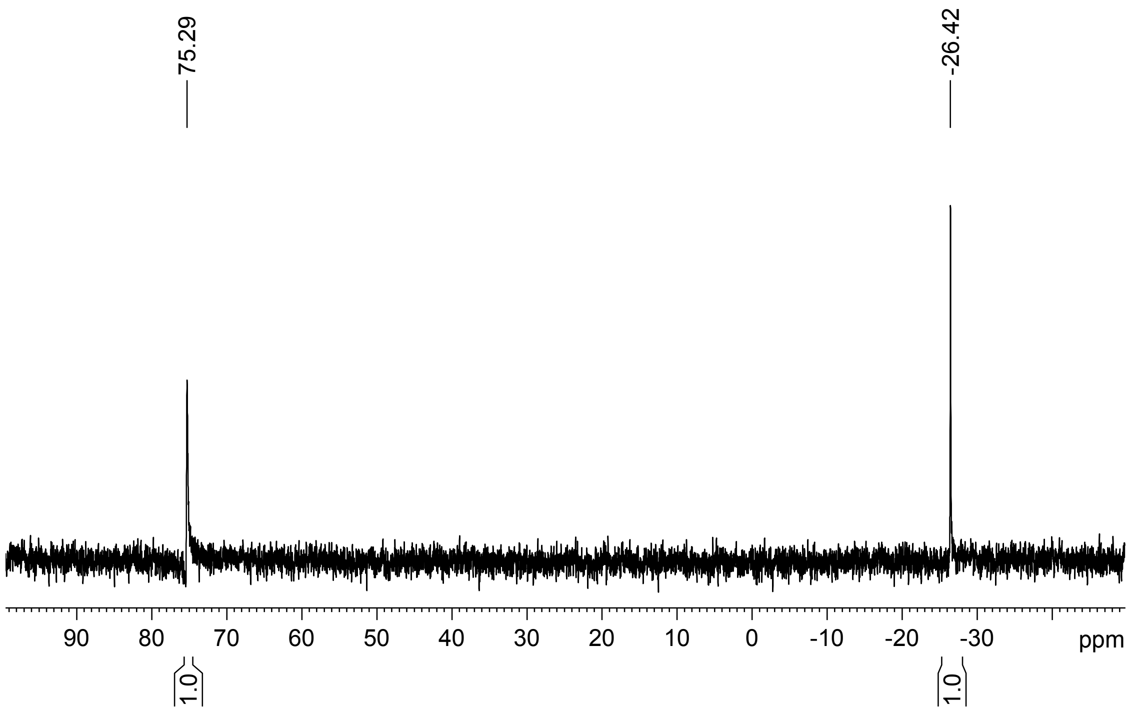

Figure S18. ^{29}Si{^{1}H} NMR (59.63 MHz) spectrum of **3**·(C_6H_5F) in THF-d_8 at 203 K.

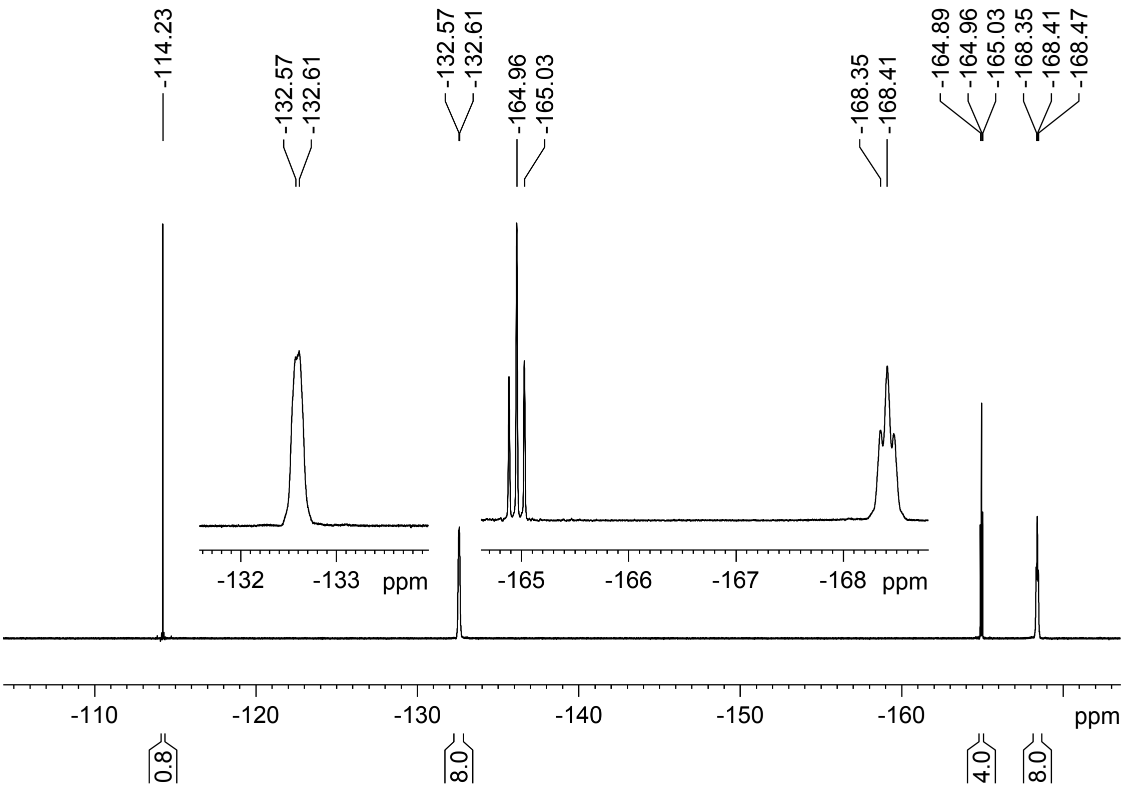

Figure S19. $^{19}F\{^{1}H\}$ NMR (282.4 MHz) spectrum of **3**·(C_6H_5F) in THF-d_8 at 298 K.

3. Determination of the standard Gibbs energy of activation for 3·(C_6H_5F)

The thermodynamic values ($\Delta G^{\neq}$, $\Delta H^{\neq}$, $\Delta S^{\neq}$) of the dynamic process of **3**·(C_6H_5F) were determined with variable temperature ^{1}H NMR spectroscopy from 203 K to 263 K (Figure S20).

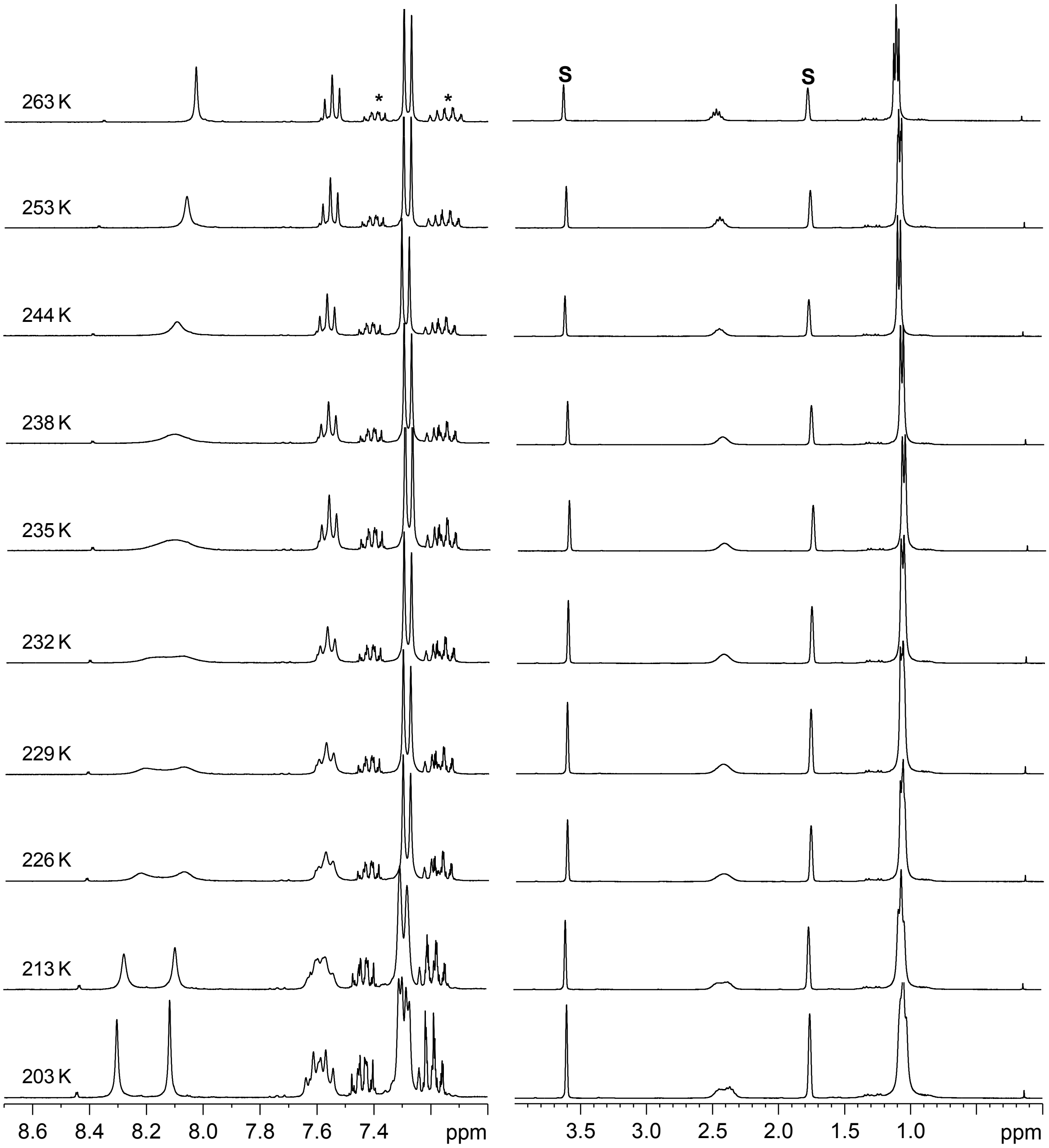

Figure S20. Excerpts of selected variable temperature ^{1}H NMR (300.1 MHz) spectra of **3**·(C_6H_5F) in THF-d_8 from 203 – 263 K showing the dynamic behaviour. The signals marked with **S** corresponds to the residual proton resonances of the deuterated solvent. The signals marked with an asterisk (*) arise from co-crystallised fluorobenzene.

The rate constants (k) were obtained from full line-shape analysis of the $C^{4,5}$-H signals using the NMR simulation program *g*NMR.[S7] The calculations were performed using standard methods of dynamic NMR spectroscopy.[S8] The rate constants (k) obtained from simulation are given in Table S1.

Table S1: Determined rate constants from 203 – 263 K.

T [K]	$1/T$ [1/K]	k [Hz]	ln(k/T)
203	$4.93\cdot10^{-3}$	3.93	−3.94457
208	$4.81\cdot10^{-3}$	7.24	−3.35792
213	$4.69\cdot10^{-3}$	13.7	−2.7439
218	$4.59\cdot10^{-3}$	24.8	−2.17365
223	$4.48\cdot10^{-3}$	44.2	−1.61845
226	$4.42\cdot10^{-3}$	61.9	−1.29501
229	$4.37\cdot10^{-3}$	83.1	−1.01368
232	$4.31\cdot10^{-3}$	113	−0.71935
235	$4.26\cdot10^{-3}$	164	−0.35972
238	$4.02\cdot10^{-3}$	212	−0.11568
241	$4.15\cdot10^{-3}$	310	0.25178
244	$4.10\cdot10^{-3}$	412	0.52386
247	$4.05\cdot10^{-3}$	577	0.84845
250	$4.00\cdot10^{-3}$	806	1.17062
253	$3.95\cdot10^{-3}$	1130	1.49658
258	$3.88\cdot10^{-3}$	1800	1.94258
263	$3.80\cdot10^{-3}$	3420	2.56524

The Eyring plot of ln(k/T) versus $1/T$ gave a linear fit with $R^2 = 0.9966$ (Figure S21).[S9] The activation parameters were obtainedfrom the Eyring plot using the modified Eyring equation (**1**) and the equations $\Delta H^{\neq} = -\text{slope} \cdot R$, $\Delta S^{\neq} = R \cdot (\text{intercept} - \ln(k_B/h))$, with $\ln(k_B/h) = 23.760$, and the Gibbs-Helmholtz equation (**2**). The activation parameters amount to $\Delta H^{\neq}$ = 47.3(±0.7) kJ mol^{-1}, $\Delta S^{\neq}$ = 1.39(±3.0) J K^{-1} mol^{-1} and $\Delta G^{\neq}$(235 K) = 47.0(±1.4) kJ mol^{-1}. The errors $\sigma(\Delta H^{\neq})$ and $\sigma(\Delta S^{\neq})$ were calculated on the basis of the errors of the slope (83.06708) and the intercept (0.35761) as obtained from the least-square fit. The error $\sigma(\Delta G^{\neq})$ was estimated from $\sigma(\Delta H^{\neq})$ and $\sigma(\Delta S^{\neq})$ using linear error propagation and equation (**2**).

[S7] The program *g*NMR was used for the simulation of the spectra: *gNMR*, Version 5.0.6.0, P. H. M. Budzelaar, IvorySoft, Centennial, USA, **2006**.

[S8] J. Sandström, *Dynamic NMR Spectroscopy*, Academic Press, London, **1982**.

[S9] The program Origin Pro 8G was used for the determination of the thermodynamic parameters and the Eyring plot, *Origin Pro 8G. v8.0988*, OriginLab Corporation, **2009**.

$$\ln\left(\frac{k}{T}\right) = \ln\left(\frac{k_B}{h}\right) + \frac{\Delta S^{\neq}}{R} - \frac{\Delta H^{\neq}}{R}\left(\frac{1}{T}\right) \qquad (\mathbf{1})$$

$$\Delta G^{\neq}(T_c) = \Delta H^{\neq} - T_c \cdot \Delta S^{\neq} \qquad (\mathbf{2})$$

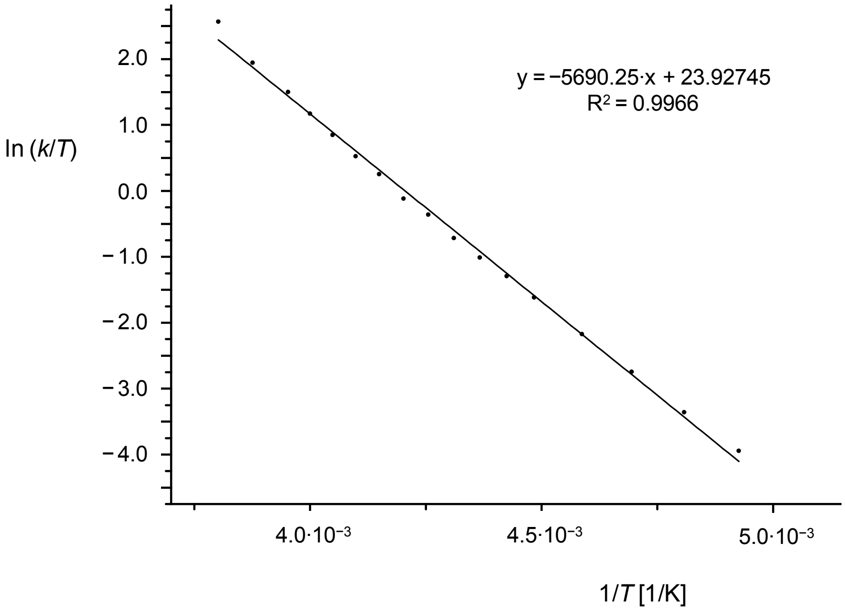

Figure S21. Eyring plot of ln(k/T) versus 1/T for the dynamic process of compound **3**·(C_6H_5F).

4. Crystal structure determination of 2-Br·0.5(n-C_6H_{14}), 2-I·0.5(n-C_6H_{14}) and 3·(C_6H_5F)

Red plate-shaped single-crystals of **2-Br**·0.5(n-C_6H_{14}) and **2-I**·0.5(n-C_6H_{14}) suitable for X-ray diffraction were obtained upon gas-phase diffusion of *n*-hexane into concentrated solutions of **2-Br** and **2-I** in benzene-d_6 at ambient temperature for one week. The synthesis of **3**·(C_6H_5F) yielded after workup (cf. Chapter 2.3) red crystalline plates of **3**·(C_6H_5F), which were suitable for X-ray diffraction.

The data collection for **2-Br**·0.5(n-C_6H_{14}) was performed on a Nonius KappaCCD diffractometer and the data collection for **2-I**·0.5(n-C_6H_{14}) and **3**·(C_6H_5F) were performed on a Bruker X8-KappaApexII diffractometer (area detector) using graphite monochromated Mo-$K\alpha$ irradiation (λ = 0.71073 Å). The diffractometers were equipped with a low-temperature device (Cryostream 600er series, Oxford Cryosystems, 123(2) K and Kryoflex I, Bruker AXS, Karlsruhe, 100(2) K). Intensities were measured by fine-slicing ω and φ-scans and corrected for background, polarization and Lorentz effects. An empirical absorption correction was applied for **2-Br**·0.5(n-C_6H_{14}), **2-I**·0.5(n-C_6H_{14}) and **3**·(C_6H_5F). The structures were solved by direct methods and refined anisotropically by the least-square procedure implemented in the SHELX program system.[S10] Hydrogen atoms were included using the riding model on the bound carbon atoms. The program Diamond 2.1c was used for the illustration of the molecular structures.[S11] CCDC numbers CCDC-1414787 (**2-Br**·0.5(n-C_6H_{14})), CCDC-1414788 (**2-I**·0.5(n-C_6H_{14})) and CCDC-1414789 (**3**·(C_6H_5F)) contain the supplementary crystallographic data for this paper, which can be obtained free of charge from the Cambridge Crystallographic Data Centre via www.ccdc.cam.ac.uk/data_request/cif.

[S10] G. M. Sheldrick, *SHELXS97* and *SHELXL97*, University of Göttingen, Germany, **1997**.

[S11] K. Brandenburg, DIAMOND 2.1c, Crystal Impact GbR, Bonn, Germany, **1999**.

Table S2: Crystal data and refinement.

	**2-Br**·0.5(*n*-C_6H_{14})	**2-I**·0.5(*n*-C_6H_{14})	**3**·(C_6H_5F)
Empirical formula	$C_{57}H_{79}Br_2N_4Si_2$	$C_{57}H_{79}I_2N_4Si_2$	$C_{84}H_{77}BF_{21}IN_4Si_2$
Moiety formula	$C_{54}H_{72}Br_2N_4Si_2$, 0.5(C_6H_{14})	$C_{54}H_{72}I_2N_4Si_2$, 0.5(C_6H_{14})	$C_{54}H_{72}IN_4Si_2$, $C_{24}BF_{20}$, C_6H_5F
Formula weight	1036.24 g mol^{-1}	1130.22 g mol^{-1}	1735.39 g mol^{-1}
Temperature	123(2) K	100(2)	100(2)
Wavelength	0.71073 Å	0.71073 Å	0.71073 Å
Crystal system, space group	monoclinic, *C2/c*	monoclinic, *C2/c*	monoclinic, *P2₁/n*
Unit cell dimensions	*a* = 38.860(2) Å	*a* = 38.872(2) Å	*a* = 19.902(2) Å
	b = 15.6626(9) Å	*b* = 15.6801(9) Å	*b* = 20.202(2) Å
	c = 22.731(2) Å	*c* = 22.785(1) Å	*c* = 20.207(2) Å
	α = 90°	α = 90°	α = 90°
	β = 125.774(3)°	β = 125.507(2)°	β = 99.189(4)°
	γ = 90°	γ = 90°	γ = 90°
Volume	11225(1) $Å^3$	11305(1) $Å^3$	8020(1) $Å^3$
Z, Calculated density	8, 1.226 mg m^{-3}	8, 1.328 mg m^{-3}	4, 1.437 mg m^{-3}
Absorption coefficient	1.525 mm^{-1}	1.193 mm^{-1}	0.525 mm^{-1}
F(000)	4376	4664	3536
Crystal size	0.36 × 0.10 × 0.02 mm	0.60 × 0.22 × 0.06 mm	0.25 × 0.17 × 0.06
θ-range for data collection	2.83 – 28.00°	1.58 – 28.00°	1.45 – 28.00°
Limiting indices	−47 ≤ *h* ≤ 47	−51 ≤ *h* ≤ 51	−26 ≤ *h* ≤ 25
	−19 ≤ *k* ≤ 19	−20 ≤ *k* ≤ 20	−26 ≤ *k* ≤ 26
	−28 ≤ *l* ≤ 26	−30 ≤ *l* ≤ 30	−26 ≤ *l* ≤ 26
Reflections collected / unique	56069 / 11020 [R_{int} = 0.0727]	131812 / 13632 [R_{int} = 0.0427]	113058 / 19365 [R_{int} = 0.0490]
Completeness to θ_{max}	99.8 %	99.8 %	99.9 %
Absorption correction	Empirical	Empirical	Empirical
Max. / min. transmission	0.9701 and 0.6097	0.9318 and 0.5346	0.9692 and 0.8799
Refinement method	Full-matrix least squares on F^2	Full-matrix least squares on F^2	Full-matrix least squares on F^2
Data / restraints / parameters	11020 / 75 / 629	13632 / 75 / 629	19365 / 35 / 1044
Goodness-of-fit on F^2	1.006	1.052	1.022
Final *R* indices [$I > \sigma(I)$]	R_1 = 0.0429, wR_2 = 0.0811	R_1 = 0.0281, wR_2 = 0.0644	R_1 = 0.0370, wR_2 = 0.0933
R indices (all data)	R_1 = 0.0867, wR_2 = 0.0950	R_1 = 0.0420, wR_2 = 0.0709	R_1 = 0.0652, wR_2 = 0.1040
Largest diff. peak / hole	0.656 / −0.540 e $Å^{-3}$	1.222 / −0.676 e $Å^{-3}$	1.690 / −0.757 e $Å^{-3}$
CCDC number	CCDC-1414787	CCDC-1414788	CCDC-1414789

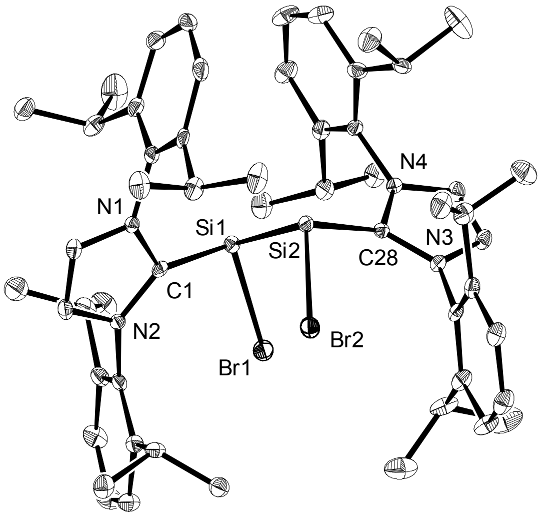

Figure S22. DIAMOND plot of the molecular structure of **2-Br**·0.5(n-C_6H_{14}) in the single crystal; thermal ellipsoids represent 30 % of the electronic propability at 123(2) K;hydrogen atoms and n-hexane molecules were omitted for clarity reasons. Selected bond lengths [Å], bond angles [°] and torsion angles [°]: C1–Si1 1.940(3), Si1–Br1 2.3602(8), Si1–Si2 2.385(1), Si2–Br2 2.3677(9), Si2–C28 1.936(3); C1-Si1-Br1 102.22(9), C1-Si1-Si2 97.87(9), Br1-Si1-Si2 103.78(4), Si1-Si2-Br2 104.17(4), Si1-Si2-C28 96.74(9), Br2-Si2-C28 101.42(9); C1-Si1-Si2-C28 161.5(1), Br1-Si1-Si2-Br2 −46.81(4).

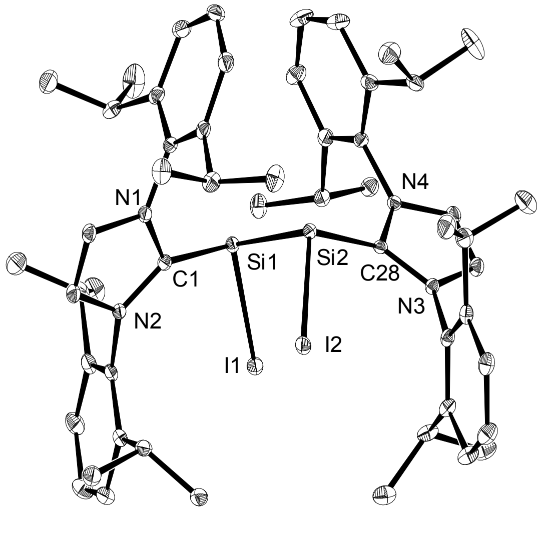

Figure S23. DIAMOND plot of the molecular structure of **2-I**·0.5(n-C_6H_{14}) in the single crystal; thermal ellipsoids represent 30 % of the electronic propability at 100(2) K;hydrogen atoms and n-hexane molecules were omitted for clarity reasons. Selected bond lengths [Å], bond angles [°] and torsion angles [°]: C1–Si 1.943(2), Si1–I1 2.6036(6), Si1–Si2 2.3909(9), Si2–I2 2.5919(6), Si2–C28 1.939(2); C1-Si1-I1 103.42(7), C1-Si1-Si2 97.04(7), I1-Si1-Si2 103.90(3), Si1-Si2-I2 103.45(3), Si1-Si2-C28 97.94(7), I2-Si2-C28 102.56(7); C1-Si1-Si2-C28 −160.31(9), I1-Si1-Si2-I2 50.46(3).

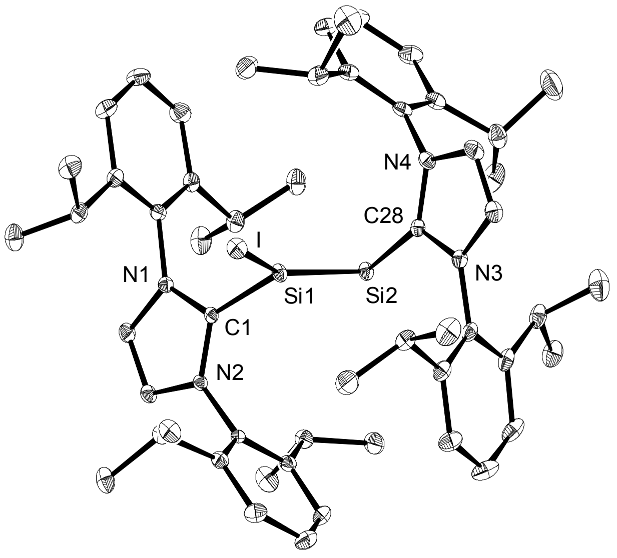

Figure S24. DIAMOND plot of the molecular structure of the cation of **3**·(C_6H_5F) in the single crystal. The thermal ellipsoids represent 30 % of the electronic propability at 100(2) K. The hydrogen atoms are omitted for clarity. Selected bond lengths [Å], bond angles [°] and torsion angles [°]: C1–Si1 1.901(2), Si1–I 2.4654(7), Si1–Si2 2.1739(9), Si2–C28 1.931(2); C1-Si1-I 104.56(7), C1-Si1-Si2 112.83(7), I-Si1-Si2 142.27(3), Si1-Si2-C28 96.61(7); C1-Si1-Si2-C28 −178.5(1), I-Si1-Si2-C28 −6.71(9).

4.1 Correlation of the Si–Si bond length in base-stabilized Si(I) compounds to the sum of bond angles at the silicon atoms

Table S3: Geometrical parameters of selected Si(I) bis(silylene) compounds. Formal charges were not considered in the formulas for simplicity reasons.

	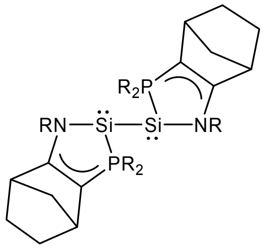 	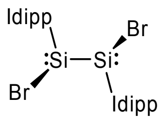	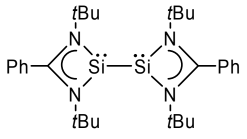	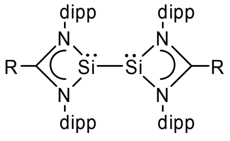
	R = dipp (N), *t*Bu (P)			R = C_6H_4-4-*t*Bu
d(Si–Si)	2.331(1) Å	2.385(1) Å	2.413(2) Å	2.489(2) Å
Σ(Si)[a]	330°	303.1°	282.4°	267.5°
Ref.	[S12]	this paper	[S13]	[S14]

[a]: The average value of the sums of angles of the two silicon atoms is given for each compound.

[S12] D. Gau, R. Rodriguez, T. Kato, N. Saffon-Merceron, A. de Cózar, F. P. Cossío, A. Baceiredo, *Angew. Chem. Int. Ed.* **2011**, *50*, 1092; *Angew. Chem.* **2011**, *123*, 1124-

[S13] C. Jones, S. J. Bonyhady, N. Holzmann, G. Frenking, A. Stasch, *Inorg. Chem.* **2011**, *50*, 12315.

[S14] S. S. Sen, A. Jana, H. W. Roesky, C. Schulzke, *Angew. Chem. Int. Ed.* **2009**, *48*, 8536; *Angew. Chem.* **2009**, *121*, 8688.

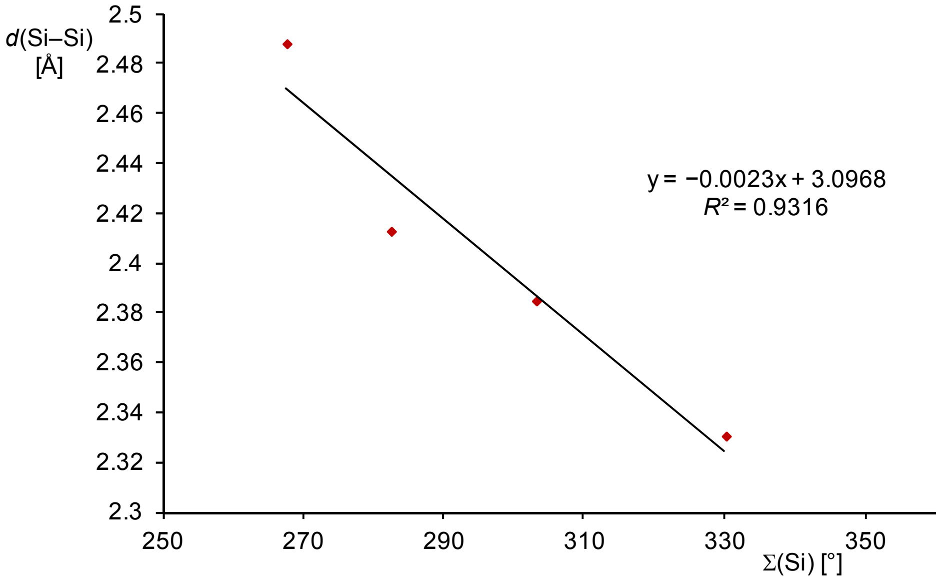

Figure S25. Plot of the Si–Si bond lengths of the Si(I) compounds depicted in Table S3 versus the sums of bond angles at the silicon atoms (average values).

5. Electronic structure calculations

Structure optimizations were performed without symmetry restraints using the ORCA 3.0.0 programm package or with symmetry restraints using the Turbomole 6.6 programm package, with their internal standard convergence criteria.[S15,S16] The B97-D3[S17] functionals, including the COSMO-solvation model[S18] for THF and RI-JCOSX approximations (ORCA) or RIJ approximations (Turbomole)[S19,S20] were employed in combination with the def2-TZVP basis set for the Si, N and carbene C atoms, and the def2-SVP basis sets for all peripherical carbon and all hydrogen atoms.[S21] Relativistic effects were approximated for iodine by the ZORA method[S22] in combination with the def2-ZORA-TZVP basis set.[S23] The level of theory employed using the ORCA program was abbreviated with B97-D3/**I** and that using the Turbomole program with B97-D3/**II**. The optimized geometries were verified as minima on the potential energy surface by two-sided numerical differentiation of the analytical gradients to obtain harmonic frequencies, which were also used to calculate the zero point vibrational energies (ZPVE). NBO and NRT analyses were performed using NBO6.0.[S24] The cartesian coordinates of the solid state structures of **2-Br** and **3** were used as a starting point for the structure optimization. A relaxed potential energy surface scan was performed involving a decrease of the Si2-I distance from 445 to 239 pm in twelve steps to obtain a starting point for the search of the transition state and the other minimum structure $\mathbf{3'}_{\mathbf{calc}}$ (π-bonded isomer). The obtained transition state $\mathbf{3}^{\mathbf{TS}}_{\mathbf{calc}}$ reveals one imaginary frequency at −92 cm^{-1}, which corresponds to a rocking vibration of the iodine atom interconverting $\mathbf{3}_{\mathbf{calc}}$ and the C_2-symmetric π-bonded isomer $\mathbf{3'}_{\mathbf{calc}}$.

[S15] F. Neese, *WIREs Comput. Mol. Sci.* **2012**, *2*, 73.

[S16] a) R. Ahlrich, M. Bär, M. Häser, H. Horn, C. Kölmel, *Chem. Phys. Letters* **1989**, *162*, 165; b) TURBOMOLE Version 6.6, University of Karlsruhe and Forschungszentrum Karlsruhe, **1989** – **2014**.

[S17] a) S. Grimme, S. Ehrlich, L. Goerigk, *J. Comp. Chem.* ***2011***, *32*, 1456; b) S. Grimme, J. Antony, S. Ehrlich, H. Krieg, *J. Chem. Phys*. **2010**, *132*, 154104.

[S18] A. Klamt, G. Schürmann, *J. Chem. Soc., Perkin Trans. 2* **1993**, 799.

[S19] a) F. Neese, *J. Comput. Chem.* **2003**, *24*, 1740; b) F. Neese, F. Wennmohs, A. Hansen, U. Becker, *Chem. Phys.* **2009**, *356*, 98.

[S20] F. Weigand, *Phys. Chem. Chem. Phys.* **2002**, *4*, 4285.

[S21] a) A. Schäfer, H. Horn, R. Ahlrichs, *J. Chem. Phys*. **1992**, *97*, 2571; b) F. Weigend, R. Ahlrichs, *Phys. Chem. Chem. Phys.* **2005**, *7*, 3297

[S22] C. van. Wüllen, *J. Chem. Phys*. **1998**, *109*, 392.

[S23] D. Pantazis, X. Y. Chen, C. R. Landis, F. Neese, *J. Chem. Theory Comput.* **2008**, *4*, 908.

[S24] NBO 6.0 Program. E. D. Glendening, J. K. Badenhoop, A. E. Reed, J. E. Carpenter, J. A. Bohmann, C. M. Morales, C. R. Landis, F. Weinhold, Theoretical Chemistry Institute, University of Wisconsin, Madison, **2013**.

5.1 Comparison of selected experimental and calculated bonding parameters of 2-Br, (*S*,*S*)-2-Br$_{calc}$ and (*R*,*S*)-2-Br$_{calc}$

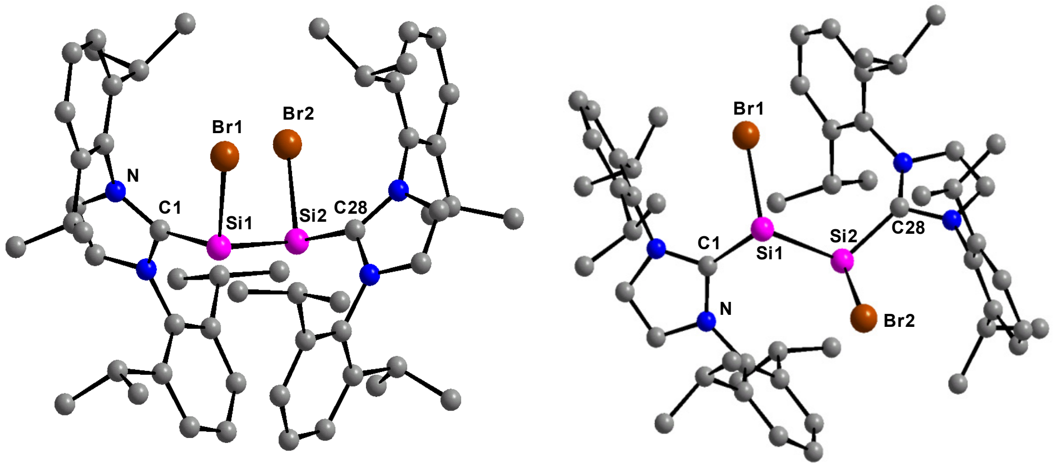

0.00 kJ mol^{-1}
(*S*,*S*)-**2-Br$_{calc}$**

57.4 kJ mol^{-1}
(*R*,*S*)-**2-Br$_{calc}$**

Figure S26. Calculated structures of the (*S*,*S*) (left) and (*R*,*S*) (right) diastereomeres of **2-Br** on the B97-D3/**I** level of theory. The relative energies are given below the structures. The H atoms are omitted for clarity. Atom numbering of the experimental structure was taken over in the calculated structures.

Table S4: Comparison of selected experimental bond lengths and angles of **2-Br** with the calculated bond lengths and angles of (*S*,*S*)-**2-Br$_{calc}$** and (*R*,*S*)-**2-Br$_{calc}$**.

	Si1–Si2 [Å]	Si–C [Å]	Si–Br [Å]	C-Si-Si [°]	Si-Si-Br [°]	C-Si-Br [°]	Σ(Si) [°]	Br-Si-Si-Br [°]	C-Si-Si-C [°]
**2-Br**	2.385(1)	1.940(3)<br>1.936(3)	2.3602(8)<br>2.3677(9)	97.87(9)<br>96.74(9)	103.78(4)<br>104.17(4)	101.42(9)<br>102.22(9)	303.9(1)<br>302.3(1)	−46.81(4)	161.5(1)
(*S*,*S*)-**2-Br$_{calc}$**[a]	2.413	1.937<br>1.938	2.405<br>2.406	97.77<br>97.43	103.83<br>103.97	102.49<br>103.29	304.09<br>304.69	-49.81	160.90
(*S*,*S*)-**2-Br$_{calc}$** C_2[b]	2.413	1.950	2.409	99.42	104.09	103.51	307.2	-48.66	164.54
(*S*,*S*)-**2-Br$_{calc}$** C_1[b]	2.405	1.948<br>1.941	2.412<br>2.410	97.83<br>99.32	103.92<br>104.01	104.11<br>103.16	305.9<br>306.5	-49.70	163.20
(*R*,*S*)-**2-Br$_{calc}$**[a]	2.469	1.963<br>1.945	2.381<br>2.393	101.21<br>115.27	107.53<br>89.69	98.42<br>102.61	307.16<br>307.57	147.64	146.49
(*R*,*S*)-**2-Br$_{calc}$** C_1[b]	2.470	1.981<br>1.944	2.384<br>2.397	100.93<br>116.76	107.99<br>91.26	97.83<br>103.66	306.8<br>311.7	149.13	145.17
(*R*,*S*)-**2-Br$_{calc}$** C_i[b]	2.511	2.034	2.407	106.91	93.91	98.30	299.1	180.0	180.0

[a]: The calculations were performed on the B97-D3/**I** level of theory. [b]: The calculations were performed on the B97-D3/**II** level of theory.

Geometry optimization of (*S,S*)-**2-Br** was carried out using the ORCA program at the B97-D3/**I** level of theory (vide supra) and gave a C_1-symmetric structure with bonding parameters very close to those of a C_2 symmetric structure. The geometry optimization of (*S,S*)-**2-Br** was repeated using the Turbomole program package at the B97-D3/**II** level of theory with and without a symmetry restriction to C_2 to elucidate the difference in energy of the two structures. The two structures were found to be isoenergetic suggesting that the

stereoisomer (*S,S*)-**2-Br** has a C_2-minimum structure. In comparison, two minimum structures of different symmetry (C_1 and C_i) resulted for the (*R*,*S*)-**2-Br** stereoisomer from the quantum chemical calculations at the B97-D3/**II** level of theory using the Turbomole program package. The C_i-symmetric minimum structure was found to be less stable by 32.6 kJ mol^{-1} than the C_1-symmetric structure. The calculated energy difference between the C_1-symmetric minimum structures of the (*S,S*) and the (*R,S*) stereoisomers was found to be 57.4 kJ mol^{-1} (ORCA, B97-D3/**I**) and 48.8 kJ mol^{-1} (Turbomole, B97-D3/**II**), respectively.

5.2 Comparison of selected experimental and calculated bonding parameters of 2-I, 2-I$_{calc}$, 3, 3$_{calc}$, 3$^{TS}_{calc}$ and 3'$_{calc}$

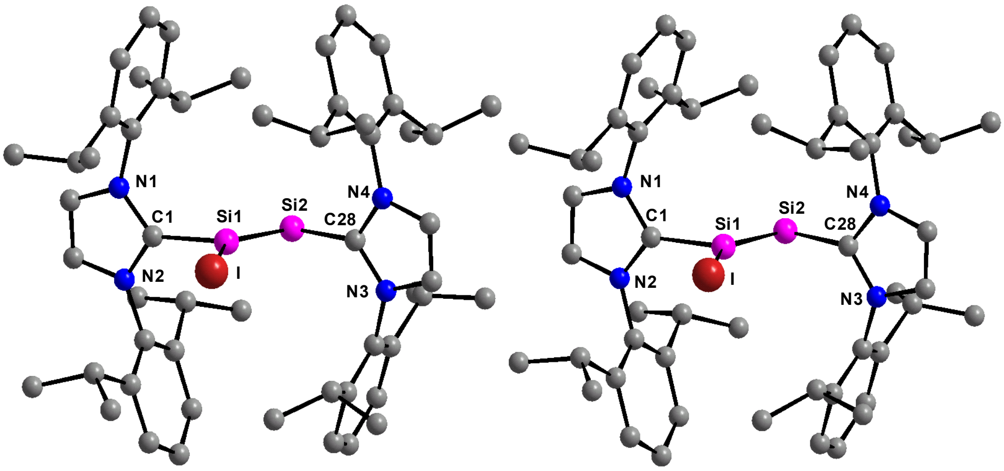

3 **3$_{calc}$, 0.00 kJ mol^{-1}**

Figure S27. Experimental (**3**) and calculated (**3$_{calc}$**, B97-D3/**I**) structures of $[Si_2(I)(Idipp)_2]^+$. The relative Gibbs energy of **3$_{calc}$** is given below the structure. The H atoms are omitted for clarity. Atom numbering of the experimental structure was taken over in the calculated structure.

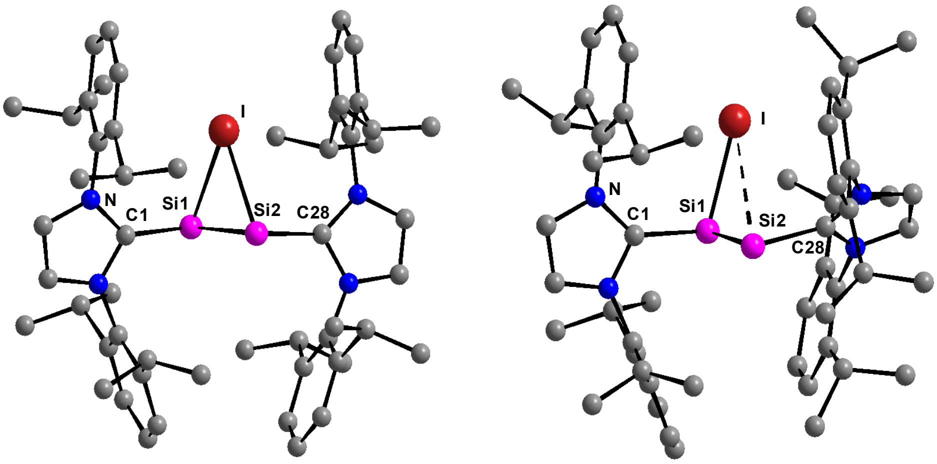

3'$_{calc}$, 18.5 kJ mol^{-1} **3$^{TS}_{calc}$, 37.6 kJ mol^{-1}**

Figure S28. Calculated (B97-D3/**I**) structures of the "π- isomer" of $[Si_2(I)(Idipp)_2]^+$ (**3'$_{calc}$**) and the transition state of the dynamic process (**3$^{TS}_{calc}$**) with their corresponding relative Gibbs energies. The H atoms are omitted for clarity.

Table S5: Comparison of selected experimental bond lengths and angles of **2-I** and **3** with the calculated (B97-D3/I) bond lengths and angles of **2-I**$_{\mathbf{calc}}$, **3**$_{\mathbf{calc}}$, **3**$^{\mathbf{TS}}_{\mathbf{calc}}$ and **3'**$_{\mathbf{calc}}$. Atom numbering of the experimental structures was taken over in the calculated structures.

	Si1–Si2 [Å]	Si1–C1 [Å]	Si2–C28 [Å]	Si–I [Å]	C1-Si1-Si2 [°]	C1-Si1-I [°]	Si1-Si2-C28 [°]	I-Si1-Si2 [°]	φ_{NHC1}[a] [°]	φ_{NHC2}[a] [°]
**2-I**	2.3909(9)	1.943(2)	1.939(2)	2.6036(6)<br>2.5916(6)	97.04(7)	103.42(7)	97.94(7)	103.90(9)	54.43(8)	125.8(1)
**2-I**$_{\mathbf{calc}}$	2.409	1.937	1.937	2.659[b]	97.50	104.16	97.47	105.08	55.6	124.5
**3**	2.1739(9)	1.901(2)	1.931(2)	2.4654(7)	112.83(7)	104.56(7)	96.61(7)	142.27(3)	96.69(7)	95.78(7)
**3**$_{\mathbf{calc}}$	2.171	1.903	1.923	2.502	112.06	103.58	96.96	144.35	89.10	89.68
**3**$^{\mathbf{TS}}_{\mathbf{calc}}$	2.366	1.950	1.936	2.618<br>3.440	95.39	104.66[b]	97.44	87.14[b]	91.98	45.56
**3'**$_{\mathbf{calc}}$	2.463	1.977	1.975	2.696	101.49	98.24[b]	101.79	62.84[b]	78.45	81.02

[a]: The dihedral angles φ_{NHC1} and φ_{NHC2} are the respective angles between the least-square plane of the atoms C1, Si1, Si2 and C28 and the respective NHC central ring planes. [b] The corresponding angles C28-Si2-I and I-Si2-Si1 are 76.12° and 49.48° (**3**$^{\mathbf{TS}}_{\mathbf{calc}}$) and 98.24° and 62.84° (**3'**$_{\mathbf{calc}}$).

5.3 Results of the natural bond orbital (NBO) and natural resonance theory (NRT) analyses of [SiBr$_2$(Idipp)]$_{\mathbf{calc}}$, (*S*,*S*)-2-Br$_{\mathbf{calc}}$, 2-I$_{\mathbf{calc}}$, 3$_{\mathbf{calc}}$ and 3'$_{\mathbf{calc}}$

Table S6: Selected results of the natural bond orbital (NBO) and natural resonance theory (NRT) analyses of [SiBr$_2$(IDipp)]$_{calc}$ (B97-D3/I). Atom numbering of the experimental structure (see ref. [25]) was taken over in the calculated structure.[a]

NBO analysis					NPA partial charges[b]		NRT analysis[c]	
	occ.	pol. [%]	hyb.	WBI				tot / cov / ionic
σ(Si–C1)	1.95	20.3 (Si1)<br>79.7 (C1)	sp$^{10.8}$ (Si)<br>sp$^{1.36}$ (C1)	0.66	C1<br>Σ(NHC)	0.08<br>0.27	Si1–C1	1.00 / 0.40 / 0.60
σ(Si–Br1)	1.96	22.0 (Si1)<br>78.0 (Br1)	sp$^{16.7}$ (Si)<br>sp$^{5.3}$ (Br1)	0.75	Br2	−0.44	Si2–Br1	0.99 / 0.43 / 0.56
σ(Si–Br2)	1.98	22.8 (Si1)<br>77.2 (Br2)	sp$^{14.9}$ (Si)<br>sp$^{4.8}$ (Br2)	0.82	Br1	−0.43	Si1–Br2	1.00 / 0.45 / 0.55
n(Si)	1.94		sp$^{0.24}$		Si	0.60		

[a]: occ.: occupancy, pol.: polarization, hyb.: hybridization, WBI: Wiberg bond index, tot / cov / ionic: total bond order / covalent bord order / ionic bond order. [b]: Partial charges obtained by natural population analysis (NPA). [c]: A local NRT analysis was carried out including the Si1, Si2, Br1, Br2, N and C1 atoms.

S25 A. C. Filippou, O. Chernov, G. Schnakenburg, *Angew. Chem. Int. Ed.* **2009**, *48*, 5687; *Angew. Chem.* **2009**, *121*, 5797

Table S7: Selected results of the natural bond orbital (NBO) and natural resonance theory (NRT) analyses of (*S*,*S*)-**2-Br$_{calc}$** (B97-D3/**I**). Atom numbering of the experimental structure was taken over in the calculated structure.[a]

NBO analysis					NPA partial charges[b]		NRT analysis[c]	
	occ.	pol. [%]	hyb.	WBI				tot / cov / ionic
σ(Si1–Si2)	1.76	50.0 (Si1)<br>50.0 (Si2)	$sp^{7.3}$ (Si1)<br>$sp^{7.3}$ (Si2)	0.93	Si1<br>Si2	0.34<br>0.35	Si1–Si2	0.92 / 0.89 / 0.03
σ(Si1–C1)	1.95	21.3 (Si1)<br>78.7 (C1)	$sp^{8.4}$ (Si1)<br>$sp^{1.2}$ (C1)	0.83	C1<br>Σ(NHC1)	0.03<br>0.10	Si1–C1	1.16 / 0.42 / 0.73
σ(Si2–C28)	1.95	21.3 (Si2)<br>78.7 (C28)	$sp^{8.4}$ (Si2)<br>$sp^{1.2}$ (C28)	0.83	C28<br>Σ(NHC2)	0.02<br>0.15	Si2–C28	1.16 / 0.42 / 0.73
σ(Si1–Br1)	1.97	21.6 (Si1)<br>78.5 (I1)	$sp^{20.0}$ (Si1)<br>$sp^{4.0}$ (Br1)	0.72	Br1	−0.47	Si1–Br1	0.99 / 0.43 / 0.57
σ(Si2–Br2)	1.97	21.6 (Si1)<br>78.5 (I2)	$sp^{20.0}$ (Si2)<br>$sp^{4.1}$ (Br2)	0.72	Br2	−0.47	Si2–Br2	1.00 / 0.43 / 0.57
n(Si1)	1.76		$sp^{0.37}$					
n(Si2)	1.76		$sp^{0.37}$					

[a]: occ.: occupancy, pol.: polarization, hyb.: hybridization, WBI: Wiberg bond index, tot / cov / ionic: total bond order / covalent bord order / ionic bond order. [b]: Partial charges obtained by natural population analysis (NPA). [c]: A local NRT analysis was carried out including the Si1, Si2, Br1, Br2, N, C1 and C28 atoms.

Table S8: Selected results of the natural bond orbital (NBO) and natural resonance theory (NRT) analyses of **2-I$_{calc}$** (B97-D3/**I**). Atom numbering of the experimental structure was taken over in the calculated structure **2-I$_{calc}$**.[a]

NBO analysis					NPA partial charges[b]		NRT analysis[c]	
	occ.	pol. [%]	hyb.	WBI				tot / cov / ionic
σ(Si1–Si2)	1.77	50.0 (Si1)<br>50.0 (Si2)	$sp^{7.00}$ (Si1)<br>$sp^{7.00}$ (Si2)	0.96	Si1<br>Si2	0.27<br>0.27	Si1–Si2	0.93 / 0.91 / 0.03
σ(Si1–C1)	1.95	20.8 (Si1)<br>79.2 (C1)	$sp^{8.83}$ (Si1)<br>$sp^{1.20}$ (C1)	0.81	C1<br>Σ(NHC1)	0.02<br>0.11	Si1–C1	1.06 / 0.41 / 0.65
σ(Si2–C28)	1.95	20.8 (Si2)<br>79.2 (C28)	$sp^{8.83}$ (Si2)<br>$sp^{1.20}$ (C28)	0.81	C28<br>Σ(NHC2)	0.02<br>0.11	Si2–C28	1.06 / 0.41 / 0.65
σ(Si1–I1)	1.96	25.5 (Si1)<br>74.5 (I1)	$sp^{27.2}$ (Si1)<br>$sp^{5.0}$ (I1)	0.78	I1	−0.38	Si1–I1	0.98 / 0.50 / 0.48
σ(Si2–I2)	1.96	25.5 (Si1)<br>74.5 (I2)	$sp^{27.2}$ (Si2)<br>$sp^{5.0}$ (I2)	0.78	I2	−0.38	Si2–I2	0.98 / 0.50 / 0.48
n(Si1)	1.77		$sp^{0.35}$					
n(Si1)	1.77		$sp^{0.35}$					

[a]: occ.: occupancy, pol.: polarization, hyb.: hybridization, WBI: Wiberg bond index, tot / cov / ionic: total bond order / covalent bord order / ionic bond order. [b]: Partial charges obtained by natural population analysis (NPA). [c]: A local NRT analysis was carried out including the Si1, Si2, I1, I2, N, C1 and C28 atoms.

Table S9: Selected results of the natural bond orbital (NBO) and natural resonance theory (NRT) analyses of **3**$_{\mathbf{calc}}$ (B97-D3/**I**). Atom numbering of the experimental structure was taken over in the calculated structure **3**$_{\mathbf{calc}}$ (cf. Figure S27).[a]

NBO analysis					NPA partial charges[b]		NRT analysis[c]	
	occ.	pol. [%]	hyb.	WBI				tot / cov / ionic
σ(Si1–Si2)	1.90	62.0 (Si1)<br>38.1 (Si2)	sp$^{0.69}$ (Si1)<br>sp$^{7.03}$ (Si2)	1.81	Si1<br>Si2	0.30<br>0.18	Si1–Si2	1.95 / 1.55 / 0.41
π(Si1–Si2)	1.89	58.1 (Si1)<br>41.9 (Si2)	p (Si1)<br>p (Si2)					
σ(Si1–C1)	1.95	24.3 (Si1)<br>75.8 (C1)	sp$^{3.85}$ (Si1)<br>sp$^{1.39}$ (C1)	0.72	C1<br>Σ(NHC1)	0.05<br>0.41	Si1–C1	1.00 / 0.47 / 0.53
σ(Si2–C28)	1.93	21.8 (Si2)<br>78.3 (C28)	sp$^{8.50}$ (Si2)<br>sp$^{1.28}$ (C28)	0.76	C28<br>Σ(NHC2)	0.06<br>0.28	Si2–C28	1.03 / 0.43 / 0.61
σ(Si1–I)	1.96	34.5 (Si1)<br>65.5 (I)	sp$^{3.87}$ (Si1)<br>sp$^{5.62}$ (I)	0.89	I	−0.18	Si1–I	0.93 / 0.64 / 0.30
n(Si2)	1.77		sp$^{0.29}$		Σ(Si$_2$I)	0.30		

[a]: occ.: occupancy, pol.: polarization, hyb.: hybridization, WBI: Wiberg bond index, tot / cov / ionic: total bond order / covalent bord order / ionic bond order. [b]: Partial charges obtained by natural population analysis (NPA). [c]: A local NRT analysis was carried out including the Si1, Si2, I, N, C1 and C28 atoms.

Table S10: Selected results of the natural bond orbital (NBO) and natural resonance theory (NRT) analyses of **3'**$_{\mathbf{calc}}$ (B97-D3/**I**). Atom numbering of the experimental structure was taken over in the calculated structure **3'**$_{\mathbf{calc}}$ (cf. Figure S28).[a]

NBO analysis					NPA partial charges[b]		NRT analysis[c]	
	occ.	pol. [%]	hyb.	WBI				tot / cov / ionic
σ(Si1–Si2)	1.82	50.0 (Si1)<br>50.0 (Si2)	sp$^{9.77}$ (Si1)<br>sp$^{9.85}$ (Si2)	0.89	Si1<br>Si2	0.26<br>0.27	Si1–Si2	0.95 / 0.94 / 0.01
σ(Si1–C1)	1.95	21.3 (Si1)<br>78.7 (C1)	sp$^{9.66}$ (Si1)<br>sp$^{1.31}$ (C1)	0.76	C1<br>Σ(NHC1)	0.03<br>0.26	Si1–C1	1.03 / 0.42 / 0.61
σ(Si2–C28)	1.95	21.2 (Si2)<br>78.8 (C28)	sp$^{9.66}$ (Si2)<br>sp$^{1.31}$ (C28)	0.76	C28<br>Σ(NHC2)	0.02<br>0.26	Si2–C28	1.03 / 0.42 / 0.61
σ(Si1–I)	1.96	21.1 (Si1)<br>78.9 (I)	sp$^{44.4}$ (Si1)<br>sp$^{9.59}$ (I)	0.66	I	−0.05	Si1–I	0.98 / 0.41 / 0.57
σ(Si2–I)	1.96	21.1 (Si1)<br>78.9 (I)	sp$^{44.0}$ (Si1)<br>sp$^{9.56}$ (I)	0.66			Si2–I	0.98 / 0.41 / 0.57
n(Si1)	1.89		sp$^{0.26}$		Σ(Si$_2$I)	0.48		
n(Si2)	1.89		sp$^{0.26}$					

[a]: occ.: occupancy, pol.: polarization, hyb.: hybridization, WBI: Wiberg bond index, tot / cov / ionic: total bond order / covalent bord order / ionic bond order. [b]: Partial charges obtained by natural population analysis (NPA). [c]: A local NRT analysis was carried out including the Si1, Si2, I, N, C1 and C28 atoms.

5.4 Cartesian coordinates [Å] and SCF energies of the calculated structures of $[SiBr_2(Idipp)]_{calc}$, (*S*,*S*)-2-Br_{calc}, (*R*,*S*)-2-Br_{calc}, 2-I_{calc}, 3_{calc}, 3^{TS}_{calc} and 3'$_{calc}$

$[SiBr_2(Idipp)]_{calc}$
Energy = −6598.877231226162 E_H

C	-0.00388554006313	-0.22402498528890	0.40651057865451
C	2.49965049615308	-2.67167485996436	0.04108207027528
C	2.97975178267035	-3.60420970368916	1.16036222303663
C	2.68239341599164	-3.31659171938679	-1.34469610168300
C	2.45279360695725	-0.12837990797312	-0.14275607865548
C	2.22432568928150	2.41963653479780	-0.33107241528206
C	3.17037263343221	-1.31156870530982	0.08391635351156
C	2.20822937136444	3.29341776823981	0.93361008804020
C	3.03203360416375	1.14986013942067	-0.11682656400420
C	2.72799678361945	3.20836609765224	-1.55047894330699
C	4.53837010739748	-1.18253954149527	0.34076421846992
C	4.40268741050195	1.22201656203398	0.15060428390211
C	5.14780789531224	0.06885412080801	0.37620363262333
C	-5.07653346008838	0.48812462635709	0.84718364996282
C	-4.68065640816468	-0.76971223642560	0.40593736755333
C	-4.16038802603171	1.53526144176417	0.90283529968702
C	-3.51965481168948	-3.53057827498860	0.34928363956025
C	-3.36074607060717	-1.01589405274494	0.00980815700151
C	-3.35397801520716	-2.56027648693284	-1.97595865424639
C	-2.83069068330985	1.34968294535103	0.51721267962921
C	-2.94779214062101	-2.38646071138768	-0.49992915835821
C	-2.46398614852323	0.06354747528714	0.07415008825206
C	-1.95223007430295	3.35189299750895	1.82172502193111
C	-2.00091715738611	3.37875690216831	-0.71683737786867
C	-1.84807710470711	2.50950712870556	0.54382001318925
C	-0.75340970190392	-0.05519921317527	-1.73032054899775
C	0.60239577645468	-0.11512977598172	-1.77557986346646
H	2.35613467893406	-4.50352350110521	1.18252667220554
H	2.16217186945013	-4.27968939496580	-1.38688252018135
H	1.18906781372780	2.13710768285761	-0.53986322273830
H	1.29788789731624	-0.08110121843075	-2.59849187496160
H	2.89742434139362	-3.11280952611740	2.13345504205752
H	2.28159675381268	-2.67787794842540	-2.13981609843802
H	1.56884445671759	4.16973856790496	0.77826742141459
H	1.83487854832490	2.73151640895259	1.79424756813447
H	2.10061088068698	4.09051145926486	-1.71809981203261
H	4.01774895287589	-3.92285798428189	1.01063399408128
H	2.70784908887582	2.59296802488035	-2.45722688787046
H	3.74426435960491	-3.48968099567739	-1.55496208407170
H	3.21479496037107	3.64938610795264	1.17821918409301
H	3.75755446954539	3.55080596709125	-1.40219960510122
H	5.12987622672651	-2.07141499102640	0.53359954377340
H	4.88736235646278	2.19345735512094	0.19411174359550
H	6.21097607376630	0.14587946596485	0.58967238880561
H	-6.10552820189990	0.65379641131354	1.15557282038568
H	-5.40423187268580	-1.57808867493782	0.37454684911933
H	-4.48213572761733	2.50948817775686	1.25618500983138
H	-4.60831397372163	-3.60800614867441	0.24636367104331
H	-4.44328932055892	-2.49995331219383	-2.08490420906448
H	-3.27290226330746	-3.39571596539649	1.40485278623045
H	-2.89008700360641	3.91628625939469	1.86634682036174
H	-3.09092485942880	-4.48265139152330	0.01834695586261
H	-2.99521406994489	3.83796092080302	-0.75335350578124
H	-3.02844789702215	-3.53837173314626	-2.34780739669383
H	-2.91148671694813	-1.79140804947881	-2.61682812202534
H	-1.88279648718199	2.71489396552363	2.70906522072934
H	-1.87027026496867	2.78606177949196	-1.62891539285522
H	-1.85729713613578	-2.45217023237376	-0.43181101075406
H	-1.13042700185936	4.07557830180704	1.85432221287687

H	-1.25393424803038	4.18095338521677	-0.72038864115445
H	-1.49431812931909	0.03631806666883	-2.50709330204995
H	-0.83737363810104	2.09371691144774	0.53203980271506
H	1.42868611433940	-2.52255295514519	0.20304997596847
N	1.04493070285264	-0.20855161935399	-0.46252334840323
N	-1.10767565355984	-0.12126048364243	-0.39280102062692
Si	-0.29667942387850	-0.24216356763503	2.41095126011834
Br	1.93844012586486	-0.01771545585763	3.15656676977713
Br	-0.52362991256761	-2.63554393537566	2.38047058221248

(*S*,*S*)-2-Br$_{calc}$
Energy = −8153.5305 E_H

C	2.29099330863490	0.10360758217179	-0.64555079652386
C	-2.31541326838456	0.10399536245982	0.65167374563185
C	-1.70849804561756	1.45386433951730	5.03944329056234
C	4.33925648264210	-0.11840966588164	3.38796786113222
C	0.69652409252125	1.21952341636107	4.33132603305106
C	4.66500633647992	-2.76766093481309	2.11487879183568
C	4.45137979913186	-4.06496756444010	1.65331922184078
C	-0.75280154225832	1.30765654725312	3.84051997521823
C	-3.50160522161102	-3.75008716929159	2.78846542704186
C	4.48983827618036	-0.24570471409008	1.86769672648014
C	4.26363650762143	-1.66021702696297	1.36245866744089
C	-1.47971563059566	-4.50396990840278	1.47393729588991
C	3.81834207537243	-4.27976002200691	0.43287901693088
C	-2.55354613031867	-3.42050157757592	1.62187588114904
C	5.85220350434250	0.29933646482850	1.40497745551203
C	-0.98440231173004	2.43048842819725	2.84265727614715
C	-0.34484335229617	3.66600872097849	2.98779577647584
C	-3.80672502456379	0.78314973036035	2.24666640166437
C	3.64436783688596	-1.91148151351501	0.12441778068508
C	3.39759207305616	-3.20483740565562	-0.35959419526861
C	-4.28139793496637	-0.36582774515154	1.71524429299320
C	-1.90029804512750	2.30374600424474	1.78238189036110
C	-3.32771153324379	-3.19500672111414	0.33606889310793
C	2.97366124158539	3.65049804569722	1.50242146849989
C	-0.59095837927852	4.71338374362482	2.10414885974818
C	1.57253608253559	-4.48861865179472	-1.54925625777686
C	-3.72704979324202	-4.26976014619149	-0.46576743875009
C	2.69165178529742	-3.44811115654982	-1.68155368019049
C	-3.67203407733432	-1.90581792524150	-0.09973439910588
C	3.38852083032842	3.20936833871294	0.09430605094778
C	-2.20721109486029	3.35959223932347	0.90400841197874
C	3.69049834229166	-3.85922055556786	-2.77750991972525
C	-1.51545656918619	4.56372234918700	1.07606702331779
C	4.23688910797461	-0.41166278096143	-1.72452794685673
C	4.63626327568569	3.96936621721556	-0.38883440851483
C	-4.43339508688762	-4.06135509208543	-1.64796203706535
C	-4.57315143939152	3.98355591176112	0.36254701719324
C	-4.37745486235810	-1.66120492315868	-1.28928438475810
C	-3.31352303151169	3.25039743929549	-0.13161898702599
C	2.26908546813776	3.34861800933171	-0.92378764861932
C	3.76644076875847	0.72839477004758	-2.27722750449385
C	-4.75238524828548	-2.76926689478113	-2.05621668264774
C	1.91167454687293	2.30079004639785	-1.79316562717142
C	1.61887512772812	4.57628210568664	-1.09164876742548
C	-6.22493662267962	0.03812924580312	-1.49668310782978
C	-4.73502463578862	-0.25530211201302	-1.73872865583769
C	-2.88650991580259	3.74429928011144	-1.51837277003550
C	0.99354611417980	2.45948897067432	-2.84690043390661
C	0.68900845006484	4.75668610496952	-2.10997984412527
C	-4.34126082376731	0.00249911630264	-3.19970681020250
C	0.39773609070625	3.71703473778420	-2.98946375454590
C	0.71716473916546	1.34520890227954	-3.84285759807026
C	-0.74088407931438	1.29658378064255	-4.31176858880416

C	1.65937200252650	1.46821881664599	-5.05522530326576
H	-1.55712192672820	0.62989444305112	5.74648226526910
H	5.16257326897052	-0.60538592222964	3.92304070261764
H	0.82872998183295	0.30788641455555	4.92291659356578
H	5.14245213536564	-2.61594403996073	3.07739111340043
H	4.77056189854543	-4.91249223493107	2.25494132615563
H	3.39518004183698	-0.55560098334964	3.72360701940577
H	-1.51811273977815	2.39607140047720	5.56863854299491
H	0.96114986102367	2.07126561825531	4.96875480992897
H	-2.93088007881363	-3.87370019810374	3.71642438018401
H	4.34409249020344	0.94041878376211	3.66826486243625
H	-2.75739376978234	1.44891093283409	4.73019544257446
H	-0.84648772569053	-4.52507645885644	2.36719644885071
H	1.38742384430068	1.17887421418418	3.48527608379621
H	-0.98149227461608	0.36290496874164	3.33543276389764
H	-4.23978267331107	-2.95779459736632	2.94712343816860
H	-4.04378198995644	-4.68378009794583	2.59432802388929
H	6.66693196198514	-0.30394979692848	1.82278714141080
H	3.64185719353237	-5.29630103231835	0.09357054366797
H	-1.92025751536591	-5.50064105620573	1.35420681665586
H	0.37132244306262	3.80498101022643	3.79150250132857
H	-2.03179662950686	-2.48930804269965	1.85888682289736
H	5.98400165680917	1.33293360230634	1.74596872146438
H	3.71020888853379	0.38573142831461	1.43022931757672
H	-4.20675109435165	1.43619742609243	3.00406334859921
H	-0.84151272519853	-4.29348895315694	0.61276759207642
H	-5.17814929750279	-0.92797546424011	1.91622600339880
H	3.78911160092186	3.45643941833834	2.20868866248748
H	5.94123206404699	0.28809247110576	0.31470044296164
H	1.97247255617115	-5.49074709512841	-1.35669844088952
H	2.09257833687205	3.09092863465381	1.82535125161401
H	-3.47487516870770	-5.28311998841641	-0.16854654960285
H	0.89351625733297	-4.21788678367106	-0.73762037495463
H	-0.05933999370507	5.65407276228922	2.22202014967091
H	4.17247106224375	-4.81089493933366	-2.52237793161591
H	2.74201029213607	4.72066251179154	1.53845037203894
H	3.65568096501117	2.15298351901896	0.15510781005720
H	5.46348532032898	3.82872592379368	0.31718485129295
H	-4.90186776603026	3.59665020407545	1.33304280911509
H	0.99242961664805	-4.53299427845721	-2.47730481046854
H	2.21809915221760	-2.51028044022821	-1.98456964552988
H	4.47587979432791	-3.10886783381000	-2.91188561367892
H	5.12194585272272	-0.99120884373646	-1.92721082555490
H	-3.56888791280941	2.19433894386271	-0.23664093242229
H	3.17271121027402	-3.98685783943349	-3.73519340673314
H	-1.71188312621632	5.39289915434462	0.40348019535936
H	-4.73116721834123	-4.91092899553476	-2.25764091855773
H	-6.48903780532725	-0.09926128792927	-0.44290595877133
H	4.43332202004742	5.04371137342888	-0.46759469632560
H	4.95999553812755	3.61215267527171	-1.37258877377291
H	-4.38017960854838	5.05710927399149	0.47306618259657
H	1.85517105564886	5.39978847770007	-0.42481625750663
H	-4.15581622100840	0.44528515984414	-1.13371863704979
H	-5.39311189229451	3.85459337832510	-0.35427237779817
H	4.16315046626398	1.36026788193857	-3.05405549460406
H	-6.85394582313013	-0.62971917197718	-2.09589796087002
H	-5.29539055109762	-2.61858588547988	-2.98491901142772
H	-6.46146935198886	1.07147420107591	-1.77715982842527
H	-1.98811911972346	3.21655251408233	-1.84461381402986
H	-2.68047438282643	4.82027303919619	-1.51968878090140
H	0.92727336452641	0.39339751497120	-3.34292864992642
H	-3.68578762150521	3.55258764166997	-2.24382942401424
H	0.19012725785277	5.71549285682825	-2.22507852432590
H	-3.28448274651290	-0.23033793185310	-3.35483284030801
H	-1.41746191405935	1.26258139289671	-3.45416019620988
H	-4.93879793709601	-0.60006866471276	-3.89178020781340
H	-4.50369659854089	1.05755344998261	-3.44811201058469

H	2.71193891989758	1.43226363013198	-4.76080731349231
H	-0.31810919897054	3.88108015980627	-3.78858305847332
H	-0.90299634672367	0.39493557031410	-4.91150326639015
H	-0.99658436871979	2.16117241672993	-4.93546282271598
H	1.47353150315944	0.65123046426143	-5.76199334177817
H	1.48892590573848	2.41740405337510	-5.57865594751432
N	-2.60546724172212	1.05967964147188	1.60623577333675
N	-3.36735458822911	-0.77901791810399	0.74801065912126
N	3.33723889877735	-0.78818228227366	-0.72829832368959
N	2.57971704609233	1.03536710020348	-1.62354776006427
Si	0.95218204681769	0.43078666742318	0.71508503243120
Si	-0.98535585602342	0.41375480717894	-0.72319647777309
Br	0.84080942384704	-1.68448025767454	1.85457818305130
Br	-0.85218295990043	-1.70694037274835	-1.85175677907527

(*R*,*S*)-2-Br$_{c\ alc}$
Energy = −8153.5086 E_H

C	0.87067319420411	-2.35145811928146	-0.15538917632346
C	5.09668301847684	-0.78555598779151	-0.41855845897350
C	3.99065334046175	1.46651698925658	-0.18569505304182
C	3.80356300096910	-0.04585238903993	-0.02515902997699
C	1.99217568777489	-4.29144227228756	-4.39514360527516
C	0.38191682849145	-2.40440268588287	-4.88948499518602
C	0.95656872442089	-3.32132409569675	-3.80192624037099
C	3.36620958158158	-0.45402612555496	1.37163974696133
C	3.85101755164034	0.21414205346893	2.50030693557959
C	2.82569648397352	-3.50822205401228	0.12291132249087
C	2.06366978426037	-4.21541043645640	-0.73952162551958
C	2.53729727049259	-1.57015930243008	1.59246263634271
C	-0.13533747736576	-4.07468162108964	-3.06311472179901
C	3.53605384760573	-0.21895612205026	3.78591167155734
C	-1.12556034171748	-4.75983164245183	-3.77473967302311
C	-0.20024169644010	-4.11334923517590	-1.66062800937595
C	2.21274703096915	-2.03703725058666	2.87834884376871
C	2.73089184337841	-1.33629979452496	3.97217488127309
C	-2.13854324505871	-5.44870140658296	-3.11271638627194
C	2.21142494022511	-4.40920721655283	3.71719743681874
C	-1.20471983900109	-4.80650651719919	-0.96298320993251
C	1.36831514762733	-3.27924838464190	3.10373109362247
C	-2.17619634954625	-5.46863211879389	-1.72116452896422
C	-0.64917725025816	-6.23010431805967	1.03381484064129
C	-1.23166021411972	-4.88979818629918	0.55283597248389
C	0.11945230133456	-2.99012583695154	3.94760193434175
C	-2.62928824699032	-4.64357452342663	1.13407671711180
H	5.38724262879264	-0.52116475477243	-1.44203637848511
H	4.21499271787157	1.70252112344527	-1.23123404664066
H	5.91536753128622	-0.50289961263307	0.25327521053017
H	4.82394311555104	1.84033572475766	0.41943232519943
H	2.79614146104241	-3.73565750600436	-4.89103005518491
H	4.97694800383127	-1.87156817212799	-0.37015479645458
H	1.18703665540812	-1.81578433662806	-5.34266076801026
H	3.07688537551368	1.99513421695775	0.10090753828332
H	3.01227753842211	-0.35488737855839	-0.71365404804570
H	2.44127603909907	-4.92381575456725	-3.62244686366137
H	1.52253511135139	-4.94745036177154	-5.13747491627888
H	-0.09565952210557	-2.98032873086114	-5.68927900588171
H	4.48265296174444	1.08582896595486	2.37192682655206
H	1.46952013423377	-2.68309200441946	-3.07559331487874
H	3.79761578811322	-3.70666523857646	0.54232762023786
H	-0.35359762182100	-1.71605580593023	-4.46528699883475
H	2.23673465784090	-5.15248222762884	-1.24173108848177
H	-1.10976476867817	-4.74722104394201	-4.86090351248808
H	3.92022954151250	0.32250625480456	4.64660651935884
H	3.07776806372331	-4.64373040583869	3.08891653310584
H	1.01756582339414	-3.62759018884234	2.13122148623833

H	2.49002047516706	-1.66566596725777	4.97837096193140
H	-2.90068712035262	-5.97393760185658	-3.68356153586541
H	0.37316813832708	-6.37327259966669	0.66704661364571
H	2.57852258741490	-4.12850639166186	4.71033561525797
H	-0.59818332077894	-4.09032756505565	0.94323014067492
H	1.60756038531817	-5.31761256007100	3.82247976468326
H	-1.25827035281925	-7.06851842791130	0.67611072145922
H	-2.96632758209777	-6.01458630922230	-1.21468407684748
H	-0.62820492468150	-6.26310409830383	2.12934276956098
H	-0.49179678263163	-2.21931681005589	3.46946093559608
H	0.38507579500864	-2.64978348987574	4.95427772676296
H	-0.47989633120864	-3.90239451542007	4.04696449007436
H	-3.02652567279114	-3.68832262199727	0.77712556911588
H	-3.33278266697279	-5.44034756268794	0.86713795317319
H	-2.57015124075432	-4.60143027345218	2.22726867656190
N	2.10243298690217	-2.37261177705345	0.47196566449805
N	0.87407465378583	-3.51354305061223	-0.90539194759385
Si	-0.68645006041417	-1.26778284041690	0.34872893239575
Br	-1.81653729412590	-1.28504350231715	-1.74693288127573
C	-0.78464190969173	2.46251163219193	0.12521875939405
C	-4.69464981485481	3.52259579407900	-2.32928625089591
C	-4.61733745875465	1.27077956066951	-1.16302893365751
C	-3.79375796576501	2.45647965779284	-1.68841948911463
C	-4.38010160742266	3.96981749702790	1.93844639136596
C	-3.95466158616564	1.72067773976693	3.04254453212194
C	-3.33521481740298	2.88863231518096	2.26304988283025
C	-2.70619178974663	1.99397868176499	-2.64486125783628
C	-3.04960930099225	1.40671968584709	-3.86671414993443
C	-1.17132723578246	4.27132837229363	-1.23393374679662
C	-1.14106950771338	4.72817264466466	0.03669263613152
C	-1.33700193286663	2.12051946911764	-2.34811427107486
C	-2.13915906299378	3.49743046481284	2.97460711943020
C	-2.06754073565546	0.97726107768199	-4.75460391017570
C	-2.18062327559870	3.78602025320859	4.34179551005709
C	-0.95028701429429	3.79739649902245	2.28875402730674
C	-0.32746069593921	1.66218694027073	-3.20510728007505
C	-0.72096430159198	1.09955828950798	-4.42389473482621
C	-1.07814296060520	4.33910133982592	4.99197358773440
C	1.75875704530552	3.04006425264396	-3.49720473408643
C	0.18501769052904	4.32705338445239	2.91881542078644
C	1.14049747320296	1.79423572221872	-2.84197319178963
C	0.09462764331718	4.59922196290038	4.28814792735704
C	1.79480351675267	6.06543488397698	2.04608885650963
C	1.48296773623362	4.56515324368849	2.16637686872314
C	1.92984105745224	0.52579567959513	-3.18521776694987
C	2.65056606366962	3.79997715235138	2.80969587581837
H	-5.42547551521896	3.89042839515371	-1.60023237567991
H	-5.35433447326203	1.61825433173095	-0.43038776305001
H	-5.24645452136015	3.11236986762278	-3.18240637341838
H	-5.15554144179206	0.77402270854090	-1.97835254747501
H	-5.21924251565677	3.53919094996091	1.38025850165257
H	-4.10636617562382	4.37517044721768	-2.68597374060327
H	-4.74160848534233	1.25084421711159	2.44190948289643
H	-3.97007829569163	0.52835823682457	-0.68928583876924
H	-3.31387037889477	2.92031545435510	-0.82431359974424
H	-3.94227287238429	4.77220062097559	1.33422880513423
H	-4.77400924687570	4.41412783616768	2.85991422560306
H	-4.40945681811032	2.05307715352962	3.98211472467319
H	-4.09822367619761	1.28343447549828	-4.12457652486523
H	-2.97747322313944	2.47478959689317	1.31773343247881
H	-1.35171178105261	4.77495024646540	-2.16946324263720
H	-3.19310937035600	0.96858682097392	3.26895806259311
H	-1.26565682229998	5.71791966787064	0.44421989111479
H	-3.08290846596645	3.57337979179026	4.90828046357422
H	-2.35404436833508	0.53230968982382	-5.70426594034998
H	1.21854579824108	3.94695657700194	-3.20381970090538
H	1.20849770851050	1.92249410816984	-1.75333520386631

H	0.03306517044806	0.74754980218151	-5.11860659712374
H	-1.13148166859642	4.55739827949238	6.05581895510240
H	0.97725623197553	6.60462511937954	1.55596653079700
H	1.72403579980294	2.96161833818242	-4.59068947074737
H	1.36198824996235	4.16235829700418	1.15618885063002
H	2.80618715109005	3.15275909605322	-3.19335472853936
H	1.94775602701840	6.51243061260783	3.03507035340051
H	0.95548434857702	5.00753342601803	4.81009371078272
H	2.70732172041109	6.21823547383778	1.45814548722930
H	1.48991909266161	-0.34333768295225	-2.68740668352273
H	1.94416176267961	0.33297417050109	-4.26223784041473
H	2.96721548027355	0.62686312337027	-2.85615115417840
H	2.41351198612460	2.73540968593401	2.88612508943544
H	2.87433873987237	4.18228491947024	3.81243107052101
H	3.55284270045869	3.91123584213341	2.19812294248352
N	-0.98568993281630	2.89213775755561	-1.18162667776120
N	-0.92678685305683	3.63079914162515	0.86130144660652
Si	0.34520979445094	0.95857990573002	0.61871162502304
Br	0.01408606103349	0.81068310381553	2.98385099461841

2-I$_{calc}$
Energy = −17356.1086 E_H

C	2.30340874508498	0.05653295656170	-0.63895060757213
C	-2.30332141392489	0.05758184731090	0.63970144651539
C	-1.60746187121190	1.50129043815547	5.07717879967774
C	4.44158632545199	0.08891358481455	3.34690089463313
C	0.79671573706727	1.26235521385203	4.32836978281947
C	4.88765152454543	-2.60460061123217	2.16765304521936
C	4.73561179990052	-3.93609793226184	1.75895520197198
C	-0.66720831084301	1.32447888155175	3.86498047928500
C	-3.77987663843647	-3.92200673675489	2.68401523488622
C	4.57637046520179	-0.09687300168504	1.82719308903366
C	4.40103376197480	-1.54453871540022	1.38285385959510
C	-1.76102037409789	-4.64516228637048	1.32110245245823
C	4.07565021935880	-4.23285549436739	0.56104939013037
C	-2.81103117816072	-3.54244466489496	1.54575093907651
C	5.90904211716263	0.48341146214262	1.31103041225471
C	-0.92773040548772	2.41515366962852	2.83184142614553
C	-0.30235963576641	3.67136029911473	2.93672134789363
C	-3.73055123326416	0.69782526252698	2.30643960585452
C	3.75592061086370	-1.87771257705391	0.16747110311945
C	3.56289048957175	-3.20928968224909	-0.25841441147571
C	-4.24284045844073	-0.41922966811193	1.74403151773389
C	-1.86376417770298	2.24604155402729	1.78444328346316
C	-3.56152609794487	-3.20839744940514	0.26372085961894
C	2.99254581416741	3.57820719171073	1.51894997220647
C	-0.58056310512957	4.69929789054935	2.02734201871428
C	1.76080489611095	-4.64674621404660	-1.31218146868909
C	-4.07371230310677	-4.23364085579729	-0.55397884334422
C	2.81240733327105	-3.54610286628435	-1.53970393588097
C	-3.75510370860603	-1.87774740766434	-0.16467046033558
C	3.36934015933305	3.15468973075831	0.09054808763642
C	-2.21723688753799	3.28842736181805	0.89340053079979
C	3.78115278669499	-3.93026044369550	-2.67651511039182
C	-1.53502347046692	4.51241451807370	1.02181955421983
C	4.24144371077263	-0.42324985418828	-1.74464883122714
C	4.59827756361954	3.92989824530417	-0.42793362045769
C	-4.73325083554386	-3.93929789548022	-1.75268948737639
C	-4.59746657379843	3.93537194630751	0.42237603451197
C	-4.39946819727457	-1.54701375767191	-1.38101733317829
C	-3.37063386359052	3.15563771055427	-0.09424235982251
C	2.21697732172301	3.28676197900174	-0.89835216785675
C	3.72909284800858	0.69308432478171	-2.30837090798515
C	-4.88543057885897	-2.60865895477973	-2.16402158764910
C	1.86331401901201	2.24289976777461	-1.78757913138897

C	1.53601103512831	4.51109483218293	-1.02969944545097
C	-5.90638805395094	0.48195265889398	-1.31192971243570
C	-4.57453203136291	-0.10015736095130	-1.82804840184081
C	-2.99383312166867	3.57342201506674	-1.52429025245261
C	0.92747750178716	2.41051288200383	-2.83537760296972
C	0.58239727680213	4.69685970880913	-2.03621345237589
C	-4.44122463752057	0.08269752024391	-3.34826356632447
C	0.30348609449804	3.66715158581086	-2.94333523724631
C	0.66487371805822	1.31754189273567	-3.86553209754188
C	-0.79996686703493	1.25484670728345	-4.32595306586120
C	1.60287806776893	1.49141253486965	-5.07991443606381
H	-1.44331238291263	0.69482612479114	5.80886487162848
H	5.29205160450130	-0.34762135247728	3.89340326074927
H	0.95303839704140	0.36830303790580	4.94923411056134
H	5.38613549564699	-2.38821421253289	3.11365558540395
H	5.12420426075720	-4.74461977969657	2.38296010891390
H	3.51491572594640	-0.37234479564812	3.71476665366046
H	-1.41373027350731	2.46286961458700	5.58054556937711
H	1.07462926705202	2.14130230469129	4.93103101259477
H	-3.22192339293754	-4.11396105584734	3.61394951432413
H	4.40875205693091	1.16168061317266	3.58855514909633
H	-2.66655893875173	1.48517959579647	4.78467501052123
H	-1.14389696253606	-4.76478258059836	2.22404243648485
H	1.46922812452087	1.19449037260779	3.46287255092649
H	-0.89836850523319	0.35766148587039	3.39227324449017
H	-4.50785361653738	-3.12236499144649	2.88342351293284
H	-4.34376357987234	-4.83455972738568	2.43102279676572
H	6.76006450923399	-0.05980767920779	1.75163961337827
H	3.94638359742826	-5.27515214972621	0.26220060471484
H	-2.22924619689091	-5.61825936206537	1.10449318399019
H	0.42556421404174	3.84314378304131	3.73111162692726
H	-2.26086303018107	-2.64062838239207	1.85059264767592
H	6.00060503118774	1.54510947176992	1.58820687313019
H	3.75884598400512	0.48223606003204	1.37531804306909
H	-4.09643151308281	1.32871893738155	3.09958292762758
H	-1.09493392804320	-4.37867289657496	0.48969797589185
H	-5.14253984194631	-0.97523091141180	1.95021187258950
H	3.83108057500589	3.37974363597103	2.20358589343681
H	5.98633081492934	0.40932520935520	0.21695977925818
H	2.22768741886019	-5.61961744121232	-1.09174948143249
H	2.11901441725426	3.00966672572293	1.86228185961013
H	-3.94412238729905	-5.27540089198493	-0.25336676083454
H	1.09429619477733	-4.37667318337902	-0.48226708784076
H	-0.05831161879034	5.65504475927969	2.11303629249910
H	4.34320449725202	-4.84328701560716	-2.42117382352096
H	2.75552740184542	4.65148953742098	1.57764897249105
H	3.65028094062533	2.09597768541142	0.13642387819983
H	5.45283906162744	3.79337795662851	0.25323710075169
H	-4.89620089785235	3.58834317023828	1.42316390263397
H	1.14436691381564	-4.76867578395143	-2.21528594519327
H	2.26359186455511	-2.64444049080693	-1.84742047828223
H	4.51076104911039	-3.13246059037526	-2.87725062127919
H	5.14069739704435	-0.97996851627769	-1.95072057613998
H	-3.65404563979247	2.09744228246015	-0.13637106887716
H	3.22338190294109	-4.12328751008173	-3.60633331924301
H	-1.76828915948653	5.32942903590233	0.33704663781512
H	-5.12113732418373	-4.74915441381423	-2.37541576420236
H	-5.98291638111258	0.41037346295858	-0.21765163264801
H	4.38423880256615	5.00812291886083	-0.49651843688299
H	4.89685529618926	3.57889202212489	-1.42738416439008
H	-4.38105198657981	5.01336085594939	0.48723524233079
H	1.76929218888646	5.32917487551742	-0.34619893114741
H	-3.75613325596454	0.47932992413480	-1.37812083085881
H	-5.45277072223501	3.79850197873254	-0.25778179059723
H	4.09448888753303	1.32249692484337	-3.10289704945662
H	-6.75805979053373	-0.06172541642150	-1.75073141506420
H	-5.38339584831270	-2.39422112580031	-3.11074438187199

H	-5.99761353852337	1.54306445974226	-1.59141055367198
H	-2.12189743548048	3.00173405843016	-1.86632473569471
H	-2.75426251397179	4.64592742740134	-1.58676269547339
H	0.89661966923760	0.35172611835479	-3.39107340464897
H	-3.83327246087922	3.37466864465445	-2.20774812912047
H	0.06092263813557	5.65283611777039	-2.12402468341082
H	-3.51570561993774	-0.38058464490289	-3.71650874468373
H	-1.47085579364237	1.18912813264466	-3.45900634220866
H	-5.29302547787226	-0.35348338450749	-3.89299477991408
H	-4.40704237349066	1.15497916270322	-3.59187122506014
H	2.66249391592806	1.47709226694215	-4.78923008583572
H	-0.42416087658857	3.83775229118570	-3.73823660679057
H	-0.95780953224939	0.35955229787476	-4.94462411502836
H	-1.07877003879098	2.13262054725413	-4.92990156320224
H	1.43813381432317	0.68265013636839	-5.80894550935455
H	1.40756129965936	2.45134632241054	-5.58580672361241
N	-2.54822828929372	0.98364718323029	1.63587280223643
N	-3.37411821689072	-0.80284858778893	0.72345812835497
N	3.37367342832455	-0.80465811884845	-0.72238722535261
N	2.54779200307376	0.98078095479552	-1.63688689374629
Si	0.96308902558594	0.36794123487126	0.72416204678919
Si	-0.96242954562243	0.36580309227525	-0.72352802176399
I	0.83451725669098	-1.93456356396427	2.04848534891150
I	-0.83394396846879	-1.93935599920761	-2.04228587868742

3$_{\mathbf{calc}}$
Energy= −10127.9752 E_H

C	15.19192219807570	4.13550869811196	17.13426306256411
C	17.09794554359904	3.58238571775744	18.20462834976744
H	17.78470171881123	3.53040879875032	19.03325652818923
C	17.18693309720618	3.10460588299983	16.93138758707439
H	17.96741166799195	2.55419466586170	16.43256932259237
C	15.42053646915427	4.92551958207292	19.48907721680588
C	14.59889332965540	4.24517659091147	20.41734270711545
C	14.19949752039979	4.96374606037070	21.55874650419180
H	13.55132128528638	4.48552908096284	22.29293125100259
C	14.61878450230800	6.28363147308410	21.76547524564846
H	14.29615468568321	6.82110308128190	22.66008341478729
C	15.43963160491139	6.92274972101791	20.82893414094273
H	15.74900453351743	7.95417039803702	21.00021429711301
C	15.85294612433224	6.26136168385138	19.65724996193282
C	14.21300519201607	2.78245041269972	20.23321745785184
H	14.18655720673695	2.57906334799711	19.15103420503990
C	12.81504403170168	2.45433019987901	20.78005497103297
H	12.06073238420631	3.14913261051443	20.38809391195895
H	12.78498021242764	2.49815033643786	21.87907991548640
H	12.52873222234173	1.43618137674181	20.47895749089231
C	15.28244736088872	1.86065675882874	20.85933252266342
H	15.02617943125111	0.80376386353367	20.68437220842554
H	15.34282032352793	2.02408584667064	21.94689057145859
H	16.28000215858618	2.04248424928788	20.43455178285558
C	16.76017758156636	6.95562230088846	18.64605093449066
H	16.63624258040261	6.44409362889951	17.67871468254810
C	16.39309555438157	8.43387893071741	18.42420699225801
H	16.62594334246326	9.04643331044424	19.30789766881405
H	15.32616370218086	8.55033849919003	18.19371101678906
H	16.97173816320183	8.83781138386417	17.58012374244560
C	18.24105386136254	6.82592360206810	19.06355584556527
H	18.89424909391955	7.29440333362483	18.31201105579882
H	18.54755376951972	5.77712650965053	19.17620993473358
H	18.41130063069664	7.33026319319962	20.02735251204516
C	15.70854524019995	3.15689454304571	14.90481683531894
C	16.13457899256004	4.07248979722480	13.91381334812586
C	15.83184139283710	3.74888066631695	12.57779627443291
H	16.13700873717026	4.42601973184190	11.77968540059507

C	15.13150925747729	2.57920316754428	12.25867220452761
H	14.89608538389290	2.35634466306735	11.21529349038965
C	14.72531816968926	1.69682991653382	13.26701242854859
H	14.17531178321216	0.79452804262255	13.00125489254568
C	15.00965427806254	1.96166401424192	14.61872211109929
C	16.93214089184260	5.32680741579285	14.25529510760633
H	16.66561931999965	5.62273715369466	15.28156043200168
C	16.60565125074065	6.52225910781829	13.34324293209991
H	15.52397990897684	6.70440338370150	13.29323492367190
H	16.97902023177670	6.36715563721863	12.32016199666737
H	17.08881086024654	7.42958841652901	13.73476969417280
C	18.44720751263611	5.02827315281866	14.22568649246550
H	19.02001916927108	5.92168615265045	14.51818342447839
H	18.75982775916752	4.73879978022232	13.21066310369689
H	18.71921612930374	4.21120005329763	14.90717801094448
C	14.62450109223831	0.97256427097230	15.71225438072974
H	14.50230934655437	1.54150967144875	16.64761072950677
C	13.28526899283579	0.27188309009679	15.44204988745523
H	13.35286716790665	-0.43437052148579	14.60141673481886
H	12.49688315208930	1.00233823840989	15.21700583319153
H	12.98098384568199	-0.29960931595951	16.33112656518333
C	15.75192973706656	-0.06151045401183	15.92734759406071
H	16.70867137214971	0.41791103618984	16.17752814245356
H	15.90245589046871	-0.65896886310933	15.01430184668320
H	15.48906304246298	-0.74742688576123	16.74724642030463
C	10.38582272046896	4.14724332770328	16.79090467743635
C	8.50737954494418	4.73178326876772	15.67464149372670
H	7.83382925247606	4.78142608408868	14.83480775607751
C	8.40477765300223	5.22909358214613	16.93694184313219
H	7.62157865811973	5.79335676216655	17.41699259070826
C	10.13996416776923	3.20682991799786	14.50967921140889
C	9.79309002019766	1.83495806203623	14.58872566216198
C	10.18800991635895	1.00737799141119	13.52175424478535
H	9.93934965018220	-0.05398879684813	13.54626602661871
C	10.90765526447088	1.52250730438708	12.43755548360080
H	11.21580820576743	0.86025162937302	11.62497300726606
C	11.24084322279606	2.88107753011908	12.39374496784815
H	11.81024914184148	3.26405952489453	11.54727799199587
C	10.86371946700719	3.75789241226847	13.42830876716770
C	8.98533169055375	1.26761506233914	15.75071676873919
H	9.14657423332971	1.92324332045478	16.61701725820875
C	7.47636128501614	1.29216446864192	15.42743447857124
H	7.25376815394289	0.62722354528545	14.57857350106910
H	7.13479544502692	2.30340644174978	15.16604053128990
H	6.89122046886088	0.94963423102002	16.29499598774058
C	9.43638305227518	-0.14013366788961	16.17688336483462
H	8.88952926591530	-0.44753214893509	17.07928344824576
H	10.51129219370344	-0.15886745674239	16.40278354372811
H	9.23141617471207	-0.89097409913931	15.39966961087935
C	11.18715347895271	5.24346223706871	13.35204250269948
H	11.27160562821829	5.61603540861848	14.38441612278389
C	10.03917696213191	6.01274344064394	12.66109463025325
H	9.07579692355485	5.85645715041727	13.16603845142756
H	9.92901879927401	5.67834671606303	11.61745738290500
H	10.25064815445181	7.09296064458834	12.65624207679096
C	12.52343624622355	5.52995140544212	12.65073252915262
H	12.76917951979938	6.59658104644323	12.74156835619136
H	12.47753080854524	5.29123041232407	11.57811501558147
H	13.34365488438998	4.95283166579517	13.09689166092171
C	9.75142901958862	4.95395693927358	19.04536265601765
C	10.47940419374077	6.03977900627444	19.58316857884767
C	10.66903740703871	6.05875255217575	20.97689935189911
H	11.24024135592194	6.86924687266205	21.42798458495656
C	10.14312832265841	5.04994693364107	21.79471482903368
H	10.30983476051116	5.08285061953084	22.87426177440994
C	9.40603198540290	4.00168990953094	21.23412715597629
H	8.99438352807667	3.22421004492766	21.88082026051361

C	9.19647219423533	3.92680788316629	19.84370655702565
C	10.97985420265613	7.17406519952859	18.70180700040527
H	11.20484528238814	6.74663408777146	17.71351109472199
C	9.87366060033102	8.23595736353290	18.51501504490290
H	9.61994132285585	8.70026018502278	19.48111088017572
H	8.95368377825096	7.80178521355128	18.09979382908630
H	10.21700239429513	9.02678535794008	17.83080299946968
C	12.27016059366030	7.81849084270108	19.22473591356894
H	12.63757658758146	8.55743681787584	18.49954645373868
H	13.05629736931502	7.06792707087266	19.37587213827516
H	12.10916287445952	8.34458955938112	20.17735601520812
C	8.37959524910455	2.77991277674657	19.26246201127476
H	8.39287418953110	2.87066190625220	18.16864414262566
C	8.98707080055756	1.40858292974085	19.61191064817970
H	9.00208991783469	1.23982179136556	20.69885337099712
H	10.01996416814998	1.32926765482303	19.24220624914956
H	8.39379355571893	0.60324524405842	19.15476364039413
C	6.90290931362666	2.88022527437678	19.69592659662045
H	6.47142305825242	3.84979476844078	19.40386129491285
H	6.79998270180432	2.77843747445240	20.78739979119761
H	6.31109196399374	2.08345754298122	19.22045743374402
N	15.87316981408519	4.21561041607237	18.31012721010573
N	16.01444342997856	3.45671386928510	16.28870449330429
N	9.72434676430693	4.06553246545733	15.60103361737628
N	9.56032377851644	4.85775799255810	17.61194531362540
Si	13.40544605206816	4.69891745385127	16.79720819860360
Si	11.95738633499962	3.16206745974205	17.29970063254906
I	13.68183788739404	7.01003190255240	15.87849704050028

3^{TS}_{calc}
Energy= −10127.9598 E_H

C	0.102422	0.465819	2.330140
C	0.746372	1.887332	3.986091
H	1.425619	2.531746	4.519769
C	-0.541267	1.536294	4.226887
H	-1.224432	1.808017	5.015622
C	2.454859	1.283515	2.270062
C	2.823162	2.422234	1.517710
C	4.151320	2.479005	1.052851
H	4.488258	3.355545	0.496630
C	5.049243	1.433759	1.303910
H	6.070976	1.494252	0.922649
C	4.648937	0.317005	2.049776
H	5.364052	-0.481855	2.249248
C	3.344754	0.221959	2.566845
C	1.865069	3.586063	1.281889
H	0.848162	3.240640	1.521302
C	1.846191	4.060663	-0.181274
H	1.567688	3.243369	-0.860923
H	2.822253	4.457071	-0.496860
H	1.104640	4.863834	-0.304660
C	2.202405	4.754673	2.232278
H	1.469817	5.567945	2.117739
H	3.200993	5.158622	2.004257
H	2.203351	4.437649	3.285348
C	2.945475	-0.931757	3.480902
H	1.848569	-0.955381	3.534884
C	3.387255	-2.304221	2.946598
H	4.482793	-2.405941	2.929093
H	3.007397	-2.460668	1.928168
H	2.987743	-3.101850	3.590623
C	3.469812	-0.677527	4.910273
H	3.125458	-1.471033	5.592087
H	3.112780	0.287329	5.301078
H	4.571074	-0.663075	4.926031

C	-2.236144	0.081381	3.151776
C	-2.478950	-1.071813	3.930341
C	-3.765411	-1.636241	3.843887
H	-3.997500	-2.543007	4.404973
C	-4.754440	-1.053719	3.039507
H	-5.746141	-1.509518	2.986480
C	-4.483799	0.108053	2.304280
H	-5.268677	0.548314	1.687545
C	-3.208500	0.699989	2.335864
C	-1.385970	-1.706783	4.788833
H	-0.625314	-0.936515	4.985034
C	-0.681179	-2.858438	4.041479
H	-0.222685	-2.510065	3.106515
H	-1.398158	-3.652611	3.786126
H	0.110588	-3.292601	4.672060
C	-1.909187	-2.171663	6.160515
H	-1.068061	-2.494350	6.792715
H	-2.596493	-3.025714	6.064743
H	-2.441189	-1.360683	6.680440
C	-2.896196	1.964501	1.542413
H	-1.805348	2.006701	1.403145
C	-3.519788	1.965446	0.137687
H	-4.617465	2.027866	0.172974
H	-3.254739	1.058228	-0.423287
H	-3.158255	2.830513	-0.437461
C	-3.308973	3.219117	2.341449
H	-2.824115	3.244242	3.327190
H	-4.398673	3.235860	2.498652
H	-3.026152	4.133640	1.796390
C	-0.129606	-0.411588	-2.337248
C	-0.721259	-1.267298	-4.347823
H	-1.339555	-1.402649	-5.219309
C	0.482336	-1.803445	-4.012501
H	1.123804	-2.492162	-4.537061
C	-2.211913	0.494421	-3.369314
C	-1.956224	1.840079	-3.735925
C	-3.054546	2.717701	-3.771463
H	-2.898958	3.761823	-4.047190
C	-4.347730	2.268528	-3.477844
H	-5.186986	2.967069	-3.509745
C	-4.571836	0.924803	-3.160080
H	-5.586951	0.585280	-2.951891
C	-3.510591	0.001713	-3.099421
C	-0.576995	2.319210	-4.174059
H	0.165975	1.604165	-3.799318
C	-0.482098	2.304585	-5.713991
H	-1.198200	3.013903	-6.156244
H	-0.700722	1.304145	-6.115930
H	0.529770	2.589386	-6.040541
C	-0.204920	3.693960	-3.592719
H	0.844551	3.930349	-3.825823
H	-0.325412	3.699251	-2.499907
H	-0.822573	4.499504	-4.017511
C	-3.788034	-1.471354	-2.827870
H	-2.853527	-1.925616	-2.469763
C	-4.197913	-2.188711	-4.133350
H	-3.437530	-2.082301	-4.919861
H	-5.138883	-1.770131	-4.522571
H	-4.350389	-3.262698	-3.947280
C	-4.855640	-1.693910	-1.740604
H	-4.894893	-2.759611	-1.470883
H	-5.858002	-1.406309	-2.091585
H	-4.632802	-1.121588	-0.830164
C	2.098479	-1.522789	-2.123711
C	2.285199	-2.758507	-1.466121
C	3.527209	-2.969940	-0.836820
H	3.710036	-3.910785	-0.315976

C	4.515931	-1.982158	-0.841885
H	5.461078	-2.154764	-0.323227
C	4.298555	-0.767099	-1.503741
H	5.082995	-0.011031	-1.496662
C	3.089825	-0.510224	-2.175292
C	1.211570	-3.839577	-1.431726
H	0.248591	-3.362375	-1.666429
C	1.483516	-4.918266	-2.501749
H	2.432147	-5.439097	-2.295677
H	1.551899	-4.488739	-3.511122
H	0.676930	-5.667605	-2.502675
C	1.067605	-4.482242	-0.039705
H	0.179395	-5.130267	-0.017094
H	0.950241	-3.715537	0.739333
H	1.940124	-5.104073	0.212593
C	2.884948	0.784568	-2.962846
H	1.854394	1.131927	-2.772514
C	3.813000	1.926719	-2.530655
H	4.857370	1.730146	-2.817167
H	3.777936	2.083590	-1.446681
H	3.506644	2.860325	-3.023640
C	3.019004	0.539450	-4.481406
H	2.303792	-0.204791	-4.851756
H	4.034205	0.183741	-4.715488
H	2.850737	1.474542	-5.036155
N	1.126036	1.227842	2.826787
N	-0.921177	0.667300	3.210009
N	-1.079592	-0.408763	-3.321039
N	0.834066	-1.272256	-2.780357
Si	0.478486	-0.727666	0.834545
Si	-0.131233	0.822315	-0.845616
I	-1.796254	-1.934980	0.362175

3'$_{calc}$
Energy= −10127.9657 E_H

C	-2.51028943195118	0.33916324015187	-0.05489606859975
C	-4.45745859119418	1.45116240089874	-0.46153260959258
H	-5.10475205625831	2.31180917459132	-0.50871832526728
C	-4.66064054142469	0.14905671571937	-0.77779902936485
H	-5.52518197177955	-0.37119636331961	-1.15758449418467
C	-2.55938314398370	2.79239421347263	0.39665758217797
C	-1.92759037180611	3.58584542409642	-0.58762741594636
C	-1.46140023478054	4.85038908644923	-0.18437899573684
H	-0.97009981507399	5.49967476969528	-0.90956128788421
C	-1.64217950598472	5.29513806786456	1.13278375547075
H	-1.28205917526529	6.28406036878005	1.42395191307557
C	-2.27869956422318	4.48309582290480	2.08055411938040
H	-2.40793730935237	4.84459062678060	3.10213548783892
C	-2.75032507901329	3.20334506092418	1.73535213449293
C	-1.85397553164470	3.13290476099135	-2.04077426435818
H	-2.03737369656461	2.04848034967100	-2.06461301629155
C	-0.47392005199611	3.36352841868295	-2.66763917739847
H	0.31409481982090	2.89677936766255	-2.06428967408085
H	-0.23961479524454	4.43290566421125	-2.76190670592964
H	-0.43772767599178	2.92307539602717	-3.67489677337831
C	-2.96640638653649	3.81379274992994	-2.86640059971659
H	-2.95931831635306	3.43904560842325	-3.90161049962330
H	-2.81312015379853	4.90389366356607	-2.89495893114942
H	-3.96167908837927	3.62328677018988	-2.43913616493622
C	-3.48483289969218	2.33317494382192	2.74956787019100
H	-3.63371819662758	1.34180111540095	2.29648733234900
C	-2.67302035669381	2.11590167865405	4.03949751255872
H	-2.47821380447244	3.06431754509595	4.56198218695602
H	-1.70946623782515	1.63693398275395	3.81787732424050
H	-3.23125416425283	1.46221145639566	4.72667564703261

C	-4.88109207802304	2.91887372620501	3.04973955752847
H	-5.43968304256664	2.25232388689338	3.72468464151289
H	-5.46730109465027	3.04735000632776	2.12748037743822
H	-4.79696556911299	3.90301312703316	3.53659075313920
C	-3.35794144647427	-1.93462806361263	-0.76703350915467
C	-3.62770367052364	-2.81500382082564	0.30281912489617
C	-3.55583979174213	-4.19328924661379	0.02907502897443
H	-3.75168756781912	-4.91262617704708	0.82681644368703
C	-3.23124594861613	-4.65566927024632	-1.25445004032169
H	-3.17979376117952	-5.73045280632203	-1.44659403734334
C	-2.96912588175727	-3.75058099666293	-2.29156585061260
H	-2.70903234130202	-4.12838561986430	-3.28231597859287
C	-3.02109022391886	-2.36248323607946	-2.06907696121779
C	-3.99038189303300	-2.30325277766663	1.69248404091124
H	-3.73416260280248	-1.23451314471228	1.73510810195054
C	-3.18414318397343	-2.99445552707148	2.80699079405859
H	-2.10275760571017	-2.90330086408448	2.62771748699358
H	-3.43176287131701	-4.06359075610321	2.88842231098624
H	-3.41111345763316	-2.52818473103591	3.77810091241559
C	-5.50951019320840	-2.42429485978191	1.93166793286348
H	-5.77965306730366	-2.01037334332472	2.91527769397656
H	-5.82389009808131	-3.47917098219122	1.90689218421044
H	-6.07969870495053	-1.88165959456493	1.16353817745607
C	-2.72168934300593	-1.37574512440356	-3.19254420608909
H	-2.62336691477651	-0.37544916473710	-2.74417138033394
C	-1.38784568517030	-1.67858434150786	-3.90175367999948
H	-1.41311821576514	-2.65027763669620	-4.41783091707174
H	-0.55147434153572	-1.68790429662400	-3.18759924511768
H	-1.18107074852449	-0.90518804736954	-4.65676054212863
C	-3.89230393355173	-1.32553144656895	-4.19573355691163
H	-4.83842165736728	-1.06436316933282	-3.69859843262984
H	-4.02847298356091	-2.30318072765868	-4.68416580980085
H	-3.69736470417207	-0.57741694758393	-4.97901809194896
C	2.53690552614101	0.06542584228830	0.07179837039721
C	4.67060904553395	-0.35252482661961	0.74745800853702
H	5.48594714591533	-0.96486700939256	1.09719336077512
C	4.58676275419681	0.97352822621160	0.48145266342028
H	5.31398058260069	1.76723782003224	0.54007301884327
C	3.17237139877819	-2.29890127203987	0.71417028478680
C	3.29651873456043	-3.18102422194220	-0.38101572252942
C	3.10296894762370	-4.55039738390118	-0.12312081929300
H	3.18394680976060	-5.27014583718319	-0.93990437300627
C	2.79884696332327	-5.00337486202233	1.16890903551309
H	2.64890338845462	-6.07113676829064	1.34772604757986
C	2.67767319778454	-4.09689359199307	2.23025591199611
H	2.42699126435421	-4.46553860401303	3.22701417487890
C	2.85768209171701	-2.71672959241982	2.02499243664418
C	3.62625500646620	-2.67504990831097	-1.78088191951971
H	3.40868499737761	-1.59677975329590	-1.80529999221803
C	5.12916409948011	-2.85156474253583	-2.08173751703083
H	5.40311831560793	-3.91788874750477	-2.07350262216444
H	5.75114328840998	-2.33541936593620	-1.33637620905934
H	5.37396331432601	-2.44308581149175	-3.07431627386970
C	2.75179029963767	-3.32419336357570	-2.86846062559480
H	2.94019307534805	-2.84014765225822	-3.83895954761761
H	1.68284270977986	-3.21481041403686	-2.63408475327895
H	2.97119571397086	-4.39618462250553	-2.98532009477721
C	2.70013857314520	-1.72582810411750	3.17319416850612
H	2.71187988224969	-0.71241817281489	2.74539362920871
C	3.88621944182581	-1.82780047010591	4.15353546881637
H	4.84681062006288	-1.67027930123897	3.63999177305651
H	3.91802293045283	-2.82097689865821	4.62728971902552
H	3.79355806804645	-1.07318782021892	4.94948665109929
C	1.35211813474697	-1.88762627071584	3.90106949088863
H	1.24149779757055	-1.10737278677243	4.66940324817485
H	1.28025295413794	-2.86402023390939	4.40361865317912
H	0.51013601698789	-1.79758262057789	3.20004716777467

C	2.81756074816107	2.51616926105024	-0.30598194089597
C	2.25908393917226	3.33308615991494	0.70346514339249
C	1.91189060375335	4.64696675653402	0.34039479507203
H	1.47815440692130	5.31468561212868	1.08476300375733
C	2.13818982813497	5.11541025552894	-0.96135045120030
H	1.86983259082428	6.14168961280489	-1.22083668948621
C	2.70182393064951	4.27975859051715	-1.93406216540683
H	2.86478881713706	4.66026112003525	-2.94392969711451
C	3.05112586950630	2.95094730556150	-1.63038355047086
C	2.14457403906956	2.84500502641086	2.14272114573767
H	2.22141092803417	1.74769367580732	2.13272034943084
C	3.32151312756338	3.38687397566933	2.98262951017971
H	3.27980236586012	4.48544227496065	3.04245521284679
H	4.29219080751776	3.10748931149014	2.54745402573609
H	3.27789486628920	2.98547775258197	4.00678438230738
C	0.79530883492158	3.18920408105795	2.78448709225540
H	0.72240424988462	2.73045058116087	3.78149085704477
H	-0.03512019220736	2.81046638494840	2.17656922425616
H	0.66098290069784	4.27319115173473	2.90464083009119
C	3.69948907528199	2.04995276429955	-2.67587843411484
H	3.78392325056067	1.04105908659393	-2.24634665355831
C	2.84214077023537	1.92181532277918	-3.94894782787120
H	2.69554935252190	2.89589930399290	-4.43895453357897
H	1.85562212845845	1.49971438583408	-3.71245355833590
H	3.33740371290819	1.25301371082897	-4.66915976381403
C	5.12874840795696	2.53459493852184	-2.99761137759302
H	5.74104295821599	2.60443460951130	-2.08599355555980
H	5.10966763931726	3.52979218981142	-3.46831923540602
H	5.62193732369745	1.83943188678567	-3.69415305895772
N	-3.14717916174697	1.54869628974100	-0.02364204596677
N	-3.46840400871926	-0.51896551508537	-0.52699105493657
N	3.41718117468610	-0.89640164323454	0.49511047934899
N	3.28531750534967	1.20986303512888	0.07166221892632
Si	-0.81031475225591	0.14241816498557	0.93473992804289
Si	0.82140398163809	0.04748466359926	-0.90725709904269
I	-0.11698715889925	-2.29959904877742	0.03013652569285
